# Domestication Effects on Stress Induced Steroid Secretion and Adrenal Gene Expression in Chickens

**DOI:** 10.1038/srep15345

**Published:** 2015-10-16

**Authors:** Amir Fallahsharoudi, Neil de Kock, Martin Johnsson, S. J. Kumari A. Ubhayasekera, Jonas Bergquist, Dominic Wright, Per Jensen

**Affiliations:** 1 AVIAN Behavioural Genomics and Physiology Group, IFMBiology, Linköping University, 58183 Linköping, Sweden; 2 Department of Chemistry – Biomedical Center, Analytical Chemistry and Science for Life Laboratory, Uppsala University, Box 599, BMC, SE-75124, Uppsala, Sweden

## Abstract

Understanding the genetic basis of phenotypic diversity is a challenge in contemporary biology. Domestication provides a model for unravelling aspects of the genetic basis of stress sensitivity. The ancestral Red Junglefowl (RJF) exhibits greater fear-related behaviour and a more pronounced HPA-axis reactivity than its domesticated counterpart, the White Leghorn (WL). By comparing hormones (plasmatic) and adrenal global gene transcription profiles between WL and RJF in response to an acute stress event, we investigated the molecular basis for the altered physiological stress responsiveness in domesticated chickens. Basal levels of pregnenolone and dehydroepiandrosterone as well as corticosterone response were lower in WL. Microarray analysis of gene expression in adrenal glands showed a significant breed effect in a large number of transcripts with over-representation of genes in the channel activity pathway. The expression of the best-known steroidogenesis genes were similar across the breeds used. Transcription levels of acute stress response genes such as *StAR*, *CH25* and *POMC* were upregulated in response to acute stress. Dampened HPA reactivity in domesticated chickens was associated with changes in the expression of several genes that presents potentially minor regulatory effects rather than by means of change in expression of critical steroidogenic genes in the adrenal.

Domestication is the evolutionary process where organisms adapt to living with humans^1^, leading to more docile and tame animals^2^. Chickens were domesticated from the Red Junglefowl (RJF) about 8000 years ago^3^. During this period, they have undergone immense changes in morphology, physiology and behaviour. Domesticated chickens grow faster, become sexually mature earlier, lay many more and larger eggs, display an extensive variation in colour and plumage, and show reduced fearfulness and increased stress tolerance^1^,^4^,^5^,^6^,^7^,^8^. We previously reported that mature ancestral RJF show more pronounced behavioural and physiological responses to restraint stress compared to its domesticated descendant, the White Leghorn (WL)^9^. It is not known whether this is mediated by central effects in the brain, or by peripheral effects in the hormone-secreting organs. A detailed understanding of hampered HPA-axis activity will provide important knowledge about the domestication process and its stress effects in animals.

The primary physiological stress response in general is an increase in HPA-axis activity, which results in elevated levels of the glucocorticoids that regulate metabolism. Glucocorticoids are mainly synthetized in the adrenals by enzymes belonging to two major protein classes: Cytochrome P450 enzymes and hydroxysteroid dehydrogenases^10^. The synthesis of pregnenolone from cholesterol is the first step in the synthesis of all steroids. The rate-limiting process of transporting cholesterol into the mitochondria is mainly mediated through the steroidogenic acute regulatory proteins (StAR). Mitochondrial enzymes catalyse pregnenolone into various steroids including glucocorticoids. The main cytochrome P450 enzymes involved in this process are *CYP11*, *CYP17*, *CYP19*, *CYP21*, while for the hydroxysteroid dehydrogenases the principal genes are *3βHSD* and *17βHSD*. The process is controlled by ACTH secreted from the anterior lobe of the pituitary glands^11^,^12^. Being less fearful is accompanied by a decreased hypothalamic-pituitary-adrenal (HPA) axis reactivity in all species studied to date^13^,^14^,^15^,^16^,^17^,^18^. In the present experiment, we investigate the possibility that domestication may have modified the stress responses of chickens by targeting genes involved in the steroidogenic pathway in the adrenals. This should lead to modified stress levels of several steroids that could be detected by identifying altered genetic functions that are related to steroidogenesis.

Recent research suggests that regulatory mutations that cause changes in gene expression can lead to fast and dramatic evolutionary changes^19^,^20^. Considering the short evolutionary time between the divergence of the domesticated animals from their wild ancestors, it can be expected that many phenotypic variations among domesticates are mainly resulting from variations in gene expression patterns rather than protein coding mutations. Bearing in mind the difference in behaviour of domesticates and their wild counterparts, researchers have focused on brain gene expression^19^,^21^,^22^,^23^,^24^. For example, Albert, *et al.*^19^ compared brain gene expression of dogs, pigs and rabbits with their likely ancestors and found that variation in gene expression was generally larger than sequence variation between related species pairs. The number of the identified so called “domesticated genes” was also higher than the number expected by neutral fixation of gene expression over time^25^. So far, no studies have addressed the domestication-induced effects on genes involved in stress-mediated steroid synthesis.

The aim of the current study was to evaluate the effects of domestication on acute stress sensitivity of chickens at both molecular and hormonal levels. This was achieved by studying adrenal gene expression at baseline and after restraint stress in six weeks old domesticated White Leghorns and ancestral Red Junglefowl using microarrays and quantitative PCR. The plasma levels of three central steroids with adrenal origin, pregnenolone (PREG), dehydroepiandrosterone (DHEA), and corticosterone (CORT) were also compared in the same animals.

## Results

### Hormone levels

**Figure 1:**
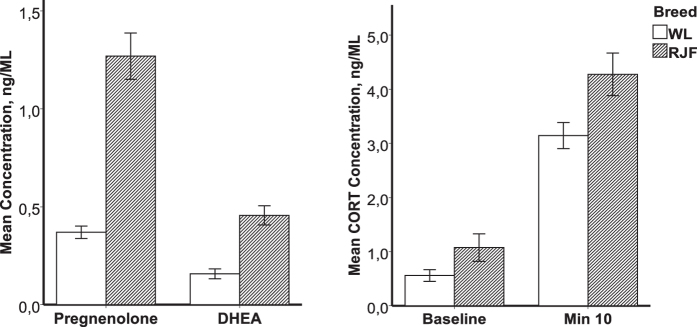
Serum concentrations of (**a**) baseline pregnenolone and DHEA and (**b**) baseline and post-restraint levels of corticosterone in domestic White Leghorn (WL; n = 24) and wild Red Junglefowl (RJF; n = 24). The values are given as mean ± SEM. The statistics for the figures is presented in Table 1.

Basal plasma levels of PREG, DHEA and CORT, as well as stress levels of CORT, were compared between With Leghorn (WL) and Red Junglefowl (RJF) birds (12 birds of each breed and sex; 48 in total) following ten minutes of physical restraint in a hanging net. The basal levels of PREG and DHEA were significantly higher in RJF (Table 1) but there was no significant difference between WL and RJF in baseline levels of CORT. Ten minutes of physical restraint led to significant elevation of the CORT levels in both WL and RJF, with the levels after restraint being higher in RJF (Fig. 1). With the exception of basal CORT, there was no significant effect of sex on the levels of the measured hormones (Table 1).

### Gene expression difference between White Leghorn and Red Junglefowl

The birds were sacrificed either immediately after being taken out of their home cages (baseline samples), or after 15 min of physical restraint in a net (stress samples). Their adrenal glands were then dissected out and snap frozen. Based on microarray analysis of pooled mRNA samples (3 individuals of each sex, breed and treatment per pool), 1291 transcripts were significantly differentially expressed when comparing the breeds in an ANOVA model that included sex and treatment (baseline or stress) as co-factors (FDR adjusted P value < 0.01) (Supplementary Table S1). Gene ontology analysis of differentially expressed genes with DAVID^26^ showed a significant (FDR adjusted P value < 0.01) enrichment in channel activity (GO: 0015267). Various GABA receptors, glutamate receptors, and transcripts related to potassium and calcium channels were among the genes differentially expressed between the breeds (Supplementary Table S2). The transcripts belonging to glutamate receptors were up-regulated in the WL. Some members of the GABA receptors and voltage-gated channels were up-regulated in WL, while other members of these families were up-regulated in RJF. With the exception of *CYP21*, the expression of other key genes known to be involved in steroidogenesis were similar in both breeds. RT-PCR confirmed the over-expression of *CYP21* in WL and similar gene expression of other steroidogenesis genes between the breeds. Breed effect were found for the expression of both *StAR* and *CH25* (Table 1).

### Gene expression affected by restraint

**Figure 2:**
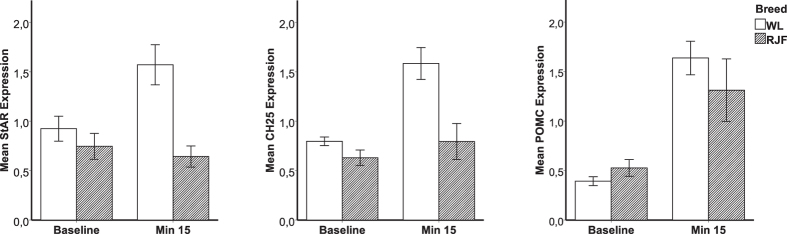
Quantitative PCR (qPCR) analysis of StAR (**a**), CH25 (**b**), and POMC (**c**) mRNA expression in adrenals of White Leghorn (WL) and Red Junglefowl (RJF). The adrenals were either collected at baseline or after 15 minutes restraint in a net. Results are shown as fold change after normalizing with TATA-binding protein and beta-2 Microglobulin mRNA. Each value represents the mean ± SEM. The statistics for the figures is presented in Table 1.

mRNA levels from adrenals were compared between breeds at baseline and after physical restraint for 15 minutes, using the same oligo microarrays as above. We found that 35 transcripts were significantly up-regulated (FDR-adjusted P value < 0.01) and three were significantly down-regulated after restraint compared to baseline levels (Supplementary Table S3). The P-values obtained for breed x treatment in the ANOVA model failed to reach statistical significance after FDR correction for multiple testing. RT-PCR results were in agreement with the results obtained from the microarrays confirming the effects of stress on the expression of *StAR*, *CH25* and *POMC* (Fig. 2a–c).

### Gene expression affected by sex

**Figure 3:**
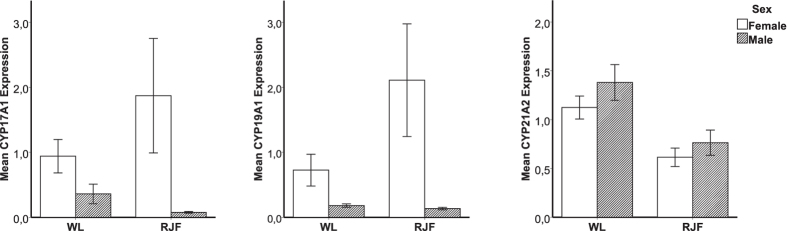
Quantitative PCR (qPCR) analysis of CYP17A1 (**a**), CYP19A1 (**b**), CYP21A2 (**c**) mRNA expression in adrenals of male and female White Leghorn (WL) and Red Junglefowl (RJF). Results are shown as fold change after normalizing with TATA-binding protein and beta-2 Microglobulin mRNA. Each value represents the mean ± SEM. The statistics for the figures is presented in Table 1.

A total of 430 transcripts were differentially expressed between the sexes (FDR adjusted P value < 0.01). *CYP17A1* and *CYP19A1*, which are several of the key genes involved in steroidogenesis, were up-regulated in females (Supplementary Table S4). No significant enrichment in any pathway was found using DAVID. Results obtained from qPCR were in agreement with the microarrays and confirmed the up-regulation of *CYP17A1* and *CYP19A1* in females (Fig. 3a,b).

## Discussion

Our results support previous observations that endocrinological stress responses, and the associated breed levels of steroid hormones, have been modified by domestication in chickens^9^. In addition, we report hundreds of differentially expressed genes in the adrenals when comparing the breeds. This suggests that domestication has targeted regulatory mechanisms of the genetic responses to stress. However, we found only limited evidence of a direct effect on the expression of genes immediately involved in the steroidogenic pathway.

Maintenance of homeostasis, gluconeogenesis, and the development of secondary sexual characteristics are amongst the functions regulated by steroid hormones^27^. The adrenal glands play a crucial role in the production of CORT and other steroids. In a previous study, the CORT response to restraint was lower in WL birds than in the ancestral RJF although the WL birds were slower to recover^9^. Here, we have investigated both the plasma levels of adrenal derived steroid hormones and the global transcription levels of central stress-related genes in the adrenal glands. Similar to our previous findings in adult birds, CORT increase due to restraint stress was higher in the RJF 10 minutes after the restraining stress. Blunted HPA axis reactivity is a widespread trait amongst all studied domesticated species, including chickens, pigs, guinea pigs, mice, rats, salmonids, ducks and silver foxes^9^,^13^,^14^,^15^,^16^,^17^,^28^,^29^. It has been suggested that the HPA axis reactivity has been modified as a consequence of selection for docility during domestication, which in turn has led to a chain of events causing shared characteristics among domesticates^2^,^29^,^30^. Destabilizing selection^31^ and the neural crest hypothesis^2^ suggest some underlying connections between adrenal function and the occurrence of shared domestication traits.

The levels of PREG and DHEA were higher in the RJF. Elevated levels of DHEA are positively associated with plasma ACTH levels, as well as with psychosocial stress in humans^32^. In line with our present results, the RJF is reported to show more fearful behaviour than WL in various behavioural tests displaying a more pronounced physiological response to stress^4^,^9^,^33^. However, it is worth noting that we have compared individuals from only one outbred WL population against a single RJF population that has lived in captivity for 14 generations. Our ongoing studies suggest that commercial strains of WL also have an attenuated HPA response to stress. The current intense selection on production traits might have altered the HPA activity of domesticates even further due to resource allocation or other factors. To conclusively state that domestication has dampened HPA activity in chickens, more populations should be studied.

A large number of transcripts were significantly differentially expressed between the breeds (Supplementary Table S1). However, although the RJF showed higher HPA-axis reactivity as measured by CORT levels after stress, the expression levels of known steroidogenic genes (with the exception of *CYP21A2*) were mostly similar across the breeds. Steroid hormones are central components of vital physiological functions such as metabolism and homeostasis^34^. Our data suggest that the attenuated HPA axis reactivity in WL is not related to changes in the transcription of crucial steroidogenic genes by the adrenal glands. Although transcript levels and gene expression are usually highly correlated, it is worth noting that potential post transcriptional differences may exist between the breeds^35^. We studied 6 weeks old chickens, with the persistence of breed difference in HPA reactivity in adults leading us to assume with some certainty that the majority of gene expression changes found would maintain a similar pattern in adulthood. Various factors such as age and environment may still partially alter tissue specific gene expression profile, however.

Factors other than gene expression may affect the endocrine stress response. Many studies have suggested that the variability of HPA axis reactivity is due to differences in the adrenal sensitivity to ACTH^36^,^37^,^38^. However the role of the central nervous system and the pituitary gland in HPA axis regulation is well known^12^. A decrease in the size of adrenals during domestication^2^ or augmented metabolic clearance rate of CORT may also be involved in the attenuated HPA axis reactivity in WL. Since the size of the adrenal glands are reduced in some domesticates^14^,^39^, the other possibility is that the RJF maintains higher hormonal levels due to larger adrenal glands or different cell composition rather than through elevated enzyme activity (something not measured in the present study).

mRNA levels of *CYP21A2* were significantly higher in WL of both sexes (Fig. 3c). This gene codes for 21-hydroxylase, which is involved in the conversion of the main steroid precursors into cortisone and aldosterone. A deficit in 21-hydroxylase activity leads to reduced levels of aldosterone and increased levels of plasma androgens^40^,^41^. The lower expression of *CYP21A2* in the RJF may partly underlie the observed higher plasma levels of DHEA and pregnenolone in this breed.

Adrenal chromaffin tissue is the main site of catecholamine biosynthesis during stress in birds^42^,^43^. Our functional analysis showed a significant overrepresentation of genes related to ‘channel activity’ pathways. Several GABA receptors, glutamate receptors, opioid receptors, serotonin receptors, dopamine receptors, and genes coding various potassium, calcium and sodium channels, were among the differentially expressed genes in the above-mentioned pathway (Supplementary Table S2). GABA and glutamate receptors are important inhibitory and excitatory receptors in the nervous system and mediate their effect by stimulating ion channels^44^,^45^,^46^. Adrenal glands also play a central role in the regulation of the fight or flight response, fearfulness and aggression by releasing catecholamines through the direct action of sympathetic nerve fibres^43^,^47^,^48^,^49^. Hence, the enrichment of differentially expressed genes in the pathway modulating production and release of catecholamines might possibly indicate that selection for tameness during domestication has had a bigger influence on the sympatico-adrenal medulla axis than the HPA-axis.

To verify that the observed gene expression reflected the state achieved after CORT levels peaked, the adrenals were sampled five minutes later than blood samples, i e, after 15 minutes. Following the restraint, various transcripts including several known genes related to immediate stress responses^27^,^50^ such as *StAR*, *POMC* and *CH25*, were up-regulated (Supplementary Table S4). However, the expression of genes specifically related to steroidogenesis (*CYP11, CYP17, CYP19, CYP21, 3βHSD* and *17βHSD*) was not affected by the restraint. This supports the suggestion that only long term exposure to stress or ACTH treatment causes up-regulation of the steroidogenic genes^11^.

Although the increased expression of steroidogenic enzymes is central for the chronic stress response, the transportation of cholesterol into mitochondria is mediated by a rapid increase in the transcription of *StAR*, which is the crucial rate-limiting step in acute hormonal response to ACTH^27^,^51^,^52^,^53^. *Cholesterol 25-hydroxylase (CH25)* also plays crucial roles in both mediating cholesterol transport into the inner mitochondrial membrane and cleaving it into Pregnenolone^51^,^54^. The stress effect on the expression of *StAR* and *CH25* seems to be more pronounced in the WL (Fig. 2a,b). CORT levels were significantly higher than baseline in both breeds after restraint. Therefore it is possible that *StAR* transcription returned to baseline levels 15 minutes after the initial handling in RJF but not in WL birds. This speculation is in agreement with our previous results where CORT levels one hour after restraint did not statistically differ from peak levels in WL when RJF had recovered to baseline^9^. The regulation of *StAR* transcription is cAMP dependant and very rapid but the exact underlying mechanism is still unclear^55^.

In birds, adrenal cortical and medullary tissues are anatomically mixed and the adrenal glands can function independent from the ACTH^56^. In mammals, however the hypothalamic-spinal-adrenocortical (HSA) axis also takes part in modulation of glucocorticoid secretion^57^,^58^, with pro-opiomelanocortin (POMC)-derived peptides being involved in all aspects of adrenal functioning^59^. Although exposure to stressors leads to an increase in the transcription of POMC in the rat pituitary^60^,^61^,^62^, we are not aware of any study indicating stress-related modulation of *POMC* transcription in the adrenals of mammals or birds. The adrenal transcription and modulation of POMC by stress implicates its potential role in the pituitary-independent responsiveness of adrenals in chicken. Gulevich, *et al.*^63^ showed that selection for tameness in foxes has significantly altered the mRNA levels of *POMC* in the anterior pituitary.

We detected 475 deferentially expressed genes between sexes. A total of 187 genes out of 194 located on the Z chromosome were up-regulated in males, while all observed 32 genes located on the W chromosome were up-regulated in females. The male biased expression differences observed in a large number of genes located on the Z chromosome can be explained by the lack of global dosage compensation in chickens^64^,^65^. The remainder of the differentially expressed genes were located on autosomes (154 genes were up-regulated in males, and 92 were up-regulated in females). Sex-biased gene expression of autosomal genes is common in many organisms^66^. Large sex differences in behaviour, brain gene expression, and to a lesser extent, DNA-methylation, exist in chickens^65^. Underlying causes and the probable physiological consequences of the observed genes with sex biased expression were not the focus of this study, however these remain interesting topics for future research. Adrenal glands are involved in the production of various steroid hormones that are important for sexual dimorphism in chickens. Hence, sex-biased gene expression in adrenals may contribute to the extensive sexually dimorphic behavioural and physiological traits observed in chicken.

Plasma levels of pregnenolone and dehydroepiandrosterone were significantly higher in the juvenile Red Junglefowl in comparison with the domesticated White Leghorn chickens, but the baseline levels of corticosterone was similar between the breeds. However, restraining stress caused a significantly larger increase in corticosterone in RJF than in WL, indicating a blunted HPA-axis reactivity in domesticated chickens. Using mRNA microarrays on adrenal tissue, we found that a large number of transcripts were differentially expressed between the breeds but the expressions of the key steroidogenic genes were not different. However, there was a significant over-representation of genes in the channel activity pathway, which may explain some of the observed differences in the plasmatic levels of the measured steroids. It also indicates that the sympatico-adrenal medulla axis may have been targeted more than the HPA-axis during chicken domestication. Our data suggest that domestication has resulted in decreased adrenal activity in WL, mediated by changes in the expression of several genes with potentially minor regulatory functions.

## Materials and Methods

### Ethical statement

All experimental protocols were approved by Linköping Council for Ethical Licensing of Animal Experiments, ethical permit no 122-10. Experiments were carried out in accordance with the approved guidelines.

### Animals and housing

Two different breeds of chicken were studied in this experiment; WL birds and their wild progenitor the RJF. The WL were the progeny of an outbred line called SLU13, developed for research and selected for egg mass. The RJFs stem from a wild population from Thailand (for details regarding the origin of the breeds, see^67^). In order to sustain genetic diversity the animals have been bred based on pedigree; however some degree of inbreeding is unavoidable in experimental populations. Twelve birds of each breed and sex (48 individuals) from 24 families were used for both the gene expression and the hormonal analysis when they were 6 weeks old. All of the birds were hatched and raised together in a 2 × 3 m pen with ad libitum access to food and 12/12 hours light/dark (for hatching and rearing routines, see^9^).

### Blood Sampling

The experiment was conducted in two consecutive days in the afternoon. To avoid disturbing the animals during sample collection and to minimize the effects of their potential social status on hormone levels and gene expressions, they were collected and kept in groups of 3 individuals per cage (size: 60 × 40 × 40 cm) with ad lib access to water and food), one day in advance. Each cage housed three individuals of the same sex and breed. All birds within each cage were blood sampled within 3 minutes after the capture of the first bird. After the initial blood sampling to measure corticosterone at basal levels, the birds were restrained in a hanging net for 10 minutes and were blood sampled again. For details regarding the restraint treatment, see Ericsson 2014^9^.

### Tissue sampling

One week after blood sampling, the same individuals were used for tissue collection. One day before sampling, the animals were caged in small groups. The subjects were divided into two groups; control and stressed (24 individuals per group including equal numbers from each sex and breed). Regarding the control group, within 3 minutes after the capture of the first bird from each cage all 3 birds were decapitated and their adrenal gland was removed surgically. The birds in the stressed group were restrained in a net for 15 minutes before decapitation and dissection of the adrenals. The adrenals were removed within 3 minutes after decapitation and were immediately snap-frozen in liquid nitrogen. The samples were stored in a −80 °C freezer before the analysis.

### Hormonal analysis

Dehydroepiandrosterone (DHEA), pregnenolone (PREG), corticosterone (CORT) and the internal standards DHEA-d_2_ and cortisol-d_4_ were purchased from Steraloids Inc. (Newport, RI). Methoxyamine hydrochloride, the internal standard PREG-^13^C_2_-d_2_, highest purity solvents and chemicals were obtained from Sigma-Aldrich (Stockholm, Sweden), unless otherwise stated.

### Steroids extraction

A slightly modified method of the steroid extraction performed by Liu, Y. *et al.* was used^68^. 200 μL of chicken plasma was mixed with the internal standard mixture. The internal standard mixture was prepared with DHEA-d_2_, PREG-^13^C_2_-d_2_ and cortisol-d_4_ in methanol. After the addition of an internal standard (IS) to plasma samples, each sample contained 1 ng of each IS. The extraction of steroids from chicken plasma was performed using 2 mL of *tert*-butyl methyl ether (MTBE) as the extraction solvent. Samples were gently vortexed for 10 min and were centrifuged at 1000 g for 10 min. The supernatant was collected and the solvent was evaporated under a stream of nitrogen gas. The dried samples were reconstituted with 100 μL methanol. During the extraction, the lipids were protected against oxidation by the addition of 0.05 mg/mL butylated hydroxytoluene (BHT) to the extraction solvent (MTBE).

### Derivatization of steroids

Extracted steroids were derivatized with methoxyamine to form methoxime derivatives. In brief, the methoxime derivatives of steroids were prepared by adding 100 μL of 20 mg/mL of methoxyamine hydrochloride. The samples were incubated at 60 °C for 45 min. The solvent was evaporated under a nitrogen stream and methoxime derivatives were dissolved in 100 μL of methanol. Samples were kept at −20 °C prior to the analysis by SCF-MS/MS as described below.

### Separation of chicken plasma steroids by supercritical fluid (SCF) chromatography

The supercritical fluid chromatography system was a Waters ACQUITY® UPC^2^™ (Milford, MA), equipped with a binary solvent delivery pump, an autosampler, a column oven and a back pressure regulator. The qualitative analysis was performed at 40 °C using an Acquity UPC^2^ BEH column (100 mm × 3.0 mm × 1.7 μm; Waters, Milford, MA, USA). The mobile phase flow rate was maintained at 3.0 mL/min with a gradient elution (eluent A, CO_2_; eluent B, methanol). The gradient program was started with 2% of component B, then, a linear gradient was programmed from 2% to 17% for 2.01 min, followed by a linear gradient down to 2% B in 3.0 min, and finally it was held for 1.0 min for the elution of ionic liquids out of the instrument. Isocratic solvent was 97.5% methanol, 2.5% water and 0.1% NH_4_OH with a flow rate of 0.4 mL/min. The back pressure was set at 1800 psi and the injection volume was 2.0 μL.

### Identification of plasma steroids by tandem mass spectrometry (XEVO® TQ-S)

Steroids were identified by using a Waters Xevo TQ-S mass spectrometer (Milford, MA, USA). The data acquisition was in the positive ion electrospray ionization (ESI) mode. The desolvation gas was nitrogen, and the collision gas was argon (0.25 mL/min). The data acquisition range was *m/z* 50–800. The capillary voltage was 2.8 kV, the cone voltage was 30.0 V, and the source offset was 30 V. The source temperature was 150 °C and the desolvation temperature was 500 °C with the desolvation gas flow rate of 750.0 L/h. The cone gas flow was 150.0 L/h. The nebulizer gas flow was at 7.0 bar. MS data were collected using two separate scan functions. The first scan function was set at low collision energy (5 eV), which provided parent ions, and the second scan function was set at high collision energy (ramped from 15 to 30 eV) which provided fragment ions of parent ions (multiple reactions monitoring (MRM)). The scan time for each function was set at 0.3 s. Data were acquired and analyzed with Waters MassLynx v4.1 software. Finally the quantification was accomplished using internal standards. The sample analysis was performed in triplicates to test the repeatability, and a CV < 10% was accepted.

### Microarray analysis

RNA was isolated from the frozen adrenal tissue using AllPrep DNA/RNA Mini Kit (Qiagen, Germany) and the potential residual DNA was digested on-column using RNase-Free DNase Set (Qiagen, Germany) during extraction according to manufacturer’s instructions. Using 6 μg of total RNA The double stranded cDNA was prepared with Maxima H Minus Double-Stranded cDNA Synthesis Kit (Thermo Fisher Scientific, USA), followed by phenol-chloroform extraction and precipitation. Two labelled dsDNA from each treatment-group-sex were pooled (8 × 3 pooled samples from each group according to treatment, sex and breed), Cy3 labelled with the NimbleGen One Colour Labeling Kit and hybridized to a Nimblegen 12 × 135 k custom gene expression arrays and scanned with a NimbleGen MS2000 scanner according to manufacturer’s protocol (Roche Nimblegen). In order to decrease the probability of dissimilar hybridization between breeds due to presence of SNPs in the probe sequences, the microarray probes were designed based on the chicken reference genome (galGal3), avoiding SNPs found in a previous resequencing of wild and domestic chickens^69^.

### RT-PCR confirmation

mRNA from the individual birds (without pooling) was used for confirmation of gene expression by means of Real-time PCR (RT-PCR). Considering the large number of differentially expressed genes, we focused only on validating the expression of the genes with known biological importance in steroidogenesis. The validated genes were: *MC2R, StAR, FDX1, POMC, CH25, CYP17A1, CYP19A1,CYP21, HSD3B2* and *HSD11B1*. We used the same double stranded cDNA as we used to perform the microarray for template in RT-PCR. Specific primers were designed using primer3^70^,^71^ and were blated against the chicken genome with the NCBI primer blat tool to avoid multiple amplification^72^. If possible the primers were selected to be on exon/intron boundaries to evade amplification of potential genomic DNA residual. The specificity of primers was confirmed by observing a single band of PCR product by gel electrophoresis and via inspecting the melting curve in Light Cycler. The reactions were performed in the Light Cycler 480 (Roche Diagnostics, Basel, Switzerland). Each reaction included 2 μl water, 1 μl of each forward and reverse primers (0.5 μM), 125 picograms (pg) cDNA diluted in 1 μl water, and 5 μl SYBR Green I Master (Roche Diagnostic). The following RT-PCR reaction was performed: 5 min 95 °C activation followed by 45 times (10 s 95 °C, 10 s 55 °C, and 20 s 72 °C). The program was ended with a melting curve from 72 °C to 95 °C and cooling down to 40 °C. The crossing point (cp) values were normalized with 2 housekeeper genes. The relative expression difference between the individuals was measured according to the method of Pfaffl^73^.

### Statistical analysis

Microarray pre-processing was performed with the RMA method^74^ using NimbleGen DEVA version 1.2.2. The Microarray statistical analysis was conducted using R (http://www.r-project.org) and Bioconductor packages (http://www.bioconductor.org). Three samples were discarded after quality control of raw expression data. 80 percent of the transcripts with low variance across arrays (SDs = 0.8) were filtered out without any prior hypothesis^75^. Differentially expressed genes were selected using a linear model approach with the Limma package^76^. Breed, treatment, and sex were included in the model as fixed factors. Benjamini and Hochberg adjusted P value (FDR cutoff 0.01) was applied to determine statistical significance^77^,^78^. We used Database for Annotation, Visualization and Integrated Discovery (DAVID) for functional analysis^26^,^79^. The hormonal and the RT-PCR result were analysed with Generalized Linear Models (SPSS v. 22.0). We assumed a normal distribution for the variance. The Initial GLM model was fitted with breed, treatment, sex, and day of the experiment as fixed factors and cage number as covariate. The effects of “day of the experiment” and “cage number” were not significant (p > 0.05) for either of the measured dependent variables, and hence were excluded from the final model. The significance levels were determined with the Wald Chi-squared test with adequate degrees of freedom. For determining the performance of the model against the intercept, the Omnibus (Likelihood Ratio Chi-Square) test was used and this was considered acceptable if the significance level was below 0.05. The P values were adjusted for multiple testing using a Bonferroni correction^80^.

## Additional Information

**Accession Codes**: Experiment Array Express accession: E-MTAB-3622

**How to cite this article**: Fallahsharoudi, A. *et al.* Domestication Effects on Stress Induced Steroid Secretion and Adrenal Gene Expression in Chickens. *Sci. Rep.* **5**, 15345; doi: 10.1038/srep15345 (2015).

## Supplementary Material

Supplementary Information

## Figures and Tables

**Table 1:** Results of the statistical analysis of hormonal variables and qPCR shown in Fig. 1, 2, 3. The analysis is based on Generalized Linear Models with breed, treatment, and sex in the model.

Hormone /Transcript	Breed	Treatment	Sex	Breed x Treatment
Baseline CORT	ns	na	χ^2^ = 4.1 P < 0.05	ns
10 min CORT	χ^2^ = 5.2 P < 0.05	na	ns	ns
PREG	χ^2^ = 61.1 P < 0.001	na	ns	ns
DHEA	χ^2^ = 38.3 P < 0.001	na	ns	ns
*MC2R*	ns	ns	ns	ns
*STAR*	χ^2^ = 14 P < 0.01	ns	ns	ns
*POMC*	ns	χ^2^ = 31.9 P < 0.001	ns	ns
*CH25*	χ^2^ = 13.3 P < 0.01	χ^2^ = 14 P < 0.01	ns	ns
*FDX1*	ns	ns	ns	ns
*CYP17A1*	ns	ns	χ^2^ = 11.5 P < 0.01	ns
*CYP19A1*	ns	ns	χ^2^ = 11.7 P < 0.01	ns
*CYP21A2*	χ^2^ = 17.5 P < 0.001	ns	ns	ns
*HSD3B2*	ns	ns	ns	ns
*HSD11B1*	ns	ns	ns	ns

**χ**^**2**^and **bonferroni corrected P values** are given for breed, treatment, sex and breed x treatment interaction. PREG = pregnenolone, DHEA = dehydroepiandrosterone, CORT = corticosterone. Significant data (P < 0.05), ns = not significant.

## References

[b1] Price E. O. Behavioral development in animals undergoing domestication. Applied Animal Behaviour Science 65, 245–271 (1999).

[b2] Wilkins A. S., Wrangham R. W. & Fitch W. T. The “domestication syndrome” in mammals: a unified explanation based on neural crest cell behavior and genetics. Genetics 197, 795–808 (2014). 25024034 10.1534/genetics.114.165423 PMC4096361

[b3] Tixier-Boichard M., Bed’hom B. & Rognon X. Chicken domestication: From archeology to genomics. C R Biol 334, 197–204 (2011). 21377614 10.1016/j.crvi.2010.12.012

[b4] Campler M., Jöngren M. & Jensen P. Fearfulness in red junglefowl and domesticated White Leghorn chickens. Behav Processes 81, 39–43 (2009). 19154782 10.1016/j.beproc.2008.12.018

[b5] Schütz K. *et al.* Major Growth QTLs in Fowl Are Related to Fearful Behavior: Possible Genetic Links Between Fear Responses and Production Traits in a Red Junglefowl × White Leghorn Intercross. Behavior genetics 34, 121–130 (2004). 14739702 10.1023/B:BEGE.0000009481.98336.fc

[b6] Wright D. *et al.* The genetic architecture of domestication in the chicken: effects of pleiotropy and linkage. Molecular ecology 19, 5140–5156 (2010). 21040053 10.1111/j.1365-294X.2010.04882.x

[b7] Wright D. *et al.* Onset of Sexual Maturity in Female Chickens is Genetically Linked to Loci Associated with Fecundity and a Sexual Ornament. Reproduction in Domestic Animals 47, 31–36 (2012). 22212210 10.1111/j.1439-0531.2011.01963.x

[b8] Kerje S. *et al.* The twofold difference in adult size between the red junglefowl and White Leghorn chickens is largely explained by a limited number of QTLs. Anim Genet 34, 264–274 (2003). 12873214 10.1046/j.1365-2052.2003.01000.x

[b9] Ericsson M., Fallahsharoudi A., Bergquist J., Kushnir M. M. & Jensen P. Domestication effects on behavioural and hormonal responses to acute stress in chickens. Physiol Behav 133, 161–9 (2014). 24878317 10.1016/j.physbeh.2014.05.024

[b10] Payne A. H. & Hales D. B. Overview of steroidogenic enzymes in the pathway from cholesterol to active steroid hormones. Endocr. Rev. 25, 947–970 (2004). 15583024 10.1210/er.2003-0030

[b11] Xing Y., Parker C. R., Edwards M. & Rainey W. E. ACTH is a potent regulator of gene expression in human adrenal cells. J Mol Endocrinol 45, 59–68 (2010). 20460446 10.1677/JME-10-0006 PMC3725639

[b12] Mormede P. *et al.* Molecular genetics of hypothalamic–pituitary–adrenal axis activity and function. Ann NY Acad Sci 1220, 127–136 (2011). 21388410 10.1111/j.1749-6632.2010.05902.x

[b13] Treidman D. M. & Levine S. Plasma corticosteroid response to stress in four species of wild mice. Endocrinology 84, 676–680 (1969). 4304265 10.1210/endo-84-3-676

[b14] Albert F. W. *et al.* Phenotypic differences in behavior, physiology and neurochemistry between rats selected for tameness and for defensive aggression towards humans. Hormones and Behavior 53, 413–421 (2008). 18177873 10.1016/j.yhbeh.2007.11.010

[b15] Weiler U., Claus R., Schnoebelen-Combes S. & Louveau I. Influence of age and genotype on endocrine parameters and growth performance: a comparative study in Wild boars, Meishan and Large White boars. Livestock Production Science 54, 21–31 (1998).

[b16] Harri M., Mononen J., Ahola L., Plyusnina I. & Rekila T. Behavioural and physiological differences between silver foxes selected and not selected for domestic behaviour. Animal Welfare 12, 305–314 (2003).

[b17] Martin J. T. Embryonic Pituitary Adrenal Axis, Behavior Development and Domestication in Birds. Am Zool 18, 489–499 (1978).

[b18] Woodward C. C. & Strange R. J. Physiological stress responses in wild and hatchery-reared rainbow trout. Trans Am Fish Soc 116, 574–579 (1987).

[b19] Albert F. W. *et al.* A comparison of brain gene expression levels in domesticated and wild animals. PLoS Genet 8, e1002962 (2012). 23028369 10.1371/journal.pgen.1002962 PMC3459979

[b20] Andersson L. & Georges M. Domestic-animal genomics: deciphering the genetics of complex traits. Nature reviews. Genetics 5, 202–212 (2004). 10.1038/nrg1294 14970822

[b21] Saetre P. *et al.* From wild wolf to domestic dog: gene expression changes in the brain. Mol. Brain Res. 126, 198–206 (2004). 15249144 10.1016/j.molbrainres.2004.05.003

[b22] Jongren M., Westander J., Natt D. & Jensen P. Brain gene expression in relation to fearfulness in female red junglefowl (Gallus gallus). Genes, brain, and behavior 9, 751–758 (2010). 10.1111/j.1601-183X.2010.00612.x 20597989

[b23] Nätt D. *et al.* Inheritance of Acquired Behaviour Adaptations and Brain Gene Expression in Chickens. PLoS ONE 4, e6405 (2009). 19636381 10.1371/journal.pone.0006405 PMC2713434

[b24] Heyne H. O. *et al.* Genetic influences on brain gene expression in rats selected for tameness and aggression. Genetics 198, 1277–1290 (2014). 25189874 10.1534/genetics.114.168948 PMC4224166

[b25] Khaitovich P. *et al.* A neutral model of transcriptome evolution. PLoS Biol 2, e132 (2004). 15138501 10.1371/journal.pbio.0020132 PMC406393

[b26] Huang D. W., Sherman B. T. & Lempicki R. A. Systematic and integrative analysis of large gene lists using DAVID bioinformatics resources. Nature protocols 4, 44–57 (2008). 10.1038/nprot.2008.211 19131956

[b27] Sewer M. B. & Waterman M. R. ACTH modulation of transcription factors responsible for steroid hydroxylase gene expression in the adrenal cortex. Microsc Res Tech 61, 300–307 (2003). 12768545 10.1002/jemt.10339

[b28] Soleimani A. F., Zulkifli I., Omar A. R. & Raha A. R. Physiological responses of 3 chicken breeds to acute heat stress. Poult Sci 90, 1435–1440, (2011). 21673158 10.3382/ps.2011-01381

[b29] Trut L., Oskina I. & Kharlamova A. Animal evolution during domestication: the domesticated fox as a model. BioEssays : news and reviews in molecular, cellular and developmental biology 31, 349–360 (2009). 10.1002/bies.200800070 PMC2763232 19260016

[b30] Trut L. N. Early Canid Domestication: The Farm-Fox Experiment: Foxes bred for tamability in a 40-year experiment exhibit remarkable transformations that suggest an interplay between behavioral genetics and development. Am Sci 87, 160–169 (1999).

[b31] Belyaev D. K. Destabilizing selection as a factor in domestication. Journal of Heredity 70, 301–308 (1979). 528781 10.1093/oxfordjournals.jhered.a109263

[b32] Lennartsson A.-K., Kushnir M. M., Bergquist J. & Jonsdottir I. H. DHEA and DHEA-S response to acute psychosocial stress in healthy men and women. Biol. Psychol. 90, 143–149 (2012). 22445967 10.1016/j.biopsycho.2012.03.003

[b33] Schütz K. E., Forkman B. & Jensen P. Domestication effects on foraging strategy, social behaviour and different fear responses: a comparison between the red junglefowl (*Gallus gallus*) and a modern layer strain. Applied animal behaviour science 74, 1–14 (2001).

[b34] Stocco D. M. & Clark B. J. Regulation of the Acute Production of Steroids in Steroidogenic Cells. Endocr. Rev. 17, 221–244 (1996). 8771357 10.1210/edrv-17-3-221

[b35] Tomkins G. M., Garren L. D., Rodney Howell R. & Peterkofsky B. The regulation of enzyme synthesis by steroid hormones: The role of translation. Journal of Cellular and Comparative Physiology 66, 137–151 (1965). 10.1002/jcp.1030660413 4379390

[b36] Mormède P., Foury A., Terenina E. & Knap P. Breeding for robustness: the role of cortisol. Animal 5, 651–657 (2011). 22439987 10.1017/S1751731110002168

[b37] Desautes C., Sarrieau A., Caritez J. C. & Mormede P. Behavior and pituitary-adrenal function in large white and Meishan pigs. Domest. Anim. Endocrinol. 16, 193–205 (1999). 10370859 10.1016/s0739-7240(99)00014-4

[b38] Carsia R. V., Weber H. & Satterlee D. G. Steroidogenic properties of isolated adrenocortical cells from Japanese quail selected for high serum corticosterone response to immobilization. Domest. Anim. Endocrinol. 5, 231–240 (1988). 3224523 10.1016/0739-7240(88)90034-3

[b39] Rogers P. V. & Richter C. P. Anatomical comparison between the adrenal glands of wild norway, wild alexandrine and domestic norway rats 1. Endocrinology 42, 46–55 (1948). 18898933 10.1210/endo-42-1-46

[b40] Lee H.-H. Diversity of the CYP21P-like gene in CYP21 deficiency. DNA Cell Biol 24, 1–9 (2005). 15684714 10.1089/dna.2005.24.1

[b41] White P. C. & Speiser P. W. Congenital Adrenal Hyperplasia due to 21-Hydroxylase Deficiency 1. Endocr. Rev. 21, 245–291 (2000). 10857554 10.1210/edrv.21.3.0398

[b42] Scanes C. G. Sturkie’s Avian Physiology. (Elsevier, 2014).

[b43] Lacombe A. & Jones D. R. The source of circulating catecholamines in forced dived ducks. Gen Comp Endocrinol 80, 41–47 (1990). 2272478 10.1016/0016-6480(90)90146-d

[b44] Kato K., Nakagawa C., Murabayashi H. & Oomori Y. Expression and distribution of GABA and GABAB‐receptor in the rat adrenal gland. J Anat 224, 207–215 (2014). 24252118 10.1111/joa.12144 PMC3969063

[b45] Aramori I. & Nakanishi S. Signal transduction and pharmacological characteristics of a metabotropic glutamate receptor, mGluRl, in transfected CHO cells. Neuron 8, 757–765 (1992). 1314623 10.1016/0896-6273(92)90096-v

[b46] Chen K., Li H.-Z., Ye N., Zhang J. & Wang J.-J. Role of GABA B receptors in GABA and baclofen-induced inhibition of adult rat cerebellar interpositus nucleus neurons *in vitro*. Brain Res Bull 67, 310–318 (2005). 16182939 10.1016/j.brainresbull.2005.07.004

[b47] Flatmark. Catecholamine biosynthesis and physiological regulation in neuroendocrine cells. Acta Physiol Scand 168, 1–17 (2000). 10691773 10.1046/j.1365-201x.2000.00596.x

[b48] Haller J., Makara G. B. & Kruk M. R. Catecholaminergic involvement in the control of aggression: hormones, the peripheral sympathetic, and central noradrenergic systems. Neurosci. Biobehav. Rev. 22, 85–97 (1997). 9491941 10.1016/s0149-7634(97)00023-7

[b49] Cheng H. W. & Muir W. M. Mechanisms of aggression and production in chickens: genetic variations in the functions of serotonin, catecholamine, and corticosterone. World’s Poultry Science Journal 63, 233–254 (2007).

[b50] Rivier C. & Rivest S. Effect of stress on the activity of the hypothalamic-pituitary-gonadal axis: peripheral and central mechanisms. Biol Reprod 45, 523–532 (1991). 1661182 10.1095/biolreprod45.4.523

[b51] Stocco D. M. StAR protein and the regulation of steroid hormone biosynthesis. Annu Rev Physiol 63, 193–213 (2001). 11181954 10.1146/annurev.physiol.63.1.193

[b52] Lin D. *et al.* Role of steroidogenic acute regulatory protein in adrenal and gonadal steroidogenesis. Science 267, 1828–1831 (1995). 7892608 10.1126/science.7892608

[b53] Christenson L. K. & Strauss Iii J. F. Steroidogenic Acute Regulatory Protein: An Update on Its Regulation and Mechanism of Action. Arch. Med. Res. 32, 576–586 (2001). 11750733 10.1016/s0188-4409(01)00338-1

[b54] Lund E. G., Kerr T. A., Sakai J., Li W.-P. & Russell D. W. cDNA Cloning of Mouse and Human Cholesterol 25-Hydroxylases, Polytopic Membrane Proteins That Synthesize a Potent Oxysterol Regulator of Lipid Metabolism. J Biol Chem 273 (1998). 10.1074/jbc.273.51.34316 9852097

[b55] Reinhart A. J., Williams S. C. & Stocco D. M. Transcriptional regulation of the StAR gene. Mol Cell Endocrinol 151, 161–169 (1999). 10411331 10.1016/s0303-7207(98)00257-3

[b56] Siegel H. S. Physiological Stress in Birds. Bioscience 30, 529–534 (1980).

[b57] Lowry C. Functional subsets of serotonergic neurones: implications for control of the hypothalamic‐pituitary‐adrenal axis. J Neuroendocrinol 14, 911–923 (2002). 12421345 10.1046/j.1365-2826.2002.00861.x

[b58] Bornstein S. & Chrousos G. Adrenocorticotropin (ACTH)-and non-ACTH-mediated regulation of the adrenal cortex: neural and immune inputs. The Journal of Clinical Endocrinology & Metabolism 84, 1729–1736 (1999). 10323408 10.1210/jcem.84.5.5631

[b59] Karpac J., Kern A. & Hochgeschwender U. Pro-opiomelanocortin peptides and the adrenal gland. Mol Cell Endocrinol 265–266, 29–33 (2007). 10.1016/j.mce.2006.12.035 17222502

[b60] Harbuz M. & Lightman S. Responses of hypothalamic and pituitary mRNA to physical and psychological stress in the rat. J Endocrinol 122, 705–711 (1989). 2809478 10.1677/joe.0.1220705

[b61] Höllt V. *et al.* Stress-induced alterations in the levels of messenger RNA coding for proopiomelanocortin and prolactin in rat pituitary. Neuroendocrinology 43, 277–282 (1986). 2942792 10.1159/000124541

[b62] Matthews S. G. & Challis J. Regulation of CRH and AVP mRNA in the developing ovine hypothalamus: effects of stress and glucocorticoids. American Journal of Physiology-Endocrinology And Metabolism 268, E1096–E1107 (1995). 10.1152/ajpendo.1995.268.6.E1096 7611384

[b63] Gulevich R., Oskina I., Shikhevich S., Fedorova E. & Trut L. Effect of selection for behavior on pituitary–adrenal axis and proopiomelanocortin gene expression in silver foxes (Vulpes vulpes). Physiol Behav 82, 513–518 (2004). 15276817 10.1016/j.physbeh.2004.04.062

[b64] Mank J. E. Sex chromosome dosage compensation: definitely not for everyone. Trends Genet 29, 677–683 (2013). 23953923 10.1016/j.tig.2013.07.005

[b65] Nätt D., Agnvall B. & Jensen P. Large sex differences in chicken behavior and brain gene expression coincide with few differences in promoter DNA-methylation. PLoS ONE 9, e96376 (2014). 24782041 10.1371/journal.pone.0096376 PMC4004567

[b66] Ellegren H. & Parsch J. The evolution of sex-biased genes and sex-biased gene expression. Nat Rev Genet 8, 689–698 (2007). 17680007 10.1038/nrg2167

[b67] Schütz K. *et al.* QTL analysis of a red junglefowl ×White Leghorn intercross reveals trade-off in resource allocation between behavior and production traits. Behav Genet 32, 423–433 (2002). 12467340 10.1023/a:1020880211144

[b68] Liu Y. *et al.* Detection and confirmation of 60 anabolic and androgenic steroids in equine plasma by liquid chromatography‐tandem mass spectrometry with instant library searching. Drug testing and analysis 3, 54–67 (2011). 20878876 10.1002/dta.168

[b69] Rubin C. J. *et al.* Whole-genome resequencing reveals loci under selection during chicken domestication. Nature 464, 587–591 (2010). 20220755 10.1038/nature08832

[b70] Untergasser A. *et al.* Primer3—new capabilities and interfaces. Nucleic Acids Res 40, e115–e115 (2012). 22730293 10.1093/nar/gks596 PMC3424584

[b71] Koressaar T. & Remm M. Enhancements and modifications of primer design program Primer3. Bioinformatics 23, 1289–1291 (2007). 17379693 10.1093/bioinformatics/btm091

[b72] Ye J. *et al.* Primer-BLAST: a tool to design target-specific primers for polymerase chain reaction. BMC Bioinformatics 13, 134 (2012). 22708584 10.1186/1471-2105-13-134 PMC3412702

[b73] Pfaffl M. W. A new mathematical model for relative quantification in real-time RT–PCR. Nucleic Acids Res 29, e45–e45 (2001). 11328886 10.1093/nar/29.9.e45 PMC55695

[b74] Gautier L., Cope L., Bolstad B. M. & Irizarry R. A. affy—analysis of Affymetrix GeneChip data at the probe level. Bioinformatics 20, 307–315 (2004). 14960456 10.1093/bioinformatics/btg405

[b75] Hackstadt A. J. & Hess A. M. Filtering for increased power for microarray data analysis. BMC Bioinformatics 10, 11 (2009). 19133141 10.1186/1471-2105-10-11 PMC2661050

[b76] Smyth G. K. Linear models and empirical bayes methods for assessing differential expression in microarray experiments. Statistical applications in genetics and molecular biology 3 10.2202/1544-6115.1027 (2004). 16646809

[b77] Benjamini Y. & Hochberg Y. Controlling the false discovery rate: a practical and powerful approach to multiple testing. *Journal of the Royal Statistical Society*. Series B (Methodological) 57, 289–300 (1995).

[b78] Narum S. R. Beyond Bonferroni: less conservative analyses for conservation genetics. Conserv Genet 7, 783–787 (2006).

[b79] Huang D. W., Sherman B. T. & Lempicki R. A. Bioinformatics enrichment tools: paths toward the comprehensive functional analysis of large gene lists. Nucleic Acids Res 37, 1–13 (2009). 19033363 10.1093/nar/gkn923 PMC2615629

[b80] Rice W. R. Analyzing tables of statistical tests. Evolution 43, 223–225 (1989). 10.1111/j.1558-5646.1989.tb04220.x 28568501

