# Supplementary material for: Domestication Effects on Stress Induced Steroid Secretion and Adrenal Gene Expression in Chickens
Source: Sci Rep. 2015 Oct 16;5:15345. doi: 10.1038/srep15345 (PMC4608001; doi:10.1038/srep15345)
Supplement: Supplementary Information [file srep15345-s1.pdf]

Domestication Effects on Stress Induced Steroid Secretion and Adrenal Gene Expression in Chickens

Amir Fallahsharoudi, Neil de Kock, Martin Johnsson, S.J. Kumari A.Ubhayasekera, Jonas Bergquist, Dominic Wright, Per Jensen

***Supplementary Table S1** . The list of genes which are differently expressed between the breeds based on the ANOVA model (~breed + sex + treatment). The reported values include: transcription ID, gene, log2 fold change (logFC), P.Value, BH adjusted P.Value (adj.P.Val), and B Value.   The positive logFC values suggest that the gene in up regulated in red Junglefowl and negative logFC values suggest up-regulation of the gene in White Leghorn.*

Transcript ID	Gene	logFC	t	P.Value	adj.P.Val	B
ENSGALT00000031128		3,700917	17,03646	1,43E-13	7,28E-10	20,86891
ENSGALT00000026116	CRYAA	3,118613	16,25548	3,51E-13	8,94E-10	20,05359
NM_001114503	LOC420716	3,105127	14,13645	4,9E-12	5E-09	17,61076
ENSGALT00000006932	OIT3	1,531119	13,32446	1,47E-11	1,25E-08	16,5746
ENSGALT00000038412	LOC771622	-1,67813	-11,9985	9,98E-11	7,27E-08	14,74644
ENSGALT00000027130	Q5ZL81	1,945179	11,59135	1,86E-10	9,46E-08	14,14844
ENSGALT00000037332	Q4F8N1	1,125395	11,35853	2,67E-10	1,24E-07	13,79825
ENSGALT00000008508	CYP8B1	1,913597	11,26765	3,08E-10	1,31E-07	13,6599
ENSGALT00000017666	TACSTD2	1,517356	10,52763	1,02E-09	3,39E-07	12,49753
ENSGALT00000011338	HTR3A	-1,92363	-10,5096	1,05E-09	3,39E-07	12,46846
ENSGALT00000006859	SYT12	1,319304	10,50146	1,06E-09	3,39E-07	12,45522
ENSGALT00000023272	Q4F8N1	1,127171	10,42638	1,2E-09	3,61E-07	12,3334
ENSGALT00000037523		-1,6143	-10,3586	1,35E-09	3,82E-07	12,22282
NM_001031138	SPATA4	-1,52623	-10,2387	1,65E-09	4,43E-07	12,02584
ENSGALT00000011807	VWA5B2	-1,15995	-9,84211	3,25E-09	7,9E-07	11,36135
ENSGALT00000040840	LOC769903	-1,38409	-9,77951	3,63E-09	8,4E-07	11,25464
ENSGALT00000006151	P2RX7	0,903473	9,686885	4,26E-09	9,45E-07	11,09581
ENSGALT00000021599	CLEC3A	1,592659	9,591737	5,04E-09	1,03E-06	10,93149
ENSGALT00000006646	GAA	1,220094	9,441864	6,57E-09	1,26E-06	10,67026
ENSGALT00000027322	GPC5	-0,72853	-9,43447	6,66E-09	1,26E-06	10,6573
ENSGALT00000025794	KCNE2	1,339481	9,399271	7,09E-09	1,27E-06	10,59548
ENSGALT00000037403	LOC426177	1,498349	9,3674	7,51E-09	1,28E-06	10,53936
NM_205507	CKM	-4,21068	-9,31831	8,2E-09	1,35E-06	10,45267
NM_204555	KCNIP4	1,086124	9,300699	8,47E-09	1,35E-06	10,42149
ENSGALT00000017553	SLC24A4	-1,39242	-9,24423	9,37E-09	1,45E-06	10,32122
NM_205240	ST6GALNAC1	1,263788	9,190091	1,03E-08	1,55E-06	10,22471
ENSGALT00000014060	LOC771876	1,067757	9,043783	1,35E-08	1,91E-06	9,961894
NM_001006424	SLC9A3R1	1,014137	8,99037	1,49E-08	2,05E-06	9,865225
ENSGALT00000022889	Q5ZL81	0,873491	8,948974	1,61E-08	2,16E-06	9,790038
ENSGALT00000025405	LOC431640	-1,55153	-8,92286	1,69E-08	2,2E-06	9,742483

ENSGALT00000011312	EEF2K	-1,31831	-8,86356	1,88E-08	2,4E-06	9,634169
NM_001005571	CYP8B1	1,386052	8,84586	1,94E-08	2,42E-06	9,601738
ENSGALT00000027932	LEG6	1,146905	8,787484	2,17E-08	2,63E-06	9,494492
ENSGALT00000039640	CRBA1	1,029894	8,733454	2,4E-08	2,84E-06	9,394811
ENSGALT00000015148	CNIH3	1,212002	8,703423	2,54E-08	2,94E-06	9,339232
NM_204407	GRPR	1,139368	8,668987	2,71E-08	2,97E-06	9,275348
ENSGALT00000023402	Q5ZLD8	-1,18226	-8,66253	2,74E-08	2,97E-06	9,263346
NM_001024580	VIT	1,711972	8,592308	3,13E-08	3,25E-06	9,13251
ENSGALT00000020631	LOC420807	1,158813	8,580192	3,2E-08	3,26E-06	9,109865
ENSGALT00000034819	LOC772031	-0,94724	-8,55732	3,34E-08	3,34E-06	9,067071
ENSGALT00000007846	Q5ZHY2	-0,98871	-8,53512	3,48E-08	3,42E-06	9,025444
NM_001030709	NCF1	1,112783	8,453917	4,07E-08	3,91E-06	8,87265
ENSGALT00000008506	CCBP2	1,281234	8,440028	4,18E-08	3,94E-06	8,846422
ENSGALT00000022348	C6orf94	-1,1271	-8,41612	4,37E-08	4,05E-06	8,80121
ENSGALT00000024915	ZNF462	-0,93225	-8,40255	4,49E-08	4,05E-06	8,775515
ENSGALT00000006743	FTSJ2	-0,95389	-8,39771	4,53E-08	4,05E-06	8,766351
ENSGALT00000025402	ATP5J	1,077822	8,344538	5,01E-08	4,41E-06	8,665379
ENSGALT00000039332	TEX14	-1,10038	-8,30931	5,37E-08	4,64E-06	8,59827
ENSGALT00000010717	C7orf23	1,326041	8,187677	6,79E-08	5,77E-06	8,36521
ENSGALT00000037526	C6orf94	-1,09437	-8,14384	7,39E-08	6,18E-06	8,28071
ENSGALT00000006413	MATN4	1,127717	8,104724	7,98E-08	6,56E-06	8,205077
ENSGALT00000030398	PIT1	0,889692	8,082532	8,33E-08	6,7E-06	8,162073
ENSGALT00000020664	KIAA0319	-0,95027	-8,07692	8,42E-08	6,7E-06	8,15119
ENSGALT00000019750	FAM62C	0,805884	8,014351	9,52E-08	7,32E-06	8,029517
ENSGALT00000018096	MAPK10	-0,77205	-8,0017	9,76E-08	7,32E-06	8,004849
ENSGALT00000042268	SNORA5	-0,76455	-7,9829	1,01E-07	7,49E-06	7,968157
NM_001006292	SLC17A9	0,969788	7,972842	1,03E-07	7,53E-06	7,948496
ENSGALT00000018056	KCNH7	-1,10124	-7,93442	1,11E-07	8,01E-06	7,87329
ENSGALT00000025631	SLC7A7	-1,38228	-7,90442	1,18E-07	8,38E-06	7,814415
ENSGALT00000024646	OPRK1	-2,93827	-7,8844	1,23E-07	8,6E-06	7,775059
ENSGALT00000013741	SORCS3	-1,35435	-7,86418	1,28E-07	8,83E-06	7,735256
ENSGALT00000007554	EPHA8	-1,13066	-7,85561	1,3E-07	8,86E-06	7,718359
ENSGALT00000011207	GDE1	-0,94124	-7,8463	1,33E-07	8,91E-06	7,699997
ENSGALT00000036663	CRLD1	0,721024	7,826577	1,38E-07	8,97E-06	7,661069
ENSGALT00000017631	LOC423425	-1,05505	-7,82328	1,39E-07	8,97E-06	7,654552
ENSGALT00000026387	PRDX4	-2,11323	-7,80549	1,44E-07	9,01E-06	7,619372
NM_205502	CRYBA1	0,932161	7,802122	1,45E-07	9,01E-06	7,612716
ENSGALT00000041065	PTPRU	-1,0245	-7,78521	1,5E-07	9,16E-06	7,579233
ENSGALT00000011818	PYCR2	0,85392	7,779397	1,52E-07	9,16E-06	7,56771
ENSGALT00000036758	CENPO	1,003832	7,775943	1,53E-07	9,16E-06	7,560864
ENSGALT00000009605	ZNF488	-0,77737	-7,75597	1,59E-07	9,34E-06	7,521229
ENSGALT00000016175	HHIP	1,289467	7,754878	1,59E-07	9,34E-06	7,519068
ENSGALT00000025406	TMEM215	1,435441	7,749119	1,61E-07	9,34E-06	7,507633
ENSGALT00000028520	snoZ40	-1,56308	-7,7054	1,76E-07	1E-05	7,420664
ENSGALT00000020949	LOC418109	-1,1536	-7,70047	1,78E-07	1E-05	7,410839
ENSGALT00000011065	LOC416959	0,911002	7,696652	1,79E-07	1E-05	7,40322
ENSGALT00000015259	Q9PVI5	-0,69514	-7,69186	1,81E-07	1E-05	7,393656
ENSGALT00000028083	LOC420510	0,799	7,638402	2,01E-07	1,1E-05	7,286845
NM_001039288	VNN1	0,838571	7,592376	2,21E-07	1,2E-05	7,194554
ENSGALT00000015459	NAF1	0,743394	7,573143	2,3E-07	1,23E-05	7,1559

NM_204685	F13A1	1,226782	7,541419	2,45E-07	1,3E-05	7,092028
ENSGALT00000009403	SRMS	-1,2951	-7,53164	2,5E-07	1,3E-05	7,072308
NM_001012768	MYEF2	-0,97901	-7,51855	2,57E-07	1,3E-05	7,045895
NM_001031099	CENPO	1,009496	7,512696	2,6E-07	1,3E-05	7,034077
ENSGALT00000012751	PAPOLG	1,460224	7,511235	2,6E-07	1,3E-05	7,031126
ENSGALT00000006436	CSRP3	0,832422	7,505486	2,63E-07	1,3E-05	7,019512
ENSGALT00000028571	SNORA3	-0,80822	-7,50387	2,64E-07	1,3E-05	7,016238
ENSGALT00000017447	GLIS1	1,608554	7,501262	2,66E-07	1,3E-05	7,010975
ENSGALT00000034185	PPP1R3D	-1,19638	-7,49484	2,69E-07	1,3E-05	6,997988
ENSGALT00000015561	DCAF17	-0,83171	-7,49126	2,71E-07	1,3E-05	6,990756
ENSGALT00000011985	SCN4B	0,763784	7,482679	2,76E-07	1,31E-05	6,973389
ENSGALT00000020677	DCDC2	-1,41564	-7,46086	2,89E-07	1,34E-05	6,929201
ENSGALT00000017224	VIT	1,167255	7,460645	2,89E-07	1,34E-05	6,928761
ENSGALT00000029893	LMOD3	0,9225	7,416536	3,16E-07	1,43E-05	6,839219
ENSGALT00000009581	TRIM66	1,215815	7,415981	3,16E-07	1,43E-05	6,838091
ENSGALT00000042098	SNORA35	-0,82817	-7,41483	3,17E-07	1,43E-05	6,835748
ENSGALT00000012341	LOC768955	-0,81387	-7,37314	3,45E-07	1,53E-05	6,750853
ENSGALT00000007234	RXFP2	1,037889	7,369444	3,48E-07	1,53E-05	6,74332
ENSGALT00000025248	CRLD1	0,762133	7,355122	3,58E-07	1,54E-05	6,714092
ENSGALT00000026877	Q3L248	1,573508	7,352204	3,6E-07	1,54E-05	6,708134
NM_205233	ST6GALNAC2	0,71729	7,339896	3,7E-07	1,56E-05	6,682988
ENSGALT00000038406	EML4	-0,96264	-7,33785	3,71E-07	1,56E-05	6,678805
NM_001024584	HCRTR2	-1,22499	-7,32356	3,82E-07	1,57E-05	6,649591
NM_204319	POU1F1	1,075339	7,288169	4,11E-07	1,67E-05	6,577079
ENSGALT00000032903	LOC771515	1,004233	7,278779	4,19E-07	1,67E-05	6,557814
ENSGALT00000007272	UNC5A	-0,75472	-7,27759	4,2E-07	1,67E-05	6,555363
ENSGALT00000012627	DOK5	-0,85082	-7,27439	4,23E-07	1,67E-05	6,548805
NM_001172781	HTR1B	-1,23144	-7,27391	4,24E-07	1,67E-05	6,547809
ENSGALT00000034450	LOC419182	-1,04697	-7,26672	4,3E-07	1,68E-05	6,533047
NM_001040464	GHRH	-0,80279	-7,26592	4,31E-07	1,68E-05	6,531416
ENSGALT00000023358	RGS8	1,325372	7,26036	4,36E-07	1,68E-05	6,519987
ENSGALT00000012349	MEGF11	0,773485	7,256914	4,39E-07	1,68E-05	6,512905
ENSGALT00000018621	LOC424729	-1,26812	-7,25303	4,42E-07	1,68E-05	6,50491
ENSGALT00000019860	KIF18A	0,759997	7,227506	4,66E-07	1,76E-05	6,452399
ENSGALT00000021239	ROPN1L	1,264323	7,217677	4,76E-07	1,76E-05	6,432148
ENSGALT00000018097	MAPK10	-0,70835	-7,21607	4,77E-07	1,76E-05	6,428829
ENSGALT00000019757	SLC35F4	-0,78612	-7,20889	4,84E-07	1,77E-05	6,414035
ENSGALT00000010538	HCK	-1,40251	-7,20646	4,87E-07	1,77E-05	6,409029
ENSGALT00000031682	Q9PSA3	1,064636	7,177231	5,17E-07	1,86E-05	6,34868
ENSGALT00000033031	LOC425004	1,119506	7,176262	5,18E-07	1,86E-05	6,346677
ENSGALT00000010857	RTDR1	0,934795	7,172014	5,23E-07	1,86E-05	6,337898
ENSGALT00000025443	Q9PSA3	1,136548	7,170497	5,25E-07	1,86E-05	6,334762
ENSGALT00000038490	TNPO1	-0,82681	-7,1313	5,69E-07	1,99E-05	6,253607
ENSGALT00000024555	Q5ZJ91	0,767797	7,101535	6,06E-07	2,1E-05	6,191858
ENSGALT00000006313	LOC770143	0,826697	7,088117	6,23E-07	2,15E-05	6,163977
ENSGALT00000014014	Q90707	-0,61994	-7,07419	6,41E-07	2,19E-05	6,135021
ENSGALT00000027355	LMO7	-0,78311	-7,06254	6,57E-07	2,23E-05	6,110754
ENSGALT00000030823	ODF1	-0,80461	-7,04397	6,83E-07	2,31E-05	6,072059
ENSGALT00000030259	GABRG3	-1,49238	-7,00756	7,38E-07	2,47E-05	5,996074
ENSGALT00000016703	KCAB1	0,997403	6,99624	7,55E-07	2,52E-05	5,972401

ENSGALT00000016403	GARNL1	-0,84201	-6,97657	7,87E-07	2,61E-05	5,931244
ENSGALT00000038352	LOC420510	0,739963	6,952756	8,28E-07	2,65E-05	5,881327
ENSGALT00000023625	C1R	0,833091	6,948472	8,35E-07	2,65E-05	5,87234
ENSGALT00000033467	FAM126B	-0,80156	-6,94766	8,37E-07	2,65E-05	5,87064
ENSGALT00000011813	MAFG	-0,94891	-6,94576	8,4E-07	2,65E-05	5,866645
ENSGALT00000039053	KCAB1	1,387893	6,943669	8,44E-07	2,65E-05	5,862262
ENSGALT00000037334	DCDC2	-1,39218	-6,94193	8,47E-07	2,65E-05	5,858615
ENSGALT00000038008	LOC417978	0,758423	6,933198	8,63E-07	2,68E-05	5,840279
ENSGALT00000006114	TRPM1	-0,92152	-6,93117	8,66E-07	2,68E-05	5,83601
ENSGALT00000031277	WDR86	0,832935	6,928508	8,71E-07	2,68E-05	5,830429
ENSGALT00000014282	SPATA1	-0,79616	-6,91371	8,99E-07	2,74E-05	5,799328
ENSGALT00000026539	KCNF1	1,129957	6,904643	9,16E-07	2,75E-05	5,780257
ENSGALT00000010442	XKR7	-0,65235	-6,90347	9,18E-07	2,75E-05	5,777781
NM_204856	NEGR1	-0,6562	-6,90274	9,2E-07	2,75E-05	5,776243
ENSGALT00000018798	Q90707	-0,6379	-6,90103	9,23E-07	2,75E-05	5,772664
ENSGALT00000042575	SNORA40	-0,85111	-6,89306	9,39E-07	2,77E-05	5,755888
ENSGALT00000027834	SNORA32	-0,81477	-6,89204	9,41E-07	2,77E-05	5,753722
ENSGALT00000041062	PTPRU	-0,81801	-6,88035	9,64E-07	2,83E-05	5,729116
ENSGALT00000024919	CA8	-1,03826	-6,8757	9,74E-07	2,84E-05	5,719304
ENSGALT00000040006	ZNF488	-0,81362	-6,8572	1,01E-06	2,93E-05	5,680297
ENSGALT00000014239	WDR85	0,738923	6,850023	1,03E-06	2,96E-05	5,665137
ENSGALT00000019345	KCNH5	-1,11517	-6,8457	1,04E-06	2,97E-05	5,656005
ENSGALT00000040310	PAPOLG	1,332095	6,836372	1,06E-06	3,02E-05	5,636303
ENSGALT00000042552	SNORA31	-0,6624	-6,82826	1,08E-06	3,05E-05	5,619167
NM_001004405	CAPN3	0,828289	6,808099	1,12E-06	3,15E-05	5,576503
ENSGALT00000014668	C20orf94	0,60024	6,789788	1,17E-06	3,24E-05	5,537717
NM_204267	LITAF	0,853254	6,774042	1,21E-06	3,33E-05	5,504328
ENSGALT00000027810	SESN3	-1,02272	-6,75077	1,27E-06	3,48E-05	5,454911
ENSGALT00000011835	LOC428249	-0,7213	-6,74717	1,28E-06	3,49E-05	5,447263
ENSGALT00000030257	GABRG3	-1,67588	-6,74192	1,29E-06	3,51E-05	5,436113
ENSGALT00000017981	LOC424180	-0,95366	-6,72709	1,34E-06	3,6E-05	5,404563
NM_001031430	SYK	0,861104	6,720986	1,35E-06	3,63E-05	5,391577
ENSGALT00000008831	LOC424033	1,92867	6,674184	1,5E-06	3,97E-05	5,291808
ENSGALT00000014708	GANC	-0,67636	-6,66933	1,51E-06	3,97E-05	5,281449
ENSGALT00000042361	gga-mir-1463	-0,76908	-6,6693	1,51E-06	3,97E-05	5,281368
NM_001030861	LOC419289	-1,32034	-6,66059	1,54E-06	4,03E-05	5,262782
ENSGALT00000010623	Q56IA1	1,27825	6,646006	1,59E-06	4,12E-05	5,231599
ENSGALT00000015214	MAP9	0,800766	6,644844	1,59E-06	4,12E-05	5,229112
ENSGALT00000032807	Q5ZJ91	0,697713	6,638246	1,62E-06	4,16E-05	5,214998
ENSGALT00000021965	MTDC	0,794299	6,607828	1,73E-06	4,42E-05	5,149856
ENSGALT00000026786	TRIM35	0,537045	6,605037	1,74E-06	4,43E-05	5,143873
ENSGALT00000007120	ASTN1	-0,77015	-6,58141	1,83E-06	4,61E-05	5,093184
NM_001130387	IFNGR1	-1,56995	-6,57717	1,84E-06	4,63E-05	5,084069
NM_001014970	VIPR2	1,254264	6,565318	1,89E-06	4,73E-05	5,058612
ENSGALT00000026792	ANGPTL7	0,843785	6,559658	1,91E-06	4,76E-05	5,046447
ENSGALT00000024921	MAEL	1,500229	6,545748	1,97E-06	4,88E-05	5,01653
ENSGALT00000014659	ZNF385B	1,457696	6,541681	1,99E-06	4,9E-05	5,007778
NM_204492	PTPRU	-0,78301	-6,52678	2,06E-06	5,04E-05	4,975685
ENSGALT00000004309	CCDC57	-0,54557	-6,48702	2,24E-06	5,47E-05	4,88993
ENSGALT00000024304	C18orf17	-0,7963	-6,47619	2,29E-06	5,57E-05	4,866552

ENSGALT00000030506	CDKN1A	-0,67234	-6,46539	2,35E-06	5,65E-05	4,843202
ENSGALT00000004382	FA2H	0,683445	6,465098	2,35E-06	5,65E-05	4,842573
ENSGALT00000025989	LOC428249	-0,72235	-6,46202	2,37E-06	5,66E-05	4,835914
ENSGALT00000020616	SLC35D2	-2,21311	-6,45036	2,43E-06	5,76E-05	4,810699
ENSGALT00000026261	IL1RAPL1	-0,70931	-6,44958	2,43E-06	5,76E-05	4,809011
ENSGALT00000024288	DDO	0,742206	6,446906	2,44E-06	5,77E-05	4,803218
ENSGALT00000015063	ENO4	-0,65426	-6,4451	2,45E-06	5,77E-05	4,799309
ENSGALT00000008014	SMCR7	0,584395	6,426277	2,56E-06	5,98E-05	4,758541
NM_001111130	CCNRC01	-0,74335	-6,41984	2,59E-06	6,01E-05	4,744598
ENSGALT00000012442	FAM19A4	-0,97281	-6,41568	2,62E-06	6,01E-05	4,735568
ENSGALT00000026217	SYTL5	-1,54068	-6,40807	2,66E-06	6,07E-05	4,719069
NM_001012843	MOV10	0,804473	6,403693	2,69E-06	6,07E-05	4,709567
ENSGALT00000025648	CALB1	-1,20709	-6,40268	2,69E-06	6,07E-05	4,707379
ENSGALT00000028519	SNORA1	-0,80214	-6,39123	2,76E-06	6,2E-05	4,682506
ENSGALT00000013373	FRMD5	-1,0395	-6,34168	3,08E-06	6,82E-05	4,574757
ENSGALT00000008108	TMEM103	0,847869	6,338488	3,1E-06	6,82E-05	4,567808
ENSGALT00000032455	LOC429947	-1,35349	-6,3379	3,1E-06	6,82E-05	4,566522
ENSGALT00000032587	C14orf126	-0,68879	-6,33356	3,13E-06	6,82E-05	4,557071
ENSGALT00000016469	GLO1	0,786641	6,328682	3,16E-06	6,86E-05	4,546442
NM_001030600	SLC6A6	-1,09704	-6,32609	3,18E-06	6,87E-05	4,540799
ENSGALT00000008609	CHDH	0,909974	6,321678	3,21E-06	6,91E-05	4,531175
ENSGALT00000037651	NFYB	-0,63059	-6,30194	3,36E-06	7,16E-05	4,488127
ENSGALT00000010700	KY	0,738573	6,286155	3,47E-06	7,38E-05	4,453647
NM_204544	FET1	-0,74827	-6,26933	3,6E-06	7,57E-05	4,416879
NM_204609	MX1	1,600096	6,266577	3,63E-06	7,58E-05	4,410853
ENSGALT00000024379	APBA1	-0,75709	-6,24893	3,77E-06	7,84E-05	4,372247
ENSGALT00000037300	Q8QGD8	1,639944	6,244217	3,81E-06	7,88E-05	4,361917
ENSGALT00000007475	NLE1	0,54945	6,239723	3,85E-06	7,91E-05	4,352076
ENSGALT00000014513	PEX5L	-0,75951	-6,23068	3,92E-06	8E-05	4,332258
NM_205201	LOC396120	1,000279	6,228384	3,94E-06	8,01E-05	4,32723
ENSGALT00000005498	VWC2L	0,772139	6,224049	3,98E-06	8,05E-05	4,317729
NM_001030928	KIF18B	0,566579	6,222552	3,99E-06	8,05E-05	4,314447
ENSGALT00000010371	Q5F431	-1,12386	-6,22041	4,01E-06	8,06E-05	4,30975
ENSGALT00000017582	RYR2	-0,73126	-6,21413	4,07E-06	8,14E-05	4,295984
NM_204167	HPSE	0,987627	6,20056	4,19E-06	8,35E-05	4,2662
NM_001030663	HVCN1	1,080918	6,184422	4,35E-06	8,59E-05	4,230758
NM_204796	NEUROG2	-0,93286	-6,16894	4,5E-06	8,85E-05	4,196733
ENSGALT00000039397	GIMAP8	1,285535	6,167314	4,51E-06	8,85E-05	4,193153
ENSGALT00000026103	KCNK9	-0,95327	-6,16382	4,55E-06	8,88E-05	4,185468
ENSGALT00000010221	CLCA2	0,837422	6,159709	4,59E-06	8,93E-05	4,176425
ENSGALT00000040168	RHOC	0,584313	6,157751	4,61E-06	8,93E-05	4,172117
ENSGALT00000042567	SNORD62	-1,13125	-6,14229	4,77E-06	9,11E-05	4,13809
ENSGALT00000024978	C6orf168	-1,62966	-6,14187	4,77E-06	9,11E-05	4,137167
NM_204660	CACNG3	-0,66958	-6,13594	4,84E-06	9,19E-05	4,124092
ENSGALT00000034903	PTGS1	0,955647	6,134503	4,85E-06	9,19E-05	4,12093
ENSGALT00000018418	NKIRAS1	0,642718	6,132363	4,87E-06	9,19E-05	4,116215
NM_001177575	PTH1R	-0,8272	-6,11914	5,02E-06	9,37E-05	4,087077
ENSGALT00000018074	PDE4B	-0,7744	-6,1141	5,08E-06	9,44E-05	4,07595
ENSGALT00000042579	snoU90	-0,89359	-6,10985	5,12E-06	9,5E-05	4,066575
ENSGALT00000013616	THBD	-0,9073	-6,09294	5,32E-06	9,83E-05	4,029261

ENSGALT00000040403	Q9YI45	-1,03801	-6,08986	5,36E-06	9,85E-05	4,022455
ENSGALT00000005262	OSGIN1	0,811115	6,087439	5,39E-06	9,85E-05	4,017112
ENSGALT00000017075	C10orf39	-0,71015	-6,08689	5,39E-06	9,85E-05	4,015907
ENSGALT00000031517	KIFC1	1,488117	6,074794	5,54E-06	0,000101	3,989173
NM_204315	ITGB3	0,926896	6,067308	5,63E-06	0,000102	3,972624
ENSGALT00000019793	Q9I8D5	0,805471	6,064555	5,67E-06	0,000102	3,966538
ENSGALT00000011840	CCDC64	-0,63461	-6,058	5,75E-06	0,000103	3,952031
ENSGALT00000037299	Q8QGD8	1,092332	6,057806	5,75E-06	0,000103	3,95161
ENSGALT00000036888	TMEM200C	1,159894	6,049741	5,86E-06	0,000105	3,933765
ENSGALT00000037222	LOC777026	-0,91255	-6,03633	6,03E-06	0,000108	3,904073
ENSGALT00000042099	gga-mir-1572	-1,09647	-6,02878	6,14E-06	0,000109	3,887358
NM_204316	ADORA1	0,851555	6,02456	6,19E-06	0,00011	3,878
ENSGALT00000026619	TP53I3	0,96994	6,022703	6,22E-06	0,00011	3,873885
ENSGALT00000006426	CRBA1	0,787797	6,017973	6,28E-06	0,00011	3,863401
ENSGALT00000030063	Q6V0P4	-1,471	-6,01762	6,29E-06	0,00011	3,862618
ENSGALT00000024157		1,202741	6,007076	6,44E-06	0,000112	3,839237
NM_001031232	TMEM180	0,874209	6,00648	6,45E-06	0,000112	3,837916
ENSGALT00000031321	LOC420043	0,77706	6,004417	6,48E-06	0,000112	3,833338
ENSGALT00000004874	TMEM100	0,68291	6,001507	6,52E-06	0,000112	3,826884
ENSGALT00000042612	U1	-0,8205	-5,99417	6,63E-06	0,000113	3,810593
ENSGALT00000025939	EXOC6B	-0,88208	-5,99233	6,65E-06	0,000113	3,806511
ENSGALT00000019784	TMEM37	0,820083	5,99151	6,67E-06	0,000113	3,804696
ENSGALT00000031464	KCNH2	-0,67102	-5,99	6,69E-06	0,000113	3,801342
ENSGALT00000041121	Q9PS75	-0,64716	-5,98071	6,83E-06	0,000115	3,780723
ENSGALT00000018774	Q5ZLZ0	-1,09445	-5,95339	7,26E-06	0,000122	3,720003
ENSGALT00000023749	RAB3C	-0,47779	-5,93505	7,56E-06	0,000127	3,679181
NM_204906	KCNAB1	1,284539	5,926945	7,7E-06	0,000129	3,661137
ENSGALT00000023052	LOC777026	-0,6892	-5,9223	7,78E-06	0,000129	3,650787
ENSGALT00000017868	SERPINA10	1,094241	5,921947	7,79E-06	0,000129	3,650006
NM_001031360	MTHFD2	0,730828	5,920496	7,81E-06	0,000129	3,646772
ENSGALT00000038623	Q5Y1E8	-0,77382	-5,9163	7,89E-06	0,00013	3,63742
ENSGALT00000034299	LOC769251	-0,7994	-5,88725	8,42E-06	0,000138	3,572648
ENSGALT00000042214	gga-mir-1467	-1,33079	-5,8831	8,5E-06	0,000139	3,56339
ENSGALT00000027616	LNX2	-0,72306	-5,87582	8,64E-06	0,00014	3,547128
ENSGALT00000013206	EML6	-0,51598	-5,87574	8,64E-06	0,00014	3,546948
ENSGALT00000012457	BCL9L	-0,85316	-5,87497	8,65E-06	0,00014	3,545236
ENSGALT00000038969	IF4A3	-0,69629	-5,87412	8,67E-06	0,00014	3,54334
NM_001007827	DD1	-0,76003	-5,87021	8,75E-06	0,000141	3,534601
ENSGALT00000018784	FAM198A	0,975079	5,857095	9,01E-06	0,000144	3,505301
ENSGALT00000041927	SNORD61	-1,09029	-5,84481	9,26E-06	0,000148	3,47784
ENSGALT00000019159	TMEM140	0,631619	5,843661	9,28E-06	0,000148	3,475269
ENSGALT00000039214	CCDC13	0,859519	5,842492	9,31E-06	0,000148	3,472653
NM_204347	LIX1	0,722504	5,835534	9,45E-06	0,00015	3,457091
NM_001081698	KIFC1	1,006315	5,832371	9,52E-06	0,00015	3,450013
ENSGALT00000027858	FOLH1	-0,81056	-5,82592	9,66E-06	0,000152	3,435569
NM_204358	BMP2	0,631693	5,824401	9,69E-06	0,000152	3,432177
ENSGALT00000026078	SLC37A1	-0,91429	-5,79102	1,05E-05	0,000162	3,357405
ENSGALT00000025967	SLC30A8	-0,84633	-5,7909	1,05E-05	0,000162	3,35713
ENSGALT00000036895	MYSC	-0,93499	-5,79027	1,05E-05	0,000162	3,35572
ENSGALT00000015001	LOC770184	0,794658	5,790028	1,05E-05	0,000162	3,355172

ENSGALT00000036405	Q6V0P4	-0,71861	-5,78226	1,07E-05	0,000164	3,337745
ENSGALT00000022862	A4GALT	-0,75987	-5,77745	1,08E-05	0,000165	3,326952
ENSGALT00000012870	CNTN6	-1,07394	-5,77671	1,08E-05	0,000165	3,325314
ENSGALT00000017834	PPFIA2	-0,53983	-5,76984	1,1E-05	0,000167	3,309892
ENSGALT00000022968	GABRA4	1,019818	5,765206	1,11E-05	0,000169	3,299487
ENSGALT00000012664	VASN	0,575312	5,759569	1,12E-05	0,000169	3,286831
NM_001030682	SURF6	0,818946	5,753262	1,14E-05	0,000171	3,272669
ENSGALT00000023816	HAO2	-1,53935	-5,75126	1,14E-05	0,000171	3,268179
NM_001006264	PRPS2	-1,0732	-5,74443	1,16E-05	0,000174	3,252838
ENSGALT00000023629	C1R	0,73984	5,740371	1,17E-05	0,000175	3,243706
ENSGALT00000025241	PALM2	-0,65751	-5,73817	1,18E-05	0,000175	3,238755
ENSGALT00000007167	SLC2A10	0,770788	5,728342	1,2E-05	0,000178	3,216667
ENSGALT00000007042	SDK1	-0,97112	-5,72559	1,21E-05	0,000178	3,210468
ENSGALT00000030449	MOR1A	0,750516	5,718138	1,23E-05	0,000181	3,193717
ENSGALT00000036248		-0,70452	-5,71768	1,23E-05	0,000181	3,192681
ENSGALT00000019404	B2CL09	-0,81382	-5,69393	1,3E-05	0,00019	3,139222
ENSGALT00000025530	GRIK1	-0,68801	-5,68957	1,31E-05	0,000191	3,129418
NM_204621	PTPRJchr818221	-1,42721	-5,68559	1,33E-05	0,000192	3,120447
ENSGALT00000037273		1,278335	5,682164	1,34E-05	0,000192	3,112724
ENSGALT00000035334	gga-mir-216c	1,045991	5,680037	1,34E-05	0,000193	3,10793
NM_001115079	ASIP	-1,44641	-5,675	1,36E-05	0,000195	3,096589
NM_001127170	GPR103	-0,91513	-5,66924	1,38E-05	0,000197	3,083602
ENSGALT00000031776	IFNGR1	-0,76129	-5,66651	1,38E-05	0,000197	3,077427
ENSGALT00000037301	Q5ZLD8	-0,8264	-5,65817	1,41E-05	0,0002	3,058619
ENSGALT00000005789	RGS16	-1,14215	-5,65707	1,41E-05	0,0002	3,056143
ENSGALT00000037181	Q9PSA4	0,756151	5,653185	1,43E-05	0,000201	3,047382
NM_214673	CDH10	-0,60312	-5,6524	1,43E-05	0,000201	3,045622
ENSGALT00000011699	DYSFIP1	-0,77219	-5,64643	1,45E-05	0,000202	3,032136
ENSGALT00000022325	TMEM181	-1,08118	-5,64155	1,47E-05	0,000204	3,021136
ENSGALT00000018380	KCNH8	1,314125	5,63606	1,48E-05	0,000206	3,008731
NM_001031488	NTF3	0,677152	5,634918	1,49E-05	0,000206	3,006152
ENSGALT00000031381	LOC422106	1,147382	5,623466	1,53E-05	0,000211	2,980286
ENSGALT00000038405	EML4	-0,83934	-5,61616	1,55E-05	0,000214	2,963773
ENSGALT00000038805	DGKE	-0,91873	-5,60084	1,61E-05	0,000221	2,929141
ENSGALT00000014571	LOC428554	0,968158	5,595537	1,63E-05	0,000223	2,917153
ENSGALT00000020569	AGTPBP1	-0,59392	-5,59294	1,64E-05	0,000224	2,911272
ENSGALT00000016472	PLK3	0,482343	5,585455	1,66E-05	0,000227	2,894343
ENSGALT00000004236	NUP35	0,546355	5,58465	1,67E-05	0,000227	2,892521
ENSGALT00000023178	PCDH7	-0,73252	-5,58026	1,68E-05	0,000228	2,882595
ENSGALT00000009567	OPRL1	-1,07388	-5,5781	1,69E-05	0,000229	2,8777
ENSGALT00000041181	CLAT	0,880439	5,566777	1,74E-05	0,000234	2,852062
ENSGALT00000036528	LOC769538	-0,84843	-5,55761	1,77E-05	0,000238	2,831304
ENSGALT00000029971	O57534	0,624948	5,55529	1,78E-05	0,000239	2,826042
ENSGALT00000038428	Q5ZIL8	-0,69942	-5,54881	1,81E-05	0,000241	2,811355
ENSGALT00000014247	B3GNT5	-1,17345	-5,54783	1,81E-05	0,000241	2,809138
ENSGALT00000028059	SLITRK3	-1,03993	-5,54726	1,82E-05	0,000241	2,807836
ENSGALT00000026454	MTBP	0,641783	5,544501	1,83E-05	0,000242	2,80159
ENSGALT00000018020	PDE4B	-0,75781	-5,54226	1,84E-05	0,000243	2,796515
ENSGALT00000025626	Q90769	-1,12549	-5,53994	1,85E-05	0,000243	2,791244
ENSGALT00000013562	PHTF2	-0,57952	-5,53806	1,85E-05	0,000243	2,786994

ENSGALT00000007151	DMGDH	-0,88028	-5,53751	1,86E-05	0,000243	2,785744
ENSGALT00000037765	HMOX1	0,66446	5,53431	1,87E-05	0,000245	2,778486
ENSGALT00000005468	MCM10	0,709169	5,516671	1,95E-05	0,000254	2,738471
ENSGALT00000006053	CYFIP2	-0,76706	-5,51554	1,95E-05	0,000254	2,735906
ENSGALT00000020095	GRM1	-1,07404	-5,51232	1,97E-05	0,000255	2,728604
NM_205190	RET	-0,62098	-5,51133	1,97E-05	0,000255	2,726352
NM_001031246	BARD1	0,715421	5,503129	2,01E-05	0,000259	2,70773
NM_204349	MBL2	0,932767	5,5012	2,02E-05	0,00026	2,70335
NM_001079740	LOC769174	1,114783	5,495695	2,04E-05	0,000262	2,690848
ENSGALT00000021359	VWC2	-1,06225	-5,49303	2,06E-05	0,000263	2,684805
ENSGALT00000005282	C1QTNF3	1,107213	5,489482	2,07E-05	0,000265	2,676735
ENSGALT00000011947	SRRM4	-0,6573	-5,47693	2,13E-05	0,000272	2,648218
ENSGALT00000025600	CPNE3	-0,74328	-5,47508	2,14E-05	0,000272	2,644007
ENSGALT00000028361	GAS1	0,821982	5,46834	2,17E-05	0,000276	2,628683
ENSGALT00000014662	ZNF385B	0,804887	5,463788	2,2E-05	0,000277	2,618331
ENSGALT00000020275	LRP1B	-0,7228	-5,45879	2,22E-05	0,000279	2,606958
ENSGALT00000026781	PRPS2	-1,13127	-5,45797	2,23E-05	0,000279	2,6051
ENSGALT00000015417	PPAPDC1A	0,759374	5,457835	2,23E-05	0,000279	2,604792
ENSGALT00000024216	CYTL1	1,515243	5,451591	2,26E-05	0,000282	2,590588
NM_204610	CHAT	1,091791	5,450294	2,27E-05	0,000283	2,587635
ENSGALT00000039806	Q5ZKK6	-0,83282	-5,44066	2,32E-05	0,000287	2,565716
ENSGALT00000016200	FRS2	-0,7245	-5,43874	2,33E-05	0,000288	2,561347
ENSGALT00000025008	Q90Z42	0,74897	5,435697	2,34E-05	0,000289	2,554413
ENSGALT00000009331	COL7A1	0,664349	5,433259	2,36E-05	0,00029	2,548862
ENSGALT00000039541	SCO1	-0,67942	-5,43263	2,36E-05	0,00029	2,547423
ENSGALT00000017215	STON2	-0,84103	-5,42895	2,38E-05	0,000291	2,539041
NM_206906	ANXA1	0,48547	5,427731	2,39E-05	0,000291	2,536273
ENSGALT00000030862	PAG1	-0,6681	-5,42241	2,42E-05	0,000294	2,524159
ENSGALT00000007785	FRMPD3	-0,96827	-5,41657	2,45E-05	0,000297	2,510846
ENSGALT00000034015	LOC415495	0,748924	5,41437	2,46E-05	0,000298	2,505838
ENSGALT00000004729	PHGDH	1,658009	5,398088	2,55E-05	0,000307	2,468727
ENSGALT00000034863	KCNT1	-0,88653	-5,39704	2,56E-05	0,000307	2,466335
ENSGALT00000017310	EML5	-0,6496	-5,39624	2,57E-05	0,000307	2,464517
ENSGALT00000015450	FSTL5	-0,51319	-5,39568	2,57E-05	0,000307	2,463235
NM_001005346	TSKU	-0,7692	-5,39493	2,57E-05	0,000307	2,461535
ENSGALT00000023744	Q6TL25	1,356238	5,392973	2,58E-05	0,000307	2,457064
ENSGALT00000007671	TGM6	-0,72537	-5,38582	2,63E-05	0,000312	2,440755
ENSGALT00000007788	SLC6A11	-0,68614	-5,38357	2,64E-05	0,000312	2,435617
ENSGALT00000012682	ANKK1	-0,7799	-5,38022	2,66E-05	0,000313	2,42798
ENSGALT00000022131	VIP	1,70174	5,363108	2,77E-05	0,000325	2,388922
ENSGALT00000026582	TTC32	0,736748	5,359545	2,79E-05	0,000327	2,380788
NM_001012863	RBM24	0,757062	5,35851	2,8E-05	0,000327	2,378424
NM_214591	LGALS3	1,295727	5,348376	2,86E-05	0,000333	2,35528
ENSGALT00000024183	PGM5	0,744228	5,345843	2,88E-05	0,000335	2,349495
ENSGALT00000009496	NRIP3	-0,72504	-5,32735	3,01E-05	0,000347	2,307228
ENSGALT00000010135	RACGAP1	0,528683	5,325267	3,02E-05	0,000348	2,302475
ENSGALT00000021107	CDH12	0,961134	5,317568	3,07E-05	0,000354	2,284872
ENSGALT00000040286	LOC771030	0,68796	5,310671	3,12E-05	0,000359	2,269099
NM_001012880	PRIM2	1,097752	5,304181	3,17E-05	0,000363	2,254254
ENSGALT00000009188	Q9YI45	-0,91622	-5,30129	3,19E-05	0,000365	2,247632

ENSGALT00000036889	Q91429	-0,67716	-5,29236	3,26E-05	0,000372	2,227207
NM_204777	FGF14	-0,48863	-5,28638	3,3E-05	0,000376	2,213522
ENSGALT00000019447	MEI1	-0,72861	-5,27996	3,35E-05	0,00038	2,198817
ENSGALT00000015493	GNG11	0,86789	5,273399	3,4E-05	0,000385	2,183795
ENSGALT00000004163	SELP	0,564282	5,269077	3,44E-05	0,000388	2,173895
ENSGALT00000016250	LOC423317	0,562825	5,264752	3,47E-05	0,000389	2,163988
ENSGALT00000042220	gga-mir-1611	-0,69213	-5,26384	3,48E-05	0,000389	2,161904
NM_001079487	CBLN4	1,312779	5,263397	3,48E-05	0,000389	2,160885
ENSGALT00000039846	ACZ	-0,71082	-5,2621	3,49E-05	0,000389	2,157924
ENSGALT00000008237	PTPDC1	-0,57284	-5,26171	3,5E-05	0,000389	2,157021
ENSGALT00000023611	POLR3G	-0,50423	-5,26023	3,51E-05	0,000389	2,153623
NM_205140	SLC16A8	0,462504	5,259934	3,51E-05	0,000389	2,15295
ENSGALT00000012902	Q766Y2	0,61651	5,258546	3,52E-05	0,000389	2,149771
ENSGALT00000041416	gga-mir-193a	-0,81288	-5,25758	3,53E-05	0,000389	2,14755
ENSGALT00000038727	SPATA1	-0,51652	-5,25684	3,54E-05	0,000389	2,14586
ENSGALT00000027125	LOC771085	1,695994	5,255397	3,55E-05	0,00039	2,142554
NM_001177309	VIP	1,577086	5,250254	3,59E-05	0,000393	2,130768
ENSGALT00000014755		-0,76689	-5,24837	3,61E-05	0,000393	2,126455
ENSGALT00000040335	CD55	1,296025	5,248349	3,61E-05	0,000393	2,126401
ENSGALT00000024411	TRPM3	-1,11849	-5,23165	3,75E-05	0,000407	2,08812
ENSGALT00000022312	PUR1	-4,46057	-5,23016	3,76E-05	0,000408	2,084692
ENSGALT00000038802	ZNF385B	0,71003	5,223493	3,82E-05	0,000413	2,069406
NR_035027	MIR216C	0,826077	5,22284	3,83E-05	0,000413	2,067908
ENSGALT00000011513	Q8JGT3	-0,87695	-5,219	3,86E-05	0,000416	2,059098
ENSGALT00000035225	LOC419834	-0,60858	-5,20788	3,96E-05	0,000426	2,033575
ENSGALT00000006386	DHRS13	0,676923	5,20583	3,98E-05	0,000427	2,028877
ENSGALT00000027569	KL	-0,98119	-5,19763	4,05E-05	0,000433	2,010061
ENSGALT00000012944	TRPC5	-0,609	-5,18782	4,15E-05	0,000442	1,987524
NM_001012611	EDAR	0,719483	5,184032	4,18E-05	0,000444	1,978828
ENSGALT00000031027	SPB10	0,762867	5,182005	4,2E-05	0,000445	1,974171
NM_206991	ADIPOQ	0,742266	5,181646	4,21E-05	0,000445	1,973346
NM_204687	OPCML	-0,63693	-5,18026	4,22E-05	0,000446	1,970157
ENSGALT00000037975	Q9I8D4	0,747498	5,177827	4,25E-05	0,000446	1,964574
ENSGALT00000013779	CAC1B	-0,47012	-5,17507	4,27E-05	0,000448	1,958243
ENSGALT00000020659	Q5ZKC0	0,595087	5,16977	4,33E-05	0,000453	1,946063
ENSGALT00000022492	IL22RA2	-1,32166	-5,16393	4,38E-05	0,000458	1,932641
NM_001146044	CDA	-0,65939	-5,15806	4,44E-05	0,000463	1,919148
NM_001030763	C12orf48	0,548371	5,153988	4,49E-05	0,000466	1,90979
ENSGALT00000041124	Q9PS75	-0,54418	-5,14859	4,54E-05	0,000471	1,897371
ENSGALT00000031959	Q5ZJK2	-0,70322	-5,14306	4,6E-05	0,000474	1,884655
ENSGALT00000005090	LGALS2	0,78363	5,142804	4,6E-05	0,000474	1,884073
ENSGALT00000034646	C9K601	-1,24174	-5,14081	4,63E-05	0,000475	1,879478
ENSGALT00000021718	SYT7	-0,67023	-5,12842	4,76E-05	0,000487	1,850997
ENSGALT00000034909	SSH2	-0,68604	-5,12253	4,83E-05	0,000493	1,837429
ENSGALT00000042479	gga-mir-1551	-0,73442	-5,12159	4,84E-05	0,000493	1,835279
ENSGALT00000012609	FADS6	0,693688	5,117016	4,89E-05	0,000497	1,824745
ENSGALT00000038848	LOC415852	0,838739	5,115461	4,91E-05	0,000498	1,821168
ENSGALT00000032415	ZNF804B	-0,6639	-5,10796	4,99E-05	0,000506	1,803901
ENSGALT00000027777	ANGPTL5	1,01307	5,105739	5,02E-05	0,000508	1,798788
NM_001030826	RNASEH2B	-0,65616	-5,09758	5,11E-05	0,000516	1,779995

ENSGALT00000037871	CBX7	-1,16789	-5,0933	5,16E-05	0,00052	1,77014
ENSGALT00000016124	EML4	-0,66282	-5,09189	5,18E-05	0,00052	1,766904
ENSGALT00000038004	HPSE	0,870525	5,091845	5,18E-05	0,00052	1,766795
NM_204914	LOC395744	-0,74567	-5,08501	5,26E-05	0,000527	1,751053
ENSGALT00000032184	PLAC8	-0,7517	-5,07952	5,33E-05	0,000532	1,738404
ENSGALT00000023689	ZBTB7C	-0,67601	-5,07785	5,35E-05	0,000533	1,73456
ENSGALT00000020475	GALNT13	-0,49735	-5,07659	5,37E-05	0,000534	1,731644
ENSGALT00000031564		1,029467	5,067759	5,48E-05	0,000542	1,711303
ENSGALT00000037706	Q9PUC7	-0,84713	-5,06284	5,54E-05	0,000548	1,699974
NM_001034814	CETP	0,715318	5,061981	5,55E-05	0,000548	1,697986
ENSGALT00000038421	RTDR1	0,942225	5,057584	5,61E-05	0,000552	1,687851
ENSGALT00000036532	LOC769538	-0,76648	-5,05587	5,63E-05	0,000553	1,683903
ENSGALT00000031521	Q3L3P8	-1,07367	-5,05219	5,68E-05	0,000556	1,675417
ENSGALT00000042225	gga-mir-1550	-0,88003	-5,05204	5,68E-05	0,000556	1,67506
NM_001001777	GSTA3	0,970923	5,051204	5,7E-05	0,000556	1,673142
NM_001044636	RASD1	-0,58286	-5,04849	5,73E-05	0,000559	1,66689
NM_001162892	COPN5L2	1,432092	5,041868	5,82E-05	0,000566	1,651613
ENSGALT00000038338	C2orf34	0,868634	5,040703	5,84E-05	0,000567	1,648925
ENSGALT00000040664	Q4TZW6	-0,9092	-5,03922	5,86E-05	0,000568	1,645507
ENSGALT00000015034	TLR21	0,514704	5,038391	5,87E-05	0,000568	1,643592
ENSGALT00000005616	C20orf95	-0,84632	-5,03401	5,93E-05	0,000572	1,633489
ENSGALT00000020885	LY86	1,359128	5,029403	5,99E-05	0,000577	1,62286
ENSGALT00000042213	gga-mir-1772	-0,60159	-5,02401	6,07E-05	0,000584	1,610423
ENSGALT00000033414	MSMB	0,787824	5,017786	6,16E-05	0,000591	1,596056
ENSGALT00000024917	DUSP27	1,120999	5,00043	6,41E-05	0,000614	1,555995
NM_213585	LOC407087	1,117752	4,997523	6,45E-05	0,000617	1,549282
NM_205329	SRL	0,568058	4,99659	6,47E-05	0,000617	1,547129
ENSGALT00000033524	AURKA	0,566049	4,991645	6,54E-05	0,000623	1,535712
ENSGALT00000027850	Q6V0P4	-0,82616	-4,98931	6,58E-05	0,000625	1,530327
ENSGALT00000010993	ADARB2	-0,75545	-4,98864	6,59E-05	0,000625	1,528779
ENSGALT00000027848	Q6V0P4	-0,69508	-4,9843	6,66E-05	0,000631	1,518742
ENSGALT00000019713	LMLN	-0,73819	-4,98013	6,72E-05	0,000636	1,509122
ENSGALT00000008952	SEZ6L	-0,50679	-4,97717	6,77E-05	0,000638	1,502278
ENSGALT00000042112	gga-mir-1555	-0,69211	-4,9767	6,77E-05	0,000638	1,501197
ENSGALT00000042414	gga-mir-1705	-0,87684	-4,97439	6,81E-05	0,000641	1,495849
ENSGALT00000040526	LOC430516	0,520977	4,973363	6,83E-05	0,000641	1,493485
ENSGALT00000020844	O13111	0,78556	4,970967	6,87E-05	0,000643	1,487949
ENSGALT00000040171	RHOC	0,584454	4,968938	6,9E-05	0,000645	1,483262
ENSGALT00000028013	DCHS1	-0,92055	-4,96609	6,94E-05	0,000648	1,476686
ENSGALT00000018326	BEGAIN	-0,80497	-4,96363	6,98E-05	0,000651	1,471005
ENSGALT00000007223	EFNB1	0,741468	4,963014	6,99E-05	0,000651	1,469573
ENSGALT00000034345	RANBP3L	-0,76382	-4,95675	7,1E-05	0,000657	1,455106
ENSGALT00000036461	KL	-0,95731	-4,95625	7,11E-05	0,000657	1,453942
ENSGALT00000019963	LOC776557	-0,69971	-4,95542	7,12E-05	0,000657	1,45203
NM_001039605	PRKAA2	-0,89713	-4,95277	7,16E-05	0,00066	1,445895
ENSGALT00000031534	B3XZF6	1,50863	4,948705	7,23E-05	0,000665	1,436503
ENSGALT00000005010	LOC772306	-0,48185	-4,94818	7,24E-05	0,000665	1,435292
ENSGALT00000038594		-1,11647	-4,94391	7,31E-05	0,00067	1,425422
ENSGALT00000014558	NOXA1	0,635679	4,943631	7,32E-05	0,00067	1,424773
NM_204149	NBL1	0,462033	4,941646	7,35E-05	0,000672	1,420184

NM_001030561	PLLP	-0,93218	-4,9253	7,64E-05	0,000693	1,382395
NM_204863	PLEK	0,708556	4,925142	7,64E-05	0,000693	1,382021
ENSGALT00000022419	NMBR	1,014306	4,922131	7,69E-05	0,000697	1,375057
ENSGALT00000026104		0,944006	4,916911	7,79E-05	0,000704	1,362983
NM_205361	LOC396318	-0,74245	-4,90791	7,95E-05	0,000718	1,34216
ENSGALT00000022037	MR1	0,734617	4,905788	7,99E-05	0,00072	1,337252
NM_205060	LOC395933	0,924072	4,904549	8,02E-05	0,000721	1,334383
ENSGALT00000027863	NOX4	-1,29564	-4,90118	8,08E-05	0,000725	1,326588
ENSGALT00000032368	Q9YGQ6	-0,649	-4,89457	8,21E-05	0,000735	1,3113
ENSGALT00000042463	gga-mir-1774	-0,5837	-4,89248	8,25E-05	0,000738	1,306447
ENSGALT00000005545	IOV7	-1,26303	-4,89072	8,28E-05	0,000739	1,302392
ENSGALT00000012493	LOC422321	0,751449	4,887725	8,34E-05	0,000743	1,29545
ENSGALT00000040748	Q5F358	-0,77595	-4,88346	8,42E-05	0,000749	1,285573
ENSGALT00000038734	Q9YGP8	-0,74888	-4,87638	8,56E-05	0,00076	1,269185
ENSGALT00000009820	GPC3	1,114418	4,87474	8,6E-05	0,00076	1,265392
NM_001031535	NPY1R	1,327194	4,874312	8,6E-05	0,00076	1,2644
NM_213580	MUSTN1	1,209251	4,872953	8,63E-05	0,000761	1,261252
ENSGALT00000037166	SCNNA	-0,81703	-4,86844	8,72E-05	0,000767	1,250793
NM_204105	SDK1	-0,68353	-4,86382	8,82E-05	0,000772	1,240102
ENSGALT00000040819	IOV7	-1,18286	-4,86089	8,88E-05	0,000775	1,23332
NM_204707	SALL1	0,889772	4,86085	8,88E-05	0,000775	1,233227
ENSGALT00000026044	GPR20	0,816874	4,855348	8,99E-05	0,000784	1,220485
ENSGALT00000008076	TEX14	-0,95489	-4,8525	9,05E-05	0,000788	1,213894
ENSGALT00000030617	LOC417973	1,76664	4,848111	9,15E-05	0,000795	1,203724
ENSGALT00000020485	RASD2	0,715039	4,845987	9,19E-05	0,000797	1,198802
ENSGALT00000042150	SNORD82	-0,75387	-4,84229	9,27E-05	0,000803	1,190232
ENSGALT00000015770	Q98940	0,528031	4,839529	9,33E-05	0,000807	1,183842
NM_001109762	NTF3	0,558054	4,836734	9,4E-05	0,00081	1,177365
ENSGALT00000040754	Q90818	-0,68584	-4,82782	9,59E-05	0,000826	1,156718
ENSGALT00000017720	TMEM161B	-0,53439	-4,82297	9,7E-05	0,000834	1,145484
ENSGALT00000024585	O57411	0,794254	4,82204	9,72E-05	0,000835	1,143316
ENSGALT00000037382	FABP7	0,75577	4,820936	9,75E-05	0,000835	1,140758
ENSGALT00000009269	ZCCHC14	-0,87447	-4,81928	9,79E-05	0,000835	1,136916
NM_205094	TFAP2A	0,827188	4,818923	9,8E-05	0,000835	1,136094
ENSGALT00000022994	PHF21B	-0,59299	-4,8149	9,89E-05	0,00084	1,126758
ENSGALT00000004950	DGKE	-1,03318	-4,81172	9,96E-05	0,000845	1,119393
ENSGALT00000008484	KBTBD5	-0,61574	-4,81086	9,98E-05	0,000845	1,117411
ENSGALT00000024666	C6orf182	0,555671	4,806946	0,000101	0,00085	1,108332
ENSGALT00000031513	LOC768783	-0,60274	-4,80323	0,000102	0,000856	1,099727
ENSGALT00000013642	ALS2CR2	0,485779	4,800059	0,000102	0,000861	1,092365
ENSGALT00000025295	UGCG	-0,60609	-4,79901	0,000103	0,000862	1,089933
ENSGALT00000026984	Q5ZIV4	0,650654	4,79459	0,000104	0,00087	1,079686
ENSGALT00000022132	VIP	1,477605	4,792634	0,000104	0,000871	1,075149
ENSGALT00000037894	Q9I8D5	0,639747	4,792512	0,000104	0,000871	1,074866
ENSGALT00000015791	KLHL23	0,61292	4,791813	0,000104	0,000871	1,073247
ENSGALT00000019973	PPIL5	0,396848	4,789204	0,000105	0,000874	1,067198
NM_001145016	C20orf95	-0,77351	-4,78901	0,000105	0,000874	1,066739
NM_001142254	DLK1	-0,62839	-4,78813	0,000105	0,000874	1,064713
ENSGALT00000035067	LOC419892	0,56826	4,786797	0,000106	0,000875	1,061616
ENSGALT00000040903		-0,67377	-4,7854	0,000106	0,000877	1,058367

ENSGALT00000040309	TMEM88	-1,02692	-4,7849	0,000106	0,000877	1,057222
ENSGALT00000021249	CAC1C	-0,71573	-4,7766	0,000108	0,000892	1,037973
ENSGALT00000008029	AIPL1	-0,80501	-4,77509	0,000109	0,000894	1,034457
ENSGALT00000004464	UNC80	-0,80064	-4,77131	0,00011	0,000901	1,025705
NM_001194983	PTGES	0,610525	4,769446	0,00011	0,000903	1,021373
ENSGALT00000009577	ANXA8	1,806256	4,767589	0,00011	0,000906	1,017067
NM_001101829	NOX4	-0,86112	-4,76401	0,000111	0,000912	1,008766
ENSGALT00000023581	CAC1C	-0,60066	-4,75684	0,000113	0,000926	0,992135
ENSGALT00000031660	LOC421855	0,494361	4,753429	0,000114	0,000932	0,984216
ENSGALT00000035401	CCDC39	0,806605	4,751047	0,000115	0,000935	0,97869
ENSGALT00000014609	VWA2	0,87446	4,749269	0,000115	0,000936	0,974564
ENSGALT00000027761	O93363	-1,20958	-4,7491	0,000115	0,000936	0,974168
ENSGALT00000017601	Q2LAI0	-0,81595	-4,74869	0,000115	0,000936	0,973217
ENSGALT00000019043	ACPP	-1,04649	-4,74552	0,000116	0,000941	0,965874
NM_001038691	ZDHHC8	-0,72318	-4,74475	0,000117	0,000941	0,964089
ENSGALT00000006172	ERCC6L	-0,64857	-4,74427	0,000117	0,000941	0,962961
NM_205191	CRYBB3	1,014892	4,741435	0,000117	0,000946	0,956386
ENSGALT00000023900	Q91002	0,827391	4,733492	0,00012	0,000961	0,937954
ENSGALT00000040234	LOC769442	-0,61995	-4,72888	0,000121	0,000969	0,927252
ENSGALT00000016690	LTBP2	0,769553	4,728727	0,000121	0,000969	0,926894
ENSGALT00000020086	C22orf23	0,647241	4,726453	0,000122	0,000969	0,921615
ENSGALT00000005772	LOC427491	0,386009	4,726329	0,000122	0,000969	0,921328
ENSGALT00000033846	AL1A1	0,789035	4,726133	0,000122	0,000969	0,920873
ENSGALT00000018629	DUS4	-0,55059	-4,72583	0,000122	0,000969	0,920177
ENSGALT00000040133	PLXDC1	0,758418	4,724769	0,000122	0,000969	0,917708
ENSGALT00000020592	Q9I8D4	0,657661	4,72434	0,000122	0,000969	0,916712
ENSGALT00000016007	BBS5	0,378195	4,723799	0,000122	0,000969	0,915456
ENSGALT00000006472	GPR119	-0,6375	-4,72188	0,000123	0,000972	0,910999
NM_001045838	XCR1	0,778448	4,721072	0,000123	0,000972	0,909125
ENSGALT00000033606	GPR52	-0,77122	-4,71939	0,000124	0,000975	0,905217
ENSGALT00000030264	CSF2RA	0,874298	4,711194	0,000126	0,000991	0,886194
ENSGALT00000042557	U4	-0,81339	-4,70727	0,000127	0,000997	0,877076
ENSGALT00000005950		-0,70831	-4,70397	0,000128	0,001003	0,86943
NM_204504	BHLHB4	-0,69713	-4,70042	0,000129	0,00101	0,861177
ENSGALT00000020886	Q9DER6	0,876442	4,699419	0,00013	0,001011	0,858856
ENSGALT00000030205	Q9IAI5	-0,46371	-4,68923	0,000133	0,001034	0,835189
ENSGALT00000010471	HTR7	0,70614	4,684969	0,000134	0,001042	0,825302
ENSGALT00000020003	LOC430793	-0,81655	-4,68318	0,000135	0,001044	0,82114
ENSGALT00000038023	PON2	0,692495	4,682819	0,000135	0,001044	0,82031
ENSGALT00000039147	COHA1	1,104603	4,673478	0,000138	0,001063	0,798615
ENSGALT00000025209	HTT	-0,57165	-4,67182	0,000138	0,001065	0,794776
ENSGALT00000022138	FBXO5	0,521467	4,668688	0,000139	0,001072	0,78749
ENSGALT00000039618	SSH2	-0,86759	-4,66499	0,000141	0,001079	0,778907
ENSGALT00000015790	PDK4	-0,94058	-4,66351	0,000141	0,001081	0,77546
ENSGALT00000023658	Q75W81	0,683598	4,658174	0,000143	0,001093	0,763067
ENSGALT00000027749	Q90858	-0,54781	-4,65763	0,000143	0,001093	0,761814
ENSGALT00000017513	HPGD	0,74929	4,656261	0,000143	0,001095	0,758624
ENSGALT00000020785	EDN1	0,53317	4,655767	0,000144	0,001095	0,757476
ENSGALT00000034598	IOV7	-1,21387	-4,65516	0,000144	0,001095	0,756066
NM_204736	MARCO	0,708862	4,650118	0,000146	0,001106	0,744351

NM_001030633	GTLF3B	0,429848	4,647189	0,000147	0,001112	0,737548
ENSGALT00000034096	LRRTM3	-0,62164	-4,64617	0,000147	0,001113	0,735173
ENSGALT00000014800	UAP1L1	-0,69526	-4,64512	0,000147	0,001113	0,732744
ENSGALT00000024028	TYMS	0,429609	4,645061	0,000147	0,001113	0,732604
ENSGALT00000016558	SLC1A1	-0,5456	-4,64279	0,000148	0,001114	0,727331
ENSGALT00000038891	RPGP2	-0,71365	-4,64277	0,000148	0,001114	0,727285
ENSGALT00000014414	CXorf39	-0,86191	-4,64267	0,000148	0,001114	0,727055
ENSGALT00000017742	PRKG2	-0,7215	-4,6396	0,000149	0,00112	0,719925
NM_205046	LOC395914	0,958829	4,638129	0,00015	0,001123	0,716497
ENSGALT00000020209	HNMT	1,414313	4,637481	0,00015	0,001123	0,714992
ENSGALT00000023212	APOA5	0,544164	4,634557	0,000151	0,001129	0,708197
ENSGALT00000039474	IFNA1	-1,09444	-4,63025	0,000153	0,001139	0,69819
NM_001012898	CPT1A	-0,75239	-4,62965	0,000153	0,001139	0,696802
ENSGALT00000017443	NEIL3	0,557051	4,627502	0,000154	0,001143	0,691804
ENSGALT00000004709	SPHKAP	-0,64062	-4,62545	0,000154	0,001145	0,68704
ENSGALT00000010690	ATP11C	-0,48337	-4,62212	0,000155	0,001152	0,679305
ENSGALT00000007468	LOC422171	-0,66058	-4,62048	0,000156	0,001153	0,675495
NM_204896	BARX2	0,891126	4,61781	0,000157	0,001159	0,669282
NM_001012997	CEL	0,715913	4,61727	0,000157	0,001159	0,668028
ENSGALT00000040377	LOC430986	-1,35241	-4,61472	0,000158	0,001164	0,662104
ENSGALT00000029035	gga-mir-103-2	-0,62982	-4,60862	0,000161	0,001179	0,647917
ENSGALT00000007316	ZBTB37	-0,8704	-4,60712	0,000161	0,00118	0,644434
ENSGALT00000020058	LOC777160	0,78798	4,606244	0,000161	0,00118	0,642403
NM_204144	TBX20	-0,71056	-4,60594	0,000162	0,00118	0,641695
ENSGALT00000011346	ZBTB16	-0,56244	-4,60541	0,000162	0,00118	0,640471
NM_001030375	MATN1	0,784234	4,60522	0,000162	0,00118	0,640023
ENSGALT00000036430	Q90858	-0,5375	-4,60476	0,000162	0,00118	0,63896
ENSGALT00000023151	AR2BP	-0,71358	-4,60077	0,000164	0,001189	0,629677
ENSGALT00000015416	NEUS	-0,57001	-4,59742	0,000165	0,001197	0,621901
ENSGALT00000008848	CRBB3	0,965065	4,596853	0,000165	0,001197	0,620575
ENSGALT00000008039	GP9	1,022855	4,589372	0,000168	0,001215	0,603187
ENSGALT00000037903	PUR8	-0,59104	-4,58862	0,000168	0,001215	0,601443
ENSGALT00000009629	AQP8	0,756943	4,585489	0,00017	0,001222	0,59416
ENSGALT00000031528	CLIC5	0,877262	4,580256	0,000172	0,001235	0,581995
ENSGALT00000008738	PALMD	0,688256	4,579943	0,000172	0,001235	0,581269
ENSGALT00000019962	PDE1C	-0,52186	-4,57857	0,000172	0,001237	0,57807
ENSGALT00000010874	TRIM29	-0,60995	-4,57728	0,000173	0,001239	0,575079
ENSGALT00000040332	RBPMS	0,4214	4,572489	0,000175	0,001251	0,56394
ENSGALT00000036431	Q90858	-0,50935	-4,5696	0,000176	0,001256	0,557214
ENSGALT00000027062	LYG2	0,875023	4,569175	0,000176	0,001256	0,556235
ENSGALT00000038362	LARG2	0,428994	4,564778	0,000178	0,001267	0,546013
ENSGALT00000014227	MCF2L2	-0,64299	-4,56355	0,000178	0,001269	0,543151
ENSGALT00000005433	AGXT2	-0,76058	-4,56121	0,000179	0,001274	0,537711
NM_001080869	VAPA	-0,38751	-4,55704	0,000181	0,001285	0,528012
NM_204187	CDH7	-0,71539	-4,556	0,000182	0,001287	0,525607
NM_001146131	SLC22A4	-0,796	-4,55081	0,000184	0,001301	0,513544
ENSGALT00000015569	PLEK2	0,923428	4,549666	0,000184	0,001302	0,510877
ENSGALT00000014930	LOC770038	1,559534	4,543763	0,000187	0,001319	0,497153
NM_204897	SERPINB10	0,625787	4,541966	0,000188	0,001321	0,492976
ENSGALT00000041280	KANK3	0,647408	4,541766	0,000188	0,001321	0,49251

ENSGALT00000008322	Q90796	-0,71273	-4,53848	0,000189	0,00133	0,484877
ENSGALT00000023926	JMY	-0,50963	-4,53625	0,00019	0,001333	0,479684
ENSGALT00000027705	ZC3H12C	-0,74052	-4,53442	0,000191	0,001337	0,475427
ENSGALT00000014975	Q5F3V9	-0,47288	-4,52245	0,000197	0,001373	0,447604
ENSGALT00000036251	Q9YGQ9	-0,99243	-4,52048	0,000198	0,001378	0,44302
ENSGALT00000038922	Q5IHK1	-0,73987	-4,51647	0,000199	0,001385	0,433678
ENSGALT00000011466	Q6B842	-0,71284	-4,51646	0,000199	0,001385	0,433666
NM_001030639	PLK1	0,548998	4,515432	0,0002	0,001387	0,431274
ENSGALT00000036386	NARE	0,799755	4,513578	0,000201	0,001387	0,426963
ENSGALT00000042489	gga-mir-1715	-0,5807	-4,51336	0,000201	0,001387	0,426464
ENSGALT00000026654	PHKA2	-0,44255	-4,51308	0,000201	0,001387	0,425806
ENSGALT00000027122	C2orf40	0,703825	4,512062	0,000202	0,001388	0,423438
ENSGALT00000009166	PKD1	-0,60031	-4,51058	0,000202	0,00139	0,419985
ENSGALT00000038045	LOC430793	-0,81107	-4,50998	0,000203	0,00139	0,418605
NM_001031055	TTC7A	-0,65121	-4,50877	0,000203	0,001392	0,415789
ENSGALT00000017607	CDCA7	0,568077	4,50791	0,000204	0,001393	0,413782
NM_205314	CDC2	0,534546	4,504751	0,000205	0,001401	0,406435
ENSGALT00000016119	KIAA0240	-0,6079	-4,49894	0,000208	0,001419	0,392911
ENSGALT00000013855	ACZ	-0,59064	-4,49817	0,000208	0,001419	0,391122
ENSGALT00000026450	RSAD2	1,031051	4,493412	0,000211	0,001432	0,380066
ENSGALT00000040319	C1orf70	-0,52847	-4,49339	0,000211	0,001432	0,380009
ENSGALT00000017942	LOC776434	-0,54633	-4,49205	0,000211	0,001432	0,376894
ENSGALT00000005680	FTO	0,618734	4,491987	0,000211	0,001432	0,376751
ENSGALT00000004943	Q9W6F6	0,577979	4,491484	0,000212	0,001432	0,375582
ENSGALT00000035322	gga-mir-147-2	0,778499	4,475641	0,00022	0,001481	0,338736
ENSGALT00000016803	JDP2	0,465261	4,475557	0,00022	0,001481	0,338542
ENSGALT00000033832	C20orf195	0,681794	4,47286	0,000221	0,001487	0,332267
ENSGALT00000019332	LIMD1	0,46361	4,47204	0,000221	0,001488	0,330362
ENSGALT00000039737	LOC771702	0,463774	4,471296	0,000222	0,001488	0,328631
ENSGALT00000006252	MYPN	0,991948	4,469723	0,000223	0,001492	0,324972
ENSGALT00000020670	ALDH5A1	-0,72092	-4,46617	0,000225	0,001502	0,316706
ENSGALT00000012903	Q766Y2	0,532809	4,465701	0,000225	0,001502	0,315617
ENSGALT00000032904	NXPH2	1,031341	4,460368	0,000228	0,001519	0,303214
ENSGALT00000026124	PDXK	-0,67926	-4,4565	0,00023	0,00153	0,294213
ENSGALT00000005510	BHLHB8	-0,57803	-4,45459	0,000231	0,001534	0,289787
ENSGALT00000039379	CEL	0,679806	4,448982	0,000234	0,001549	0,276733
ENSGALT00000025589	TTK	0,48239	4,448806	0,000234	0,001549	0,276323
ENSGALT00000024611	COL8A1	0,661884	4,448717	0,000234	0,001549	0,276116
NM_001007821	PRRX1	-0,64288	-4,44479	0,000236	0,001562	0,266983
ENSGALT00000019845	TBX20	-0,67072	-4,44203	0,000238	0,00157	0,26056
ENSGALT00000014074	LOC417741	0,448968	4,435856	0,000241	0,001589	0,246204
ENSGALT00000012782	Q5ZJ18	-0,65431	-4,43453	0,000242	0,001592	0,243109
ENSGALT00000009261	PKD2L1	0,648941	4,433491	0,000243	0,001594	0,240703
ENSGALT00000021912	GAN	-0,77867	-4,43288	0,000243	0,001594	0,239281
NR_031441	MIR128-1	-0,7315	-4,43139	0,000244	0,001597	0,235819
ENSGALT00000025644	CD109	-0,65498	-4,43076	0,000244	0,001598	0,234346
ENSGALT00000023623	ARRDC3	-0,54835	-4,4287	0,000245	0,001603	0,229571
ENSGALT00000040083	CAC1B	-0,77158	-4,42797	0,000246	0,001603	0,22787
NM_204766	MYH15	-0,86662	-4,42734	0,000246	0,001603	0,226402
ENSGALT00000007377	LOC427547	-0,63062	-4,42708	0,000246	0,001603	0,2258

ENSGALT00000031724	FAM162B	-0,66858	-4,42513	0,000247	0,001609	0,221252
ENSGALT00000026481	DSCC1	0,510178	4,424002	0,000248	0,001611	0,218634
ENSGALT00000023622	GPR98	-0,51246	-4,41719	0,000252	0,001633	0,202797
ENSGALT00000041877	SNORA53	-0,69082	-4,41217	0,000255	0,001648	0,19112
ENSGALT00000015261	Q9PVI7	-0,4773	-4,41008	0,000256	0,001654	0,186261
ENSGALT00000027549	CCNA1	1,115049	4,409029	0,000257	0,001656	0,183809
NM_001001782	MPL	0,710626	4,401667	0,000262	0,001681	0,166689
ENSGALT00000035454	gga-mir-147-2	0,742063	4,401499	0,000262	0,001681	0,166298
NM_001030612	LOC416235	-1,21378	-4,4011	0,000262	0,001681	0,165378
ENSGALT00000034690	DMRT1	-0,78237	-4,39907	0,000263	0,001687	0,160648
ENSGALT00000036825	CABYR	-0,57714	-4,39539	0,000265	0,0017	0,152081
NM_001031553	BDKRB2	-0,87746	-4,39309	0,000267	0,001707	0,146736
ENSGALT00000006106	KRT10	-0,65166	-4,39177	0,000268	0,00171	0,143663
ENSGALT00000018105	WDR78	0,860826	4,387851	0,00027	0,001724	0,134556
ENSGALT00000038533	LOC771944	-0,74531	-4,38707	0,000271	0,001725	0,132751
ENSGALT00000029039	gga-mir-128-1	-0,72114	-4,37944	0,000276	0,001754	0,114987
NM_205173	RAD51	0,461833	4,37781	0,000277	0,001759	0,111204
ENSGALT00000037538	DMXL1	-0,76457	-4,37372	0,000279	0,001773	0,101683
ENSGALT00000033236	RHO	-0,69462	-4,37283	0,00028	0,001775	0,099633
ENSGALT00000008462	LOC424816	-1,06748	-4,3698	0,000282	0,001786	0,092578
ENSGALT00000004672	SLC12A3	-0,72488	-4,36576	0,000285	0,0018	0,083187
NM_204794	ESR2	0,81861	4,358293	0,00029	0,00183	0,065817
ENSGALT00000020138	KCTD16	-0,69527	-4,35268	0,000294	0,001852	0,052767
ENSGALT00000028532	SNORA46	-0,59596	-4,34435	0,000299	0,001886	0,033393
ENSGALT00000006522	SSH2	-0,62048	-4,34402	0,0003	0,001886	0,032628
ENSGALT00000039714		-0,48278	-4,34013	0,000302	0,001901	0,023573
ENSGALT00000041294	ACCN2	-0,7285	-4,33906	0,000303	0,001903	0,02109
NM_001006479	ARHGAP11A	0,424559	4,336829	0,000305	0,00191	0,015906
ENSGALT00000021577	CENPE	0,489976	4,326672	0,000312	0,001948	-0,00771
ENSGALT00000014178	Q90VY6	0,525991	4,324799	0,000314	0,001954	-0,01206
ENSGALT00000036273	CCDC48	0,804992	4,322158	0,000316	0,001964	-0,0182
ENSGALT00000034881		-0,90553	-4,32164	0,000316	0,001964	-0,01941
NM_001031071	GPR126	-0,80021	-4,32104	0,000316	0,001965	-0,02079
ENSGALT00000023389	LEPREL2	-0,49651	-4,31592	0,00032	0,001986	-0,03272
ENSGALT00000015193		-1,36102	-4,31498	0,000321	0,001987	-0,03488
ENSGALT00000028294	RIT2	0,631759	4,314155	0,000322	0,001987	-0,03681
NM_001004403	SLBP	0,528441	4,314111	0,000322	0,001987	-0,03691
ENSGALT00000038635	Q91002	0,780354	4,310895	0,000324	0,001997	-0,04439
ENSGALT00000032417	TMEM28	-0,63345	-4,30997	0,000325	0,001997	-0,04655
ENSGALT00000009667	HOMER2	0,460852	4,307576	0,000327	0,002006	-0,0521
ENSGALT00000030547	Q3L255	0,523711	4,305609	0,000328	0,002013	-0,05668
ENSGALT00000024319	CABYR	-0,61061	-4,30319	0,00033	0,002021	-0,0623
ENSGALT00000010311	IFIT5	0,970619	4,302999	0,00033	0,002021	-0,06274
ENSGALT00000033989	C20orf58	-0,52432	-4,30124	0,000332	0,002024	-0,06683
ENSGALT00000034603	Q91006	-0,43814	-4,30106	0,000332	0,002024	-0,06725
ENSGALT00000014179	Q90VY6	0,479822	4,300684	0,000332	0,002024	-0,06813
NM_001012603	GINS1	0,498398	4,299481	0,000333	0,002025	-0,07092
ENSGALT00000042544	NRON	-0,78889	-4,29947	0,000333	0,002025	-0,07095
ENSGALT00000036969	GFRAL	-0,58422	-4,29404	0,000337	0,002049	-0,08357
ENSGALT00000025428	PMP2	-1,18814	-4,29342	0,000338	0,00205	-0,08502

NR_031395	MIR33	-0,55122	-4,29201	0,000339	0,002054	-0,08829
ENSGALT00000005897	Q9IAM2	-0,39054	-4,28787	0,000342	0,002067	-0,09791
ENSGALT00000010763	Q6VXX8	0,889098	4,2826	0,000346	0,002082	-0,11016
ENSGALT00000027155	FAM70B	0,688679	4,282522	0,000347	0,002082	-0,11034
ENSGALT00000039356	LAMB1	-0,8189	-4,2818	0,000347	0,002082	-0,11203
ENSGALT00000035097	A2PYM3	0,436358	4,281709	0,000347	0,002082	-0,11223
ENSGALT00000033938	CCDC48	0,747992	4,281209	0,000348	0,002082	-0,11339
ENSGALT00000010168	Q03793	0,889038	4,279018	0,000349	0,002091	-0,11849
NM_205498	IL8	0,571395	4,275359	0,000352	0,002104	-0,12699
ENSGALT00000008145	VINC	0,532391	4,264419	0,000362	0,002157	-0,15242
NM_001012786	FBXL18	-0,66491	-4,26173	0,000364	0,002167	-0,15866
NM_205099	LOC395991	-1,22965	-4,26146	0,000364	0,002167	-0,15928
ENSGALT00000039710	ASCL3	-0,47339	-4,26033	0,000365	0,002167	-0,16193
ENSGALT00000019765	C14orf108	0,508831	4,258588	0,000367	0,002174	-0,16597
ENSGALT00000007194	BHMT	-0,51651	-4,25436	0,00037	0,002193	-0,17579
ENSGALT00000022388	O93526	0,77713	4,252898	0,000372	0,002195	-0,17919
NM_001039318	SKI	-0,62079	-4,25287	0,000372	0,002195	-0,17926
ENSGALT00000037454	TMEM179	-0,73106	-4,25262	0,000372	0,002195	-0,17984
ENSGALT00000018602	HOP	0,676048	4,251124	0,000373	0,002199	-0,18331
ENSGALT00000039014	Q7T191	0,541439	4,250851	0,000373	0,002199	-0,18394
ENSGALT00000014902	FCN2	-0,41772	-4,24982	0,000374	0,002201	-0,18635
ENSGALT00000013810	ALS2CR8	-0,53428	-4,24861	0,000375	0,002205	-0,18916
ENSGALT00000008553	GJC2	0,662925	4,246472	0,000377	0,002214	-0,19412
ENSGALT00000014654	AK5	0,784432	4,245133	0,000379	0,002218	-0,19723
ENSGALT00000025054	LOC427306	0,763918	4,241918	0,000381	0,002233	-0,2047
NM_001113186	GRIA4	-0,41375	-4,24122	0,000382	0,002233	-0,20631
ENSGALT00000022794	SYTL2	-0,5121	-4,24072	0,000383	0,002233	-0,20749
ENSGALT00000032827	CNTLN	-0,41741	-4,23968	0,000383	0,002233	-0,2099
NM_204933	CDD	0,767481	4,239418	0,000384	0,002233	-0,21051
ENSGALT00000035120	FAM19A4	-0,65386	-4,23685	0,000386	0,002243	-0,21647
ENSGALT00000042245	gga-mir-1695	-0,60915	-4,23654	0,000386	0,002243	-0,2172
ENSGALT00000037596	RIN3	0,601433	4,232364	0,00039	0,002263	-0,2269
ENSGALT00000042252	SNORA26	0,961615	4,229931	0,000392	0,002272	-0,23255
NM_205308	FABP7	0,705019	4,229742	0,000393	0,002272	-0,23299
ENSGALT00000042514	5S	-0,59782	-4,2293	0,000393	0,002272	-0,23402
NM_204765	MEOX1	0,595632	4,228021	0,000394	0,002276	-0,23698
ENSGALT00000037506	Q5F396	-0,70946	-4,22574	0,000396	0,00228	-0,24229
ENSGALT00000005630	C5orf33	-0,66892	-4,22312	0,000399	0,002292	-0,24838
ENSGALT00000016784	PLCH1	0,693487	4,220774	0,000401	0,0023	-0,25382
ENSGALT00000025244	GDAP1	-0,51278	-4,21989	0,000402	0,002302	-0,25588
ENSGALT00000011594	FAM46D	-0,43218	-4,21689	0,000405	0,002315	-0,26283
NM_205427	IFNA3	-1,06925	-4,21657	0,000405	0,002315	-0,26359
ENSGALT00000011449	OTOA	-0,53294	-4,21307	0,000408	0,002329	-0,27172
ENSGALT00000028993	gga-mir-32	0,884589	4,211776	0,00041	0,002333	-0,27472
ENSGALT00000016238	C1orf84	-0,89241	-4,2054	0,000416	0,002363	-0,28952
ENSGALT00000005650	FNDC5	-0,76142	-4,20494	0,000416	0,002363	-0,29059
ENSGALT00000042242	5S	-0,73791	-4,20118	0,00042	0,002382	-0,29931
NM_204235	BIRC5	0,480097	4,199841	0,000421	0,002385	-0,30243
NM_205352	TAL1	0,971591	4,199719	0,000421	0,002385	-0,30272
ENSGALT00000014916	ECT2	0,502073	4,196587	0,000425	0,0024	-0,30999

ENSGALT00000039811	HSPB7	1,453616	4,19243	0,000429	0,002418	-0,31964
ENSGALT00000014630	MAMDC4	0,602395	4,192383	0,000429	0,002418	-0,31975
ENSGALT00000042130	gga-mir-1697	-0,71606	-4,19137	0,00043	0,002422	-0,3221
ENSGALT00000018347	LOC424199	0,672597	4,188237	0,000433	0,002437	-0,32938
ENSGALT00000036591	LYG2	0,836336	4,182224	0,000439	0,002469	-0,34334
NM_001098608	OSTN	1,5673	4,176223	0,000446	0,002497	-0,35727
ENSGALT00000040621	PRPF3	-0,60828	-4,17615	0,000446	0,002497	-0,35744
ENSGALT00000042469	SNORD88	-0,74653	-4,17504	0,000447	0,0025	-0,36003
NM_001005816	GABPB2	0,351211	4,172102	0,00045	0,002515	-0,36684
ENSGALT00000027562	NBEA	-0,47255	-4,16884	0,000453	0,002529	-0,37442
ENSGALT00000008955	TMEM56	0,75232	4,166511	0,000456	0,00254	-0,37981
NM_204442	F7	0,77463	4,165341	0,000457	0,002544	-0,38253
ENSGALT00000015621	LOC423943	0,753138	4,156073	0,000467	0,002598	-0,40404
ENSGALT00000010930	Q5W4T2	-0,7157	-4,15417	0,000469	0,002607	-0,40846
ENSGALT00000036429	Q90858	-0,45212	-4,1535	0,00047	0,002608	-0,41001
ENSGALT00000038651	CRBB3	0,762953	4,152077	0,000472	0,002614	-0,41331
ENSGALT00000020078	BAIAP2L2	0,472286	4,151534	0,000472	0,002615	-0,41457
ENSGALT00000015731	LOC771944	-0,69513	-4,14997	0,000474	0,002621	-0,41821
ENSGALT00000028792		-0,75875	-4,14645	0,000478	0,002641	-0,42636
ENSGALT00000005752	LOC427440	-0,37018	-4,14105	0,000484	0,002672	-0,43889
ENSGALT00000015307	LRRIQ4	0,787456	4,13964	0,000486	0,002678	-0,44217
ENSGALT00000023410	GNB3	-0,67708	-4,13776	0,000488	0,002687	-0,44653
ENSGALT00000009259	IFNA3	-1,03839	-4,13619	0,00049	0,002694	-0,45017
ENSGALT00000025240	LOC418465	-0,77784	-4,13308	0,000493	0,002705	-0,45737
ENSGALT00000024082	ENC1	-0,55571	-4,13306	0,000493	0,002705	-0,45742
ENSGALT00000016800	SYT1	-0,6026	-4,12903	0,000498	0,002724	-0,46677
ENSGALT00000016530	TCTE1	0,7655	4,128798	0,000498	0,002724	-0,46732
ENSGALT00000009152	HSCB	-1,31329	-4,12337	0,000505	0,002756	-0,47991
ENSGALT00000038850	CPNE7	0,680259	4,117047	0,000512	0,002792	-0,49457
NM_001029849	RHOC	0,369472	4,116954	0,000513	0,002792	-0,49479
ENSGALT00000014471	Q6R6I2	-0,49921	-4,11584	0,000514	0,002793	-0,49737
ENSGALT00000009168	PKD1	-0,6193	-4,11399	0,000516	0,0028	-0,50165
ENSGALT00000029046	gga-mir-31	-0,68337	-4,1139	0,000516	0,0028	-0,50188
ENSGALT00000030522	LOC418222	-0,5516	-4,1081	0,000523	0,002835	-0,51531
ENSGALT00000017778	SPARCL1	-0,5847	-4,10785	0,000524	0,002835	-0,51589
NM_001113761	B3GNT5	-0,7805	-4,10736	0,000524	0,002835	-0,51703
NM_204394	GHSR	0,532281	4,103364	0,000529	0,002858	-0,5263
ENSGALT00000028946	gga-mir-33-1	-0,50844	-4,103	0,00053	0,002858	-0,52714
ENSGALT00000023647	RHOBTB3	-0,4388	-4,10193	0,000531	0,002862	-0,52962
NM_001038586	GLULD1	0,638223	4,09465	0,00054	0,002907	-0,5465
ENSGALT00000030772	MRPL13	-0,66845	-4,09455	0,000541	0,002907	-0,54673
ENSGALT00000013846	ACZ	-0,42836	-4,09382	0,000541	0,002909	-0,54842
ENSGALT00000007035	LUZP2	0,6443	4,092824	0,000543	0,002912	-0,55073
ENSGALT00000009435	MCM10	0,483821	4,09128	0,000545	0,00292	-0,55431
ENSGALT00000021862	C9orf98	0,612973	4,090469	0,000546	0,002922	-0,55619
ENSGALT00000025645	CD109	-0,60467	-4,09014	0,000546	0,002922	-0,55695
ENSGALT00000039141	COHA1	0,901659	4,087616	0,000549	0,002936	-0,5628
ENSGALT00000008702	FRRS1	0,557379	4,084307	0,000554	0,002953	-0,57047
ENSGALT00000025078	SORCS2	-0,85147	-4,07873	0,000561	0,002986	-0,58339
ENSGALT00000006516	Q8AYE5	0,934568	4,069444	0,000574	0,003045	-0,60491

ENSGALT00000030302	SMPX	0,864335	4,069126	0,000574	0,003045	-0,60565
ENSGALT00000013920	Q9YH84	-0,45486	-4,06834	0,000575	0,003047	-0,60747
ENSGALT00000013712	LARG2	0,358936	4,061928	0,000584	0,003086	-0,62232
ENSGALT00000029745	LOC427060	0,311342	4,061683	0,000584	0,003086	-0,62289
ENSGALT00000036419	ANGPTL5	0,799853	4,058276	0,000589	0,003108	-0,63078
ENSGALT00000018829	BCL2L14	0,690099	4,057715	0,00059	0,003109	-0,63208
ENSGALT00000040915	LOC770241	0,396836	4,05638	0,000592	0,003115	-0,63517
NM_001012318	BIRC5	0,514766	4,055533	0,000593	0,003118	-0,63713
ENSGALT00000027248	NALCN	-0,43927	-4,05375	0,000595	0,003128	-0,64126
ENSGALT00000014055	TTBK1	-0,6612	-4,05168	0,000598	0,003138	-0,64605
ENSGALT00000005314	Q2YHU3	0,742571	4,051266	0,000599	0,003138	-0,64702
ENSGALT00000018072	AK7	0,738435	4,051221	0,000599	0,003138	-0,64712
ENSGALT00000029399	Q5MB12	-0,79093	-4,04552	0,000607	0,00317	-0,66031
ENSGALT00000042448	gga-mir-1554	-0,55293	-4,0455	0,000607	0,00317	-0,66037
ENSGALT00000008577	MSLN	-0,59312	-4,04324	0,00061	0,003181	-0,66561
NM_204956	SERGEF	0,637693	4,04091	0,000614	0,003195	-0,67099
ENSGALT00000016809	FLVCR2	-0,83341	-4,04019	0,000615	0,003197	-0,67267
NM_001001296	ISG12-2	1,321229	4,038323	0,000617	0,003208	-0,67698
NM_001030625	CCDC5	0,490003	4,037831	0,000618	0,003209	-0,67813
NM_001012319	BIRC5	0,492204	4,03666	0,00062	0,003214	-0,68084
ENSGALT00000027292	RAP2A	-0,58268	-4,02884	0,000631	0,003268	-0,69894
NM_001137648	SOCS1	0,417642	4,024258	0,000638	0,0033	-0,70954
ENSGALT00000019606	NDST4	-0,72126	-4,02374	0,000639	0,003301	-0,71074
ENSGALT00000014340	IQUB	0,52806	4,022988	0,00064	0,003303	-0,71248
ENSGALT00000039534	CDHR3	-0,74697	-4,02209	0,000642	0,003307	-0,71456
ENSGALT00000015254	ACCN5	-0,4656	-4,02026	0,000644	0,003317	-0,71879
ENSGALT00000033662	USP31	-0,86625	-4,01983	0,000645	0,003317	-0,7198
ENSGALT00000007470	PKN3	0,398325	4,019562	0,000645	0,003317	-0,72041
ENSGALT00000042614	SNORD5	-0,9452	-4,01905	0,000646	0,003317	-0,72159
NM_204577	ALDH1A1	0,626043	4,01848	0,000647	0,003317	-0,72291
ENSGALT00000023425	CDCA3	0,412788	4,017733	0,000648	0,003317	-0,72464
ENSGALT00000014481	PLCB4	-0,42005	-4,01732	0,000649	0,003317	-0,72559
ENSGALT00000025678	LOC421866	-0,78818	-4,01498	0,000652	0,003333	-0,731
ENSGALT00000023981	LOC768482	-0,47554	-4,01219	0,000657	0,003349	-0,73746
ENSGALT00000021177	CDYL2	-0,57533	-4,01209	0,000657	0,003349	-0,73769
NM_001007087	BLM	-0,43489	-4,01139	0,000658	0,003351	-0,73933
NM_001128062	GALR1	-0,56017	-4,00681	0,000665	0,003384	-0,7499
NM_204207	HAT1	0,365267	4,003285	0,000671	0,003406	-0,75807
ENSGALT00000042406	SNORD111	-0,66503	-3,99916	0,000677	0,003435	-0,7676
ENSGALT00000007354	LOC428696	0,54675	3,998794	0,000678	0,003435	-0,76845
ENSGALT00000022099	C9orf19	0,664532	3,997659	0,00068	0,00344	-0,77108
ENSGALT00000018923	TMEM179	-0,68643	-3,9973	0,00068	0,00344	-0,77191
ENSGALT00000017392	CKS2	0,556216	3,992854	0,000687	0,003473	-0,78219
ENSGALT00000030236	IL1R2	0,695946	3,991832	0,000689	0,003478	-0,78455
ENSGALT00000027031	ATP10A	0,461396	3,989937	0,000692	0,003489	-0,78893
ENSGALT00000005631	C5orf33	-0,71422	-3,98974	0,000693	0,003489	-0,78939
ENSGALT00000040890	LOC770562	0,349057	3,986586	0,000698	0,003511	-0,79668
ENSGALT00000042215	SNORD71	-0,57169	-3,98526	0,0007	0,003519	-0,79974
ENSGALT00000027775	C11orf70	0,524444	3,984422	0,000701	0,003522	-0,80169
ENSGALT00000037946	DGKB	-0,50781	-3,98107	0,000707	0,003547	-0,80942

ENSGALT00000019774	D2CP28	0,429855	3,979488	0,000709	0,003556	-0,81309
ENSGALT00000036881	Q5ZL49	0,374907	3,975782	0,000716	0,003584	-0,82166
ENSGALT00000027747	Q90858	-0,4757	-3,97501	0,000717	0,003587	-0,82345
NM_204696	FGF10	0,590104	3,973352	0,00072	0,003598	-0,82727
ENSGALT00000022382	CADH7	-0,61375	-3,97265	0,000721	0,0036	-0,8289
ENSGALT00000011460	ATP13A3	-0,50023	-3,96952	0,000726	0,003623	-0,83614
NM_001159371	HYDIN	0,942167	3,967943	0,000729	0,003631	-0,83977
ENSGALT00000024217	SLC22A15	-0,51703	-3,96784	0,000729	0,003631	-0,84001
ENSGALT00000037582	LOC427369	-0,6763	-3,96745	0,00073	0,003631	-0,84092
ENSGALT00000036393	GAB2	-0,93929	-3,96335	0,000737	0,003662	-0,8504
ENSGALT00000035152	C1orf70	-0,51394	-3,96053	0,000742	0,003683	-0,8569
ENSGALT00000038397	PUNC	0,66416	3,958088	0,000746	0,003701	-0,86254
ENSGALT00000025118	LOC768418	-0,4628	-3,95468	0,000752	0,003727	-0,87042
ENSGALT00000015369	GPR85	-0,45315	-3,95391	0,000754	0,00373	-0,87219
ENSGALT00000008364	Q2MCJ7	0,686987	3,950589	0,00076	0,003756	-0,87986
ENSGALT00000027415	EPSTI1	-0,60637	-3,94763	0,000765	0,003768	-0,88671
ENSGALT00000015742	LOC771944	-0,6401	-3,93785	0,000783	0,003848	-0,90928
ENSGALT00000033698	LOC424428	-0,71238	-3,93573	0,000787	0,00386	-0,91418
ENSGALT00000021976		-0,62386	-3,93375	0,00079	0,003873	-0,91875
NM_001031257	INHA	0,76805	3,931969	0,000794	0,003884	-0,92286
ENSGALT00000021588	MURC	-0,37183	-3,93148	0,000795	0,003884	-0,92398
ENSGALT00000009885	MYOT	0,510381	3,927993	0,000801	0,003912	-0,93204
ENSGALT00000026597	CB043	-0,49172	-3,92768	0,000802	0,003912	-0,93275
ENSGALT00000004498	PDZD2	-0,68252	-3,92599	0,000805	0,003924	-0,93665
ENSGALT00000041064	ETS1A	-0,61299	-3,92525	0,000806	0,003927	-0,93836
ENSGALT00000039020	LOC425462	0,537173	3,923332	0,00081	0,003941	-0,94279
ENSGALT00000013548	COHA1	0,8276	3,91676	0,000823	0,003999	-0,95795
ENSGALT00000015769	PON2	0,692217	3,916387	0,000824	0,003999	-0,95881
ENSGALT00000037787	Q6V0P3	-0,59342	-3,91542	0,000825	0,004004	-0,96104
ENSGALT00000035363	LOC769570	-0,62755	-3,91421	0,000828	0,004011	-0,96383
ENSGALT00000004839	TADA1L	0,385241	3,913354	0,000829	0,004016	-0,96581
NM_204189	UNG	0,402437	3,910666	0,000835	0,004038	-0,97201
ENSGALT00000022516	Q5ZJR6	-0,41208	-3,90894	0,000838	0,00405	-0,976
ENSGALT00000024645	NUDT12	-0,49349	-3,9043	0,000847	0,004087	-0,9867
ENSGALT00000015542	ZFYVE26	-0,80197	-3,90426	0,000847	0,004087	-0,9868
NM_001024834	SSTR5	-0,5885	-3,89964	0,000857	0,004121	-0,99744
ENSGALT00000041420	gga-mir-22	0,525982	3,899607	0,000857	0,004121	-0,99752
NM_001006139	MCM2	0,405434	3,898054	0,00086	0,004132	-1,0011
ENSGALT00000038942	Q6B842	-0,68379	-3,89687	0,000862	0,004137	-1,00384
ENSGALT00000040884	LOC770639	0,354776	3,896774	0,000863	0,004137	-1,00405
ENSGALT00000042609	U1	-0,72786	-3,8958	0,000865	0,004143	-1,00631
ENSGALT00000042608	SCARNA13	-0,51636	-3,89466	0,000867	0,004149	-1,00892
ENSGALT00000013098	LOC429152	-0,46655	-3,89438	0,000867	0,004149	-1,00956
ENSGALT00000042556	5S	-0,7903	-3,89349	0,000869	0,004153	-1,01161
NM_001034818	LIN28B	-0,54837	-3,89002	0,000876	0,004182	-1,01962
NM_204697	POPDC3	0,789543	3,88983	0,000877	0,004182	-1,02006
ENSGALT00000040615	CHD6	-0,7425	-3,88823	0,00088	0,004193	-1,02374
NM_001030619	RPS14	-0,69884	-3,88789	0,000881	0,004193	-1,02453
NM_001001302	MYH7	-0,63671	-3,8858	0,000885	0,00421	-1,02934
ENSGALT00000019370	Q8AY19	-0,51591	-3,88468	0,000888	0,004217	-1,03192

ENSGALT00000011866	LOC415529	0,767605	3,881888	0,000893	0,004238	-1,03836
ENSGALT00000006733	PRRX2	0,695263	3,881803	0,000894	0,004238	-1,03856
ENSGALT00000022124	SCGN	0,638205	3,881123	0,000895	0,004241	-1,04012
ENSGALT00000005408	PTPRT	-0,65782	-3,8781	0,000901	0,004267	-1,0471
ENSGALT00000027027	GABRA5	-0,65327	-3,8775	0,000903	0,004267	-1,04848
NM_001001760	CDH13	-0,67097	-3,87731	0,000903	0,004267	-1,04892
ENSGALT00000036940	B2ZE94	-0,62224	-3,87621	0,000905	0,004274	-1,05144
ENSGALT00000009711	IL13RA1	-0,81797	-3,87444	0,000909	0,004288	-1,05553
ENSGALT00000038040	MYSM1	-0,41594	-3,87364	0,000911	0,004292	-1,05735
ENSGALT00000025803	SLC4A11	-0,88489	-3,86782	0,000924	0,004344	-1,07078
NM_001037269	WNT5B	0,420721	3,863159	0,000934	0,004388	-1,08151
ENSGALT00000027726	RAB39	-0,37631	-3,86075	0,000939	0,004409	-1,08706
ENSGALT00000024694	LNPEP	-0,74812	-3,8551	0,000952	0,004464	-1,10006
ENSGALT00000037087	COCA1	-0,56023	-3,85447	0,000953	0,004466	-1,1015
ENSGALT00000025981	B3GALT5	-0,6902	-3,85354	0,000955	0,004469	-1,10365
ENSGALT00000011882	TMEM114	0,557423	3,850648	0,000962	0,004491	-1,11031
ENSGALT00000006402	PQLC2	0,363812	3,850498	0,000962	0,004491	-1,11065
ENSGALT00000010396	Q49L21	0,735016	3,850203	0,000963	0,004491	-1,11133
ENSGALT00000007940	NTCP7	-0,55751	-3,84693	0,00097	0,004517	-1,11888
ENSGALT00000036793	ASXL2	-0,61079	-3,84605	0,000972	0,004521	-1,1209
ENSGALT00000021873	LOC426093	0,859347	3,845811	0,000973	0,004521	-1,12144
ENSGALT00000006435	NBL1	0,408348	3,84508	0,000975	0,004525	-1,12312
ENSGALT00000038887	RBM46	0,464437	3,843201	0,000979	0,004541	-1,12745
ENSGALT00000022286	SOCS7	-0,52809	-3,83421	0,001	0,004634	-1,14814
ENSGALT00000022384	CDT1	0,350078	3,832004	0,001005	0,004654	-1,15321
ENSGALT00000040081	CAC1B	-0,52002	-3,82905	0,001012	0,004678	-1,16
ENSGALT00000041081	Q90591	-1,04359	-3,82904	0,001012	0,004678	-1,16002
ENSGALT00000021833	LOC430516	0,418726	3,827402	0,001016	0,004688	-1,16379
ENSGALT00000005259	KIAA1529	0,79301	3,827214	0,001016	0,004688	-1,16422
NM_001081504	NRTN	0,522936	3,826998	0,001017	0,004688	-1,16472
ENSGALT00000022435	HECA	-0,54178	-3,82576	0,00102	0,004697	-1,16756
ENSGALT00000032278		0,564613	3,82283	0,001027	0,004721	-1,17431
ENSGALT00000005607	C9orf171	1,198784	3,820875	0,001032	0,004739	-1,1788
ENSGALT00000011695	A5JNH0	1,727393	3,815948	0,001044	0,004785	-1,19013
ENSGALT00000021056	SYT10	-0,61653	-3,81447	0,001047	0,004798	-1,19353
ENSGALT00000009201	Q9PVP9	0,425899	3,813907	0,001049	0,0048	-1,19482
NM_001195157	LOC416618	0,723103	3,811741	0,001054	0,00482	-1,1998
NM_001167752	MB	0,63051	3,807597	0,001065	0,004859	-1,20932
ENSGALT00000040413	LMF2	-0,63235	-3,8067	0,001067	0,004865	-1,21138
ENSGALT00000018176	ALX1	0,479058	3,805689	0,001069	0,004872	-1,2137
NM_001001301	TPH2	0,401864	3,801167	0,001081	0,00492	-1,22409
ENSGALT00000036713	AMP1	1,019464	3,800501	0,001083	0,004923	-1,22562
ENSGALT00000036271	PTPDC1	-0,33894	-3,79657	0,001093	0,004965	-1,23465
ENSGALT00000019895	PLA2G12A	0,574217	3,795333	0,001096	0,004975	-1,23749
NM_205344	HMOX1	0,487407	3,792566	0,001103	0,005003	-1,24385
ENSGALT00000018581	AMN	1,056532	3,787058	0,001117	0,005064	-1,25649
ENSGALT00000014964	Q7ZT14	0,534287	3,785137	0,001122	0,005082	-1,2609
ENSGALT00000026428	MYT1L	-0,46909	-3,7827	0,001129	0,005099	-1,2665
ENSGALT00000037129	LOC771856	0,501873	3,782589	0,001129	0,005099	-1,26675
NM_204120	EDNRB2	0,504818	3,782008	0,001131	0,005102	-1,26809

ENSGALT00000042502	U6	-0,52086	-3,7815	0,001132	0,005103	-1,26926
ENSGALT00000012774	SLC9A5	0,427242	3,780626	0,001134	0,005109	-1,27126
NM_001006274	CKAP2	0,461166	3,779083	0,001139	0,005123	-1,2748
ENSGALT00000033175	KLF1	0,622688	3,776831	0,001145	0,005146	-1,27997
ENSGALT00000028574	SNORA74	-0,50891	-3,77588	0,001147	0,005153	-1,28216
ENSGALT00000027911	GAB2	-0,67435	-3,77511	0,001149	0,005153	-1,28391
ENSGALT00000024613	ST18	-0,4256	-3,77461	0,001151	0,005155	-1,28506
ENSGALT00000012536	RTN4R	0,59865	3,768419	0,001168	0,005223	-1,29927
NM_001001741	CCK	0,578556	3,765704	0,001175	0,00525	-1,3055
ENSGALT00000036766	Q9I9L4	-0,51988	-3,7651	0,001177	0,00525	-1,30688
NM_205389	DNTT	-1,10842	-3,765	0,001177	0,00525	-1,3071
ENSGALT00000020225	NUP43	0,337157	3,761841	0,001186	0,005284	-1,31436
ENSGALT00000032465	LOC776219	0,441295	3,761245	0,001187	0,005287	-1,31572
ENSGALT00000026574	KCNS3	0,524841	3,760813	0,001189	0,005288	-1,31671
ENSGALT00000030593	MOT3	0,417286	3,759533	0,001192	0,005299	-1,31965
ENSGALT00000015305	Q9PUI8	-0,45424	-3,75718	0,001199	0,005319	-1,32505
ENSGALT00000020409	Q9DEH4	-1,02084	-3,75688	0,0012	0,005319	-1,32574
ENSGALT00000015954	MAML3	-0,70569	-3,75567	0,001203	0,005321	-1,32851
ENSGALT00000020982	SERPINB2	1,108589	3,754094	0,001208	0,005335	-1,33212
ENSGALT00000014689	BTBD3	-0,534	-3,75308	0,001211	0,005343	-1,33445
ENSGALT00000007291	MLRA	0,59947	3,752468	0,001212	0,005346	-1,33585
NR_035053	MIR1569	-0,58926	-3,75215	0,001213	0,005346	-1,33657
NM_204981	WNT9A	0,689058	3,749014	0,001222	0,005376	-1,34377
ENSGALT00000036717	GLL7	1,441353	3,747487	0,001227	0,005391	-1,34726
ENSGALT00000018294	LOC425670	-0,43103	-3,7465	0,001229	0,005398	-1,34952
ENSGALT00000004149	LOC772195	-0,57979	-3,74455	0,001235	0,005419	-1,35398
ENSGALT00000038498	Q6PKI7	0,445805	3,739856	0,001249	0,005471	-1,36475
ENSGALT00000018715		-0,39922	-3,73951	0,00125	0,005471	-1,36554
NM_001030008	AP1S3	-0,60506	-3,73934	0,00125	0,005471	-1,36594
ENSGALT00000037912	CHSY3	0,550328	3,738236	0,001254	0,005476	-1,36846
ENSGALT00000016923	KIAA1737	-0,60086	-3,735	0,001263	0,005513	-1,37588
ENSGALT00000016623	LOC423347	0,35383	3,734374	0,001265	0,005517	-1,37731
ENSGALT00000026298	C6orf142	0,836533	3,733698	0,001267	0,005521	-1,37886
ENSGALT00000017179	CYP1B1	-1,25904	-3,73198	0,001272	0,005539	-1,3828
ENSGALT00000025892	CTHRC1	0,734794	3,730999	0,001275	0,005543	-1,38504
ENSGALT00000036421	KIAA1377	0,651125	3,730945	0,001275	0,005543	-1,38516
ENSGALT00000036384	NARE	0,830886	3,729852	0,001279	0,005552	-1,38766
ENSGALT00000041325	Q5ZK97	0,625893	3,728393	0,001283	0,005566	-1,391
NM_204377	CSNK1E	0,525895	3,724932	0,001293	0,005607	-1,39893
ENSGALT00000038475	RDH5	-0,52199	-3,72426	0,001296	0,005611	-1,40046
ENSGALT00000037701	KCC2D	-0,55472	-3,72165	0,001304	0,005641	-1,40645
NM_001001193	GAL6	1,099055	3,71491	0,001324	0,005722	-1,42187
ENSGALT00000007336	Q5F3Z8	-0,57147	-3,70926	0,001342	0,005793	-1,43479
ENSGALT00000009353	KCNQ2	-0,56756	-3,70701	0,001349	0,005819	-1,43995
ENSGALT00000017884	B1B565	-0,49977	-3,70553	0,001354	0,005834	-1,44334
ENSGALT00000013701	KIAA1754	-0,5763	-3,70222	0,001364	0,005875	-1,4509
NM_204638	FSTL1	-0,60308	-3,70086	0,001369	0,005889	-1,45401
ENSGALT00000036383	DCHS1	-0,68421	-3,70025	0,001371	0,005891	-1,45539
ENSGALT00000039097	Q98909	-0,62593	-3,69932	0,001374	0,005891	-1,45752
ENSGALT00000037508	CCDC112	-0,39802	-3,699	0,001375	0,005891	-1,45826

ENSGALT00000025178	KISHA	0,404382	3,698928	0,001375	0,005891	-1,45843
ENSGALT00000015350	KIAA0247	-0,60781	-3,6989	0,001375	0,005891	-1,4585
ENSGALT00000037768	Q8AY19	-0,48453	-3,69517	0,001387	0,005938	-1,46701
ENSGALT00000004960	Q5D7U5	0,551521	3,693405	0,001393	0,005958	-1,47105
NM_204838	DUSP4	-0,37738	-3,69275	0,001395	0,005961	-1,47255
NM_205009	KRT19	1,881439	3,692442	0,001396	0,005961	-1,47325
ENSGALT00000023814	TRIM23	-0,65708	-3,69137	0,0014	0,005971	-1,47571
ENSGALT00000037611	SMC2	0,352081	3,688817	0,001408	0,006002	-1,48154
ENSGALT00000015077	FIBA	1,147064	3,688006	0,001411	0,006003	-1,48339
ENSGALT00000037199	A2M	0,757493	3,687837	0,001411	0,006003	-1,48378
NM_204625	LOC395334	-0,97125	-3,68751	0,001413	0,006003	-1,48453
NM_001001772	SLC24A2	-0,4092	-3,68735	0,001413	0,006003	-1,48489
ENSGALT00000040409	LUZP2	0,594222	3,686405	0,001416	0,006008	-1,48705
ENSGALT00000019259	ESR2	0,679583	3,682808	0,001428	0,006048	-1,49527
ENSGALT00000033738	LOC429068	0,563894	3,682729	0,001428	0,006048	-1,49545
ENSGALT00000010826	LOC416599	-0,40623	-3,68036	0,001436	0,006075	-1,50087
ENSGALT00000038461	O42419	-0,45568	-3,68015	0,001437	0,006075	-1,50135
ENSGALT00000006729	PTGES	0,486492	3,677547	0,001446	0,006107	-1,50728
NM_001031005	RPRD1A	-0,46401	-3,6747	0,001456	0,006143	-1,51378
ENSGALT00000017469	TMEM48	0,323047	3,673045	0,001461	0,006162	-1,51757
ENSGALT00000037167	CAD20	0,612345	3,670491	0,00147	0,006194	-1,5234
ENSGALT00000040257	PATL2	0,619941	3,669656	0,001473	0,0062	-1,5253
ENSGALT00000016467	CSGALNACT1	-0,51011	-3,66902	0,001475	0,0062	-1,52676
NM_001080716	ROR2	-0,59698	-3,66901	0,001475	0,0062	-1,52678
ENSGALT00000029703	TBCEL	-0,63649	-3,65878	0,001511	0,006337	-1,55013
ENSGALT00000040087	UNC80	-0,44933	-3,65869	0,001512	0,006337	-1,55033
ENSGALT00000019424	KIF15	0,348594	3,653369	0,001531	0,006406	-1,56247
ENSGALT00000010578	Q805A7	-0,50056	-3,65303	0,001532	0,006406	-1,56326
ENSGALT00000025117	LOC427315	-0,36081	-3,65097	0,001539	0,006432	-1,56794
ENSGALT00000037577	SERPINA1	1,080231	3,650247	0,001542	0,006437	-1,56959
ENSGALT00000038440	MYPT1	-0,62146	-3,64719	0,001553	0,006478	-1,57657
ENSGALT00000017570	RIN3	0,567126	3,645556	0,001559	0,006498	-1,58029
ENSGALT00000033798	Q646T7	-0,49253	-3,64452	0,001563	0,006509	-1,58266
ENSGALT00000032841	TMEM171	-0,4121	-3,64041	0,001578	0,006566	-1,59203
ENSGALT00000011745	Q90591	-1,03192	-3,63966	0,001581	0,006572	-1,59373
ENSGALT00000032816	C9orf95	0,348351	3,637728	0,001588	0,006597	-1,59813
ENSGALT00000032927	TCTEX1D1	0,484214	3,635278	0,001597	0,00663	-1,60372
ENSGALT00000042247	gga-mir-1689	-0,57464	-3,6345	0,0016	0,006633	-1,6055
ENSGALT00000036628	NHS	-0,48104	-3,63436	0,0016	0,006633	-1,6058
NM_001113289	MDK	0,744811	3,631917	0,00161	0,006666	-1,61138
ENSGALT00000007192	RAD51	0,420357	3,625308	0,001635	0,006759	-1,62643
NM_204869	RBPMS2	0,413734	3,620863	0,001652	0,006813	-1,63655
ENSGALT00000020096	GRM1	-0,60706	-3,62036	0,001654	0,006816	-1,63769
ENSGALT00000027028	GBRB3	-0,42521	-3,61967	0,001657	0,006817	-1,63927
NM_001030767	CECR1	-0,63156	-3,61962	0,001657	0,006817	-1,63938
ENSGALT00000026001	RHPN1	-0,47955	-3,61767	0,001664	0,006842	-1,64382
NM_001145228	ADAM22	-0,3235	-3,61497	0,001675	0,00688	-1,64997
ENSGALT00000013899	PPP2R5D	-0,51574	-3,61352	0,001681	0,006898	-1,65326
ENSGALT00000042535	U6	-0,71137	-3,61184	0,001687	0,006917	-1,65709
ENSGALT00000016998	MKNK1	0,351401	3,611671	0,001688	0,006917	-1,65748

ENSGALT00000041252	A2PYM3	0,375896	3,610017	0,001694	0,006939	-1,66124
ENSGALT00000026238	TMEM47	-0,51626	-3,60601	0,00171	0,006998	-1,67036
NM_204117	NRG1	-0,25751	-3,60538	0,001713	0,006999	-1,67178
NR_035346	MIR3532	-0,65821	-3,60189	0,001727	0,007049	-1,67972
ENSGALT00000036296	ITIH3	0,781686	3,599688	0,001736	0,007076	-1,68474
NM_204670	RBBP7	0,377277	3,59963	0,001736	0,007076	-1,68487
ENSGALT00000009334	LOC427400	-0,78967	-3,59784	0,001744	0,007094	-1,68893
ENSGALT00000042246	gga-mir-1731	-0,65991	-3,59217	0,001767	0,007176	-1,70184
NM_001134359	FANCA	0,373287	3,592002	0,001768	0,007176	-1,70221
ENSGALT00000005551	C9orf9	0,632511	3,589134	0,00178	0,007212	-1,70873
NM_001031537	AREGB	0,50459	3,589093	0,00178	0,007212	-1,70882
ENSGALT00000020869	DSP	0,836121	3,581829	0,00181	0,00732	-1,72533
NM_001004404	LARGE	0,311972	3,581721	0,001811	0,00732	-1,72558
ENSGALT00000036284	LOC430723	-0,70815	-3,58134	0,001812	0,007321	-1,72644
ENSGALT00000040295	ACRC	0,443115	3,579847	0,001819	0,007341	-1,72983
NM_001024836	IFNB	-0,7248	-3,57591	0,001836	0,007403	-1,73877
ENSGALT00000038596	MKI67	0,419269	3,57527	0,001838	0,007408	-1,74023
ENSGALT00000032784	ATG10	-0,30736	-3,57273	0,001849	0,007446	-1,74599
ENSGALT00000016667	K2CO	1,119084	3,572441	0,001851	0,007446	-1,74665
NM_205219	E2F1	0,488811	3,571752	0,001854	0,007452	-1,74822
ENSGALT00000006429	DNA2L	0,375735	3,569222	0,001865	0,007491	-1,75396
NM_001031303	KLHL6	0,578382	3,568666	0,001867	0,007494	-1,75522
NM_204134	CDH20	0,593118	3,567823	0,001871	0,007503	-1,75713
NM_001044632	MYL1	1,059754	3,563716	0,001889	0,00757	-1,76646
ENSGALT00000037049	CO9A1	-0,58722	-3,56188	0,001897	0,007597	-1,77063
NM_205364	CHRNA6	-0,39428	-3,56072	0,001902	0,007611	-1,77324
NM_204327	TNFSF13B	0,75103	3,559208	0,001909	0,007632	-1,77668
ENSGALT00000018182	PLA2R1	-0,67314	-3,55857	0,001912	0,007637	-1,77813
ENSGALT00000036582	Q78AW4	0,410484	3,558268	0,001913	0,007637	-1,77882
ENSGALT00000010196	UROC1	0,389533	3,557901	0,001915	0,007638	-1,77965
NM_001037836	LY75	-0,48762	-3,557	0,001919	0,007644	-1,78168
NM_001039453	AQP1	0,677264	3,556891	0,001919	0,007644	-1,78194
NM_204365	SLC15A1	1,168635	3,555909	0,001924	0,007655	-1,78417
ENSGALT00000004315	LOC776972	-0,60344	-3,5549	0,001928	0,007668	-1,78646
ENSGALT00000036605	FZD6	-1,09784	-3,55356	0,001934	0,007686	-1,7895
ENSGALT00000025887	BAALC	-1,00737	-3,55146	0,001944	0,007711	-1,79424
ENSGALT00000024413	Q65YQ3	-0,50473	-3,54676	0,001965	0,007791	-1,80491
ENSGALT00000015549	LOC423277	0,952018	3,542696	0,001984	0,007859	-1,81412
NM_205305	COL9A3	0,377146	3,542124	0,001987	0,007864	-1,81542
NM_001044677	MIS12	0,453935	3,540501	0,001994	0,007887	-1,8191
ENSGALT00000008674	CACNA2D3	0,496747	3,536159	0,002015	0,007944	-1,82893
ENSGALT00000007245	ONECUT1	-0,50885	-3,5361	0,002015	0,007944	-1,82906
NR_035354	MIR3539	-0,62399	-3,53316	0,002029	0,007992	-1,83573
ENSGALT00000027416	EPSTI1	-0,59405	-3,53288	0,00203	0,007992	-1,83636
ENSGALT00000014770	GALNT14	0,733054	3,532322	0,002033	0,007996	-1,83762
ENSGALT00000007577	OBSCN	0,640219	3,530974	0,002039	0,008015	-1,84068
ENSGALT00000026748	C8orf80	0,906147	3,530605	0,002041	0,008016	-1,84151
NM_001170527	CRYGS	-0,60022	-3,52989	0,002044	0,008017	-1,84313
ENSGALT00000011349	PYROXD2	0,553595	3,528135	0,002053	0,008044	-1,8471
ENSGALT00000007861	LRRC48	0,477119	3,517826	0,002103	0,008234	-1,87044

ENSGALT00000007858	LOC415780	-0,31806	-3,51548	0,002114	0,008273	-1,87574
ENSGALT00000037370	Q9W6V3	-0,36452	-3,51489	0,002117	0,008278	-1,87708
NM_001006485	VRK1	0,302243	3,512652	0,002129	0,008309	-1,88214
NM_205410	PLN	-0,53084	-3,51095	0,002137	0,00833	-1,88599
ENSGALT00000018041	C14orf49	-0,54609	-3,51092	0,002137	0,00833	-1,88606
ENSGALT00000009246	IFNB	-0,64273	-3,50752	0,002154	0,008379	-1,89375
ENSGALT00000013622	MK	0,680582	3,507428	0,002155	0,008379	-1,89395
ENSGALT00000027766	MMP3	-0,60366	-3,50685	0,002158	0,008384	-1,89526
ENSGALT00000009229	LOC428705	0,639689	3,50572	0,002163	0,008399	-1,89781
ENSGALT00000011622	ATP13A5	-0,59175	-3,50422	0,002171	0,008423	-1,90121
ENSGALT00000019399	PRKCH	0,513343	3,500103	0,002192	0,008478	-1,91051
ENSGALT00000042111	gga-mir-1708	-0,60462	-3,49956	0,002195	0,008482	-1,91173
ENSGALT00000020411	Q9DEH4	-0,69746	-3,49622	0,002212	0,008542	-1,91928
ENSGALT00000020960	RAB19	0,439213	3,495208	0,002217	0,00855	-1,92157
ENSGALT00000005939	VPS37B	-0,63036	-3,49423	0,002222	0,008563	-1,92378
ENSGALT00000020693	RNF144B	-0,68932	-3,49126	0,002238	0,008616	-1,93049
ENSGALT00000042573	SNORD121A	-0,46293	-3,48951	0,002247	0,008645	-1,93445
NM_206904	CD3E	0,702005	3,488429	0,002252	0,008653	-1,93688
ENSGALT00000004429	C2orf21	-0,69683	-3,48823	0,002254	0,008653	-1,93734
ENSGALT00000007696	Q5ZJQ1	0,321101	3,488081	0,002254	0,008653	-1,93767
ENSGALT00000015877	SLC7A11	0,806449	3,487788	0,002256	0,008653	-1,93832
ENSGALT00000042182	gga-mir-1635	-0,50924	-3,48742	0,002258	0,008654	-1,93916
ENSGALT00000010014	LOC416032	0,565644	3,484373	0,002274	0,00871	-1,94603
NM_204437	TPX2	0,349375	3,483285	0,00228	0,008725	-1,94849
ENSGALT00000015260	Q9PVI7	-0,3569	-3,48033	0,002295	0,008779	-1,95515
NM_204810	LECT1	0,612585	3,479758	0,002299	0,008784	-1,95645
ENSGALT00000040888	LOC770889	0,363243	3,477118	0,002313	0,008832	-1,96241
NM_205011	HPGDS	-0,58617	-3,47215	0,00234	0,008915	-1,97361
ENSGALT00000026294	FAM83B	0,699757	3,466222	0,002372	0,009032	-1,98698
NM_204682	SIK1	0,46669	3,464444	0,002382	0,009063	-1,99099
ENSGALT00000022456	NHSL1	-0,39226	-3,46086	0,002402	0,009132	-1,99907
ENSGALT00000026767	PBK	0,406558	3,457815	0,002419	0,009191	-2,00593
NM_204932	LOC395772	1,119447	3,455497	0,002432	0,009234	-2,01115
NM_204292	WNT7A	0,590633	3,453806	0,002442	0,009263	-2,01496
ENSGALT00000008709	PDLIM1	0,394039	3,449769	0,002465	0,009344	-2,02405
ENSGALT00000012422	Q5F3Q4	0,408356	3,447723	0,002477	0,009382	-2,02866
ENSGALT00000040612	Q8AYE5	0,763075	3,446622	0,002483	0,009399	-2,03114
ENSGALT00000015431	SAMD9L	0,873018	3,444985	0,002493	0,009428	-2,03482
ENSGALT00000040686	O93526	0,568381	3,44176	0,002512	0,009492	-2,04208
ENSGALT00000026448	CMPK2	0,577524	3,440838	0,002517	0,009498	-2,04415
ENSGALT00000012122	DENND4A	-0,48008	-3,43827	0,002532	0,009545	-2,04993
ENSGALT00000018652	Q800D9	-0,61933	-3,43808	0,002533	0,009545	-2,05035
ENSGALT00000006491	TAOK1	-0,54521	-3,43454	0,002554	0,009612	-2,05833
ENSGALT00000037655	A1IIE6	-0,59148	-3,43444	0,002555	0,009612	-2,05855
ENSGALT00000028099	RXFP3	-0,44269	-3,43319	0,002562	0,009631	-2,06137
ENSGALT00000032735	BAAT	0,814199	3,432989	0,002564	0,009631	-2,06181
NM_001098852	PIWIL1	0,842904	3,431594	0,002572	0,009655	-2,06495
NM_001006209	KPNA2	0,335976	3,431195	0,002574	0,009657	-2,06584
ENSGALT00000024678	LIX1	0,569846	3,429481	0,002585	0,009687	-2,0697
ENSGALT00000021261	CAC1C	-0,48811	-3,42662	0,002602	0,009732	-2,07612

NM_001048080	CD8A	0,555433	3,426193	0,002604	0,009734	-2,07709
ENSGALT00000030773	MRPL13	-0,57228	-3,42366	0,00262	0,009785	-2,08278
ENSGALT00000038217	PTAR1	-0,49974	-3,42226	0,002628	0,009809	-2,08594
ENSGALT00000016413	FAM160A1	-0,40169	-3,42174	0,002632	0,009811	-2,0871
ENSGALT00000040711	Q90WH5	-0,62403	-3,42155	0,002633	0,009811	-2,08753
ENSGALT00000037852		-0,55139	-3,42082	0,002637	0,009819	-2,08916
ENSGALT00000029980	SGK223	0,835138	3,42058	0,002639	0,009819	-2,0897
ENSGALT00000037079		0,853667	3,418167	0,002654	0,009867	-2,09513
ENSGALT00000025100	SH3TC1	0,793657	3,416942	0,002661	0,009888	-2,09788
ENSGALT00000041883	7SK	-0,7508	-3,41404	0,002679	0,009948	-2,10439
ENSGALT00000024818	ACHA6	-0,3993	-3,41321	0,002684	0,00996	-2,10627

***Supplementary Table S2** . The functional analysis on breed differentially expressed genes (Supplementary 1) showed a significant enrichment (FDR corrected P.Val < 0.001) in genes with function related to channel activity (GO: 0015267). The reported values include: transcription ID and annotation obtained from DAVID Bioinformatics Resources 6.7.*

**Transcript ID**	**Functional Annotation**
ENSGALT00000011338	5-hydroxytryptamine (serotonin) receptor 3A
ENSGALT00000041294	amiloride-sensitive cation channel 2, neuronal
ENSGALT00000015254	amiloride-sensitive cation channel 5, intestinal
ENSGALT00000024413	aquaporin 4
ENSGALT00000008674	calcium channel, voltage-dependent, alpha 2/delta 3 subunit
NM_204660	calcium channel, voltage-dependent, gamma subunit 3
ENSGALT00000013779	calcium channel, voltage-dependent, N type, alpha 1B subunit
ENSGALT00000010221	chloride channel, calcium activated, family member 2
ENSGALT00000031528	chloride intracellular channel 5
NM_205364	cholinergic receptor, nicotinic, alpha 6
ENSGALT00000027028	gamma-aminobutyric acid (GABA) A receptor, beta 3
ENSGALT00000037181	gamma-aminobutyric acid (GABA) receptor, rho 1
ENSGALT00000031682	gamma-aminobutyric acid (GABA) receptor, rho 2
ENSGALT00000027747	glutamate receptor, ionotrophic, AMPA 4
ENSGALT00000025530	glutamate receptor, ionotropic, kainate 1
NM_206979	glutamate receptor, ionotropic, N-methyl D-aspartate 1
NM_001030663	hydrogen voltage-gated channel 1
NM_205201	mature protein
ENSGALT00000020138	potassium channel tetramerisation domain containing 16
ENSGALT00000026103	potassium channel, subfamily K, member 9
ENSGALT00000034863	potassium channel, subfamily T, member 1
ENSGALT00000025794	potassium voltage-gated channel, Isk-related family
ENSGALT00000009353	potassium voltage-gated channel, KQT-like subfamily
NM_204906	potassium voltage-gated channel, shaker-related subfamily
ENSGALT00000016703	potassium voltage-gated channel, shaker-related subfamily
ENSGALT00000026539	potassium voltage-gated channel, subfamily F, member 1
ENSGALT00000019345	potassium voltage-gated channel, subfamily H (eag-related)
ENSGALT00000031464	potassium voltage-gated channel, subfamily H (eag-related)
ENSGALT00000018380	potassium voltage-gated channel, subfamily H (eag-related)
ENSGALT00000006151	purinergic receptor P2X, ligand-gated ion channel, 7
ENSGALT00000017582	ryanodine receptor 2 (cardiac)
ENSGALT00000023272	similar to Kv channel-interacting protein
ENSGALT00000038412	similar to Potassium voltage-gated channel, subfamily H
ENSGALT00000037166	sodium channel, nonvoltage-gated 1 alpha; similar to
ENSGALT00000017931	sodium channel, voltage-gated, type II, alpha subunit
ENSGALT00000011985	sodium channel, voltage-gated, type IV, beta
ENSGALT00000027248	sodium leak channel, non-selective
ENSGALT00000019370	transient receptor potential cation channel, subfamily C
ENSGALT00000012944	transient receptor potential cation channel, subfamily C
ENSGALT00000006114	transient receptor potential cation channel, subfamily M
ENSGALT00000024411	transient receptor potential cation channel, subfamily M

Supplementary Table S3. *The list of genes which are differently expressed genes in adrenals of the animals which were culled immediately after capture (baseline) and the animals which were retrained in a net for 15 minutes before the cull (stressed) based on the ANOVA model (~ treatment + breed + sex). The reported values include: transcription ID, gene, log2 fold change (logFC), P.Value, BH adjusted P.Value (adj.P.Val), and B Value. The positive logFC values suggest that the gene in up regulated in stressed and negative logFC values suggest up-regulation of the gene in baseline animals.*

Transcription ID	Gene	log fold cha	t	P.Val	adj.P.Val	B
ENSGALT00000022087	NR4A3	2,534328	14,50924	3,01E-12	1,54E-08	17,33555
ENSGALT00000040639	Q2UXM9	1,162005	10,82365	6,26E-10	1,6E-06	12,68977
ENSGALT00000015966	ATF3	1,361336	9,906182	2,91E-09	3,71E-06	11,28888
ENSGALT00000040996	STC2	0,988056	9,193661	1,03E-08	1,05E-05	10,12269
ENSGALT00000039200	PIM3	0,831873	8,601548	3,07E-08	2,24E-05	9,098725
NM_001045836	FOSL2	1,325667	8,394554	4,55E-08	2,9E-05	8,728675
ENSGALT00000004561	STC2	1,002549	8,236266	6,18E-08	3,5E-05	8,441413
ENSGALT00000011594	FAM46D	0,828027	8,079213	8,39E-08	4,1E-05	8,152703
ENSGALT00000038760	DFNB59	1,077745	8,052412	8,84E-08	4,1E-05	8,103068
NM_204849	HBEGF	0,850931	7,804187	1,44E-07	6,13E-05	7,638228
ENSGALT00000016303	FOSL2	1,310854	7,752569	1,6E-07	6,28E-05	7,540405
ENSGALT00000009704	AXUD1	0,814006	7,512294	2,6E-07	9,46E-05	7,079784
ENSGALT00000020478	NR4A2	1,043396	7,432169	3,06E-07	0,000104	6,924255
ENSGALT00000036355	KCNE3	1,02551	7,367727	3,49E-07	0,000111	6,79847
ENSGALT00000013972	DUSP5	0,835653	7,296637	4,04E-07	0,000121	6,658992
NM_001031098	POMC	0,9727	7,042819	6,85E-07	0,000194	6,154871
ENSGALT00000018629	DUS4	0,802356	6,886743	9,52E-07	0,000255	5,840168
ENSGALT00000011903	SST	1,178525	6,797142	1,15E-06	0,000279	5,657899
NM_001009928	CCRN4L	0,69071	6,531152	2,04E-06	0,000472	5,11004
ENSGALT00000015791	KLHL23	-0,82339	-6,43725	2,5E-06	0,000532	4,914249
ENSGALT00000018061	C1orf96	0,70423	6,436124	2,5E-06	0,000532	4,911898
NM_001037835	TLR15	0,982386	6,186424	4,33E-06	0,000848	4,385384
ENSGALT00000012442	FAM19A4	0,917208	6,048992	5,87E-06	0,001107	4,092082
ENSGALT00000005887	O42253	0,917835	5,862452	8,9E-06	0,00162	3,690165
ENSGALT00000005875	O42253	0,833692	5,685514	1,33E-05	0,002332	3,305084
ENSGALT00000011346	ZBTB16	0,680697	5,573727	1,71E-05	0,002905	3,059971
ENSGALT00000022516	Q5ZJR6	0,58517	5,550892	1,8E-05	0,002962	3,009736
ENSGALT00000005970	NIPAL4	1,188776	5,502679	2,01E-05	0,003203	2,90349
NM_001083359	FOXN4	-0,79827	-5,4845	2,1E-05	0,003238	2,863372
NM_205018	IL8	1,044997	5,4604	2,21E-05	0,003321	2,810126
ENSGALT00000025920	EGR4	1,219336	5,412174	2,47E-05	0,003603	2,703409
ENSGALT00000010268	CH25H	0,726085	5,378136	2,67E-05	0,003787	2,627952
NM_001085359	DUSP1	0,889627	5,121402	4,84E-05	0,006491	2,055435
ENSGALT00000026792	ANGPTL7	0,650946	5,060512	5,57E-05	0,007285	1,918853
ENSGALT00000040970	ENSGALG00000(	1,121197	5,030152	5,98E-05	0,007623	1,85065
NM_204615	TULP1	0,732017	4,950098	7,21E-05	0,008963	1,670508
NM_204776	CCL1	0,760613	4,903664	8,03E-05	0,009751	1,565827

Supplementary Table S4 *. The list of genes which are differently expressed between the sexes based on the ANOVA model (~sex + breed + treatment). The reported values include: transcription ID, gene, log2 fold change (logFC), P.Value, BH adjusted P.Value (adj.P.Val), and B Value. The positive logFC values suggest that the gene in up regulated in red females and negative logFC values suggest up-regulation of the gene in males.*

**Transcript**	**Gene**	**logFC**	**t**	**P.Value**	**adj.P.Val**	**B**
ENSGALT00000036323		7,484286	45,81684	3,96E-22	2,02E-18	40,43073
NM_204191	SPINW	5,899902	42,85835	1,54E-21	3,31E-18	39,19822
ENSGALT00000036331	LOC771385	7,838942	42,33376	1,97E-21	3,31E-18	38,96872
ENSGALT00000029239		7,485095	39,07445	9,99E-21	6,58E-18	37,46132
ENSGALT00000036325	Q5F3T6	7,808679	38,23302	1,55E-20	8,32E-18	37,04772
NM_204688	HINTW	5,966473	38,13744	1,63E-20	8,32E-18	37,00006
ENSGALT00000036330	LOC771385	7,975916	37,00568	3E-20	1,18E-17	36,4249
ENSGALT00000029536	Q5W4Z2	7,40153	36,12614	4,88E-20	1,78E-17	35,96364
ENSGALT00000028320	Q3MMY7	6,048529	33,7727	1,9E-19	6,11E-17	34,66328
ENSGALT00000036320	LOC771438	6,100484	33,75623	1,92E-19	6,11E-17	34,65382
ENSGALT00000036333		7,500844	33,31106	2,51E-19	7,51E-17	34,39617
ENSGALT00000038395		6,70761	29,71251	2,49E-18	6,35E-16	32,16208
ENSGALT00000036342	LOC771406	8,073645	28,73964	4,86E-18	1,18E-15	31,50716
ENSGALT00000036332	LOC771385	8,286217	25,38301	5,79E-17	1,34E-14	29,0521
ENSGALT00000031505	Q98TH7	3,520938	23,85386	1,99E-16	4,22E-14	27,81945
ENSGALT00000023466		1,976571	22,13622	8,71E-16	1,78E-13	26,33562
ENSGALT00000023599	Q9YI73	4,106515	17,45715	8,93E-14	1,75E-11	21,63832
ENSGALT00000021743	A7XMV1	1,637648	17,16876	1,23E-13	2,32E-11	21,3112
ENSGALT00000041374		2,070764	15,58176	7,85E-13	1,43E-10	19,41683
ENSGALT00000036312	Q90VX2	1,122411	14,35896	3,66E-12	6,44E-10	17,83726
ENSGALT00000038812	Q02359	2,815166	12,47877	4,9E-11	8,32E-09	15,17185
ENSGALT00000023597		1,940156	11,88649	1,18E-10	1,94E-08	14,26529
ENSGALT00000023643	ARSK	-0,85113	-11,2023	3,41E-10	5,44E-08	13,17393
ENSGALT00000036314		2,339886	10,88914	5,63E-10	8,7E-08	12,65777
ENSGALT00000023743	MIER3	1,377804	10,55582	9,71E-10	1,41E-07	12,09648
ENSGALT00000023322	LOC431003	1,021747	9,898562	2,95E-09	4,18E-07	10,95229
ENSGALT00000022940	Q9I9P2	0,926983	9,823097	3,36E-09	4,63E-07	10,81764
ENSGALT00000005752	LOC427440	-0,86355	-9,66028	4,47E-09	5,99E-07	10,52479
ENSGALT00000033715		1,101852	9,583314	5,12E-09	6,68E-07	10,38521
ENSGALT00000032784	ATG10	-0,82323	-9,56924	5,24E-09	6,68E-07	10,35962
ENSGALT00000009363	DNAI1	-1,27638	-9,527	5,65E-09	7,03E-07	10,28264
ENSGALT00000023634	C5orf36	-0,98816	-9,42146	6,82E-09	8,28E-07	10,08932
ENSGALT00000024394	SMC5	-0,75298	-9,24207	9,41E-09	1,12E-06	9,757585
ENSGALT00000008522	OVCH2	-3,90176	-9,15961	1,09E-08	1,27E-06	9,603724
ENSGALT00000036316		2,152079	9,130333	1,15E-08	1,31E-06	9,548892
ENSGALT00000042607	SNORA13	-0,70407	-9,069	1,29E-08	1,43E-06	9,433669
NM_001006585	C9orf80	-0,86856	-9,05454	1,32E-08	1,44E-06	9,406435
ENSGALT00000022414	Q6Y2W3	1,313815	8,827755	2,01E-08	2,14E-06	8,9758
ENSGALT00000023755	ERCC8	-0,77846	-8,81287	2,07E-08	2,15E-06	8,947298
ENSGALT00000023967	LOC427191	-0,81113	-8,79241	2,15E-08	2,19E-06	8,908097
ENSGALT00000025179	XRCC4	-0,72832	-8,7729	2,23E-08	2,23E-06	8,870646
NM_001165913	LOC422926	5,288828	8,734726	2,39E-08	2,35E-06	8,797234
ENSGALT00000008519	SRFBP1	-0,83133	-8,54058	3,45E-08	3,31E-06	8,420966

ENSGALT00000024494	CEP78	-0,7962	-8,5321	3,5E-08	3,31E-06	8,404403
ENSGALT00000041534	SNORD72	-0,8034	-8,37363	4,74E-08	4,22E-06	8,093459
ENSGALT00000037675	F172A	-0,83709	-8,37319	4,75E-08	4,22E-06	8,092581
ENSGALT00000031543	GLL9	-4,41365	-8,36706	4,8E-08	4,22E-06	8,080491
ENSGALT00000009323	KIF24	-0,94041	-8,297	5,49E-08	4,67E-06	7,941903
ENSGALT00000023780	SDCCAG10	-0,70171	-8,23819	6,15E-08	5,14E-06	7,825072
ENSGALT00000032827	CNTLN	-0,80939	-8,22109	6,36E-08	5,15E-06	7,791004
ENSGALT00000024368	C9orf93	-0,76864	-8,19856	6,65E-08	5,29E-06	7,746087
ENSGALT00000009330	NUDT2	-0,84729	-8,17591	6,95E-08	5,39E-06	7,700831
ENSGALT00000023609	CETN3	-0,73648	-8,17306	6,98E-08	5,39E-06	7,695136
ENSGALT00000029382		-0,71365	-8,0282	9,27E-08	6,86E-06	7,404161
NM_001001611	GAL9	-4,0001	-8,0272	9,29E-08	6,86E-06	7,402151
ENSGALT00000021979	SPTLC1	-0,62993	-7,92352	1,14E-07	8,3E-06	7,192166
ENSGALT00000042590	SCARNA8	-0,815	-7,87763	1,25E-07	8,96E-06	7,098757
ENSGALT00000025117	LOC427315	-0,77673	-7,85964	1,29E-07	9,12E-06	7,062067
ENSGALT00000038331	C9orf150	-1,05914	-7,85467	1,31E-07	9,12E-06	7,051926
NM_001031429	LOC427269	-0,81499	-7,76112	1,57E-07	1,07E-05	6,860378
ENSGALT00000009688	SLC22A13	-3,55833	-7,72696	1,68E-07	1,12E-05	6,79016
ENSGALT00000025384		1,223517	7,72466	1,69E-07	1,12E-05	6,785415
NM_001006583	MRPL50	-0,66985	-7,71992	1,71E-07	1,12E-05	6,775653
NM_001031611	KIAA0258	-0,76358	-7,64708	1,98E-07	1,28E-05	6,625318
ENSGALT00000032816	C9orf95	-0,72995	-7,62268	2,08E-07	1,32E-05	6,574798
ENSGALT00000042508	snoZ39	-0,73473	-7,57723	2,28E-07	1,43E-05	6,480476
ENSGALT00000031380	MLANA	-3,65625	-7,5302	2,51E-07	1,56E-05	6,382591
ENSGALT00000020565	SLC28A3	-3,16293	-7,45818	2,9E-07	1,78E-05	6,232135
ENSGALT00000025119	LOC427316	-0,84731	-7,4161	3,16E-07	1,92E-05	6,143914
ENSGALT00000024127	C5orf37	-0,68673	-7,35201	3,61E-07	2,16E-05	6,009087
NM_001006582	TMEM175	-0,54941	-7,25154	4,44E-07	2,63E-05	5,796658
ENSGALT00000004819	Q6ZXD0	-0,63994	-7,24074	4,54E-07	2,66E-05	5,773738
ENSGALT00000006940	Q5ZHQ0	-0,67367	-7,22696	4,67E-07	2,7E-05	5,744487
NM_001031448	AMACR	-0,60781	-7,17198	5,23E-07	3E-05	5,627511
ENSGALT00000005321	RAD1	-0,77057	-7,15995	5,36E-07	3,04E-05	5,601853
ENSGALT00000037702	Q5F3N8	-0,75244	-7,14491	5,53E-07	3,1E-05	5,569749
ENSGALT00000024907	FKTN	-0,68574	-7,13926	5,6E-07	3,1E-05	5,557685
ENSGALT00000032868		-0,64876	-7,1136	5,91E-07	3,24E-05	5,502838
ENSGALT00000036319		0,964283	7,091251	6,19E-07	3,36E-05	5,455014
ENSGALT00000028816		-0,7021	-7,06989	6,47E-07	3,47E-05	5,409223
ENSGALT00000024040	MOCS2	-0,74427	-6,9504	8,32E-07	4,42E-05	5,152053
NM_001006580	TMC1	-0,89582	-6,89949	9,26E-07	4,87E-05	5,041924
NM_204378	FANCG	-0,69071	-6,88561	9,54E-07	4,96E-05	5,011846
ENSGALT00000021820	CAPSL	-1,63833	-6,87996	9,65E-07	4,96E-05	4,999575
ENSGALT00000037508	CCDC112	-0,73985	-6,87586	9,74E-07	4,96E-05	4,990681
ENSGALT00000023636		-0,90553	-6,81424	1,11E-06	5,6E-05	4,856772
ENSGALT00000039044		-0,99212	-6,80603	1,13E-06	5,64E-05	4,838875
ENSGALT00000020555	KIF27	-0,60283	-6,75941	1,25E-06	6,09E-05	4,737189
ENSGALT00000020539	RASEF	-0,92947	-6,757	1,25E-06	6,09E-05	4,731939
ENSGALT00000012986	SLC26A4	-1,7289	-6,65242	1,57E-06	7,54E-05	4,502807
ENSGALT00000024477	LOC427259	-0,71014	-6,6389	1,61E-06	7,69E-05	4,473068
ENSGALT00000024051	ROS	-1,85864	-6,60923	1,72E-06	8,12E-05	4,407762
ENSGALT00000005480		-1,69096	-6,60175	1,75E-06	8,18E-05	4,391296

ENSGALT00000023723	SLC38A9	-0,67223	-6,58385	1,82E-06	8,42E-05	4,35182
ENSGALT00000024606		-2,30914	-6,55406	1,94E-06	8,9E-05	4,286051
ENSGALT00000039335		-0,76444	-6,51565	2,11E-06	9,59E-05	4,201109
NM_001031404	LOC426890	-0,65119	-6,50655	2,15E-06	9,69E-05	4,180952
ENSGALT00000034636	MOBKL2B	-0,769	-6,48282	2,26E-06	0,0001	4,128344
ENSGALT00000017720	TMEM161B	-0,71827	-6,48259	2,26E-06	0,0001	4,127825
ENSGALT00000008624	C9orf105	-0,62054	-6,47723	2,29E-06	0,000101	4,115938
ENSGALT00000009335	KIAA1161	-0,74436	-6,44425	2,46E-06	0,000107	4,042693
ENSGALT00000020586	DAPK1	-0,58827	-6,43409	2,51E-06	0,000109	4,020102
NM_205257	ROS1	-1,56225	-6,36637	2,91E-06	0,000124	3,869223
ENSGALT00000037592	LOC427362	-0,78025	-6,3663	2,91E-06	0,000124	3,869076
ENSGALT00000009958	LRRC18	-1,67956	-6,23658	3,87E-06	0,000163	3,578558
ENSGALT00000039461		-0,74094	-6,23181	3,91E-06	0,000163	3,567856
NM_205467	LOC396454	-0,71621	-6,2289	3,94E-06	0,000163	3,561312
ENSGALT00000034419	TLN1	-0,75099	-6,17294	4,46E-06	0,000183	3,435352
ENSGALT00000024385	PTAR1	-0,77791	-6,15848	4,6E-06	0,000188	3,402742
ENSGALT00000025118	LOC768418	-0,71876	-6,14197	4,77E-06	0,000193	3,365486
ENSGALT00000009397	IL11RA	-0,84299	-6,12965	4,9E-06	0,000197	3,337646
NM_001167729	TBCA	-0,61567	-6,10423	5,19E-06	0,000207	3,280209
ENSGALT00000023611	POLR3G	-0,58453	-6,09796	5,26E-06	0,000208	3,266023
ENSGALT00000011090	Q92062	-0,94742	-6,08201	5,45E-06	0,000214	3,229922
ENSGALT00000037571	Q5ZJI2	-0,73736	-6,07417	5,55E-06	0,000216	3,212163
ENSGALT00000023909	CMYA5	-0,78543	-6,05305	5,81E-06	0,000224	3,16429
ENSGALT00000037916	LYRM7	-0,70587	-6,04945	5,86E-06	0,000225	3,156126
ENSGALT00000037797		-0,81487	-6,03981	5,99E-06	0,000228	3,134263
ENSGALT00000030986	Q98TX8	-0,91641	-6,01732	6,29E-06	0,000238	3,083209
ENSGALT00000024645	NUDT12	-0,75697	-5,98886	6,71E-06	0,000251	3,018541
NM_204183	POLK	-0,55626	-5,92747	7,69E-06	0,000284	2,878733
NM_001098609	PCDH8	1,154319	5,897609	8,22E-06	0,000302	2,810582
ENSGALT00000028205	TUSC1	-0,64778	-5,88887	8,39E-06	0,000305	2,790628
ENSGALT00000023969	FBXO4	-0,71722	-5,87845	8,59E-06	0,00031	2,766803
ENSGALT00000009698	XIRP1	-1,77264	-5,86697	8,81E-06	0,000316	2,740557
ENSGALT00000005754		-0,85121	-5,83252	9,52E-06	0,000339	2,661733
ENSGALT00000023926	JMY	-0,65469	-5,82739	9,63E-06	0,000341	2,649973
ENSGALT00000026593	LOC421965	-2,35365	-5,82042	9,78E-06	0,000344	2,634005
ENSGALT00000023789	CENPK	-0,64835	-5,77861	1,07E-05	0,000375	2,538111
ENSGALT00000032821	ANXA1	-0,61947	-5,76458	1,11E-05	0,00038	2,505909
ENSGALT00000036515		0,823592	5,704014	1,27E-05	0,000429	2,366612
ENSGALT00000024770	PIGG	-0,69418	-5,69667	1,29E-05	0,000434	2,349699
ENSGALT00000029745	LOC427060	0,436244	5,691114	1,31E-05	0,000436	2,336901
ENSGALT00000023749	RAB3C	-0,45779	-5,68665	1,32E-05	0,000438	2,326611
ENSGALT00000038882	Q8QGH0	-0,61308	-5,66305	1,4E-05	0,000459	2,272204
ENSGALT00000024442	AL1A1	-0,77733	-5,64199	1,46E-05	0,000475	2,22362
ENSGALT00000041348	TMEM213	-1,60397	-5,63502	1,49E-05	0,000477	2,207514
ENSGALT00000024125	ANKDD1B	-0,77903	-5,6348	1,49E-05	0,000477	2,207016
ENSGALT00000033768	Q7T269	2,692542	5,620091	1,54E-05	0,00049	2,173044
ENSGALT00000004529	MALT1	-0,76526	-5,60693	1,59E-05	0,000497	2,142631
ENSGALT00000038227		-0,57268	-5,60556	1,59E-05	0,000497	2,139455
ENSGALT00000023623	ARRDC3	-0,69406	-5,60555	1,59E-05	0,000497	2,139445
ENSGALT00000035384		-0,7082	-5,59706	1,62E-05	0,000504	2,119814

ENSGALT00000008658	WDR32	-0,66426	-5,58372	1,67E-05	0,000513	2,088952
ENSGALT00000039281	Q9PST0	-0,73027	-5,58122	1,68E-05	0,000513	2,083177
ENSGALT00000030190	CLDN10	-1,48705	-5,58065	1,68E-05	0,000513	2,081847
ENSGALT00000021704	CP19A	2,995781	5,579074	1,69E-05	0,000513	2,078208
ENSGALT00000005943	OSMR	-0,53286	-5,56673	1,74E-05	0,000524	2,049625
ENSGALT00000018624	ISK1L	-2,19608	-5,55789	1,77E-05	0,000529	2,029172
ENSGALT00000014609	VWA2	-1,01811	-5,52943	1,89E-05	0,000557	1,963222
ENSGALT00000032302	AKAP14	-0,60989	-5,50821	1,99E-05	0,000578	1,91401
ENSGALT00000028609	U2	-0,71024	-5,50665	1,99E-05	0,000578	1,910387
ENSGALT00000036504		0,783312	5,505875	2E-05	0,000578	1,908597
ENSGALT00000010598	Q4GWZ4	-1,03499	-5,50013	2,02E-05	0,000582	1,895274
ENSGALT00000038913	XIRP1	-1,72333	-5,49208	2,06E-05	0,00059	1,876581
ENSGALT00000012816	SPAG6	-1,28106	-5,47703	2,13E-05	0,000607	1,841636
NM_001001761	CYP19A1	3,145731	5,453006	2,25E-05	0,000634	1,785814
ENSGALT00000024223	FCHO2	-0,60064	-5,44781	2,28E-05	0,000639	1,773732
ENSGALT00000039103		-0,93088	-5,43408	2,35E-05	0,000652	1,741799
NM_001031431	TRIM14	-0,4847	-5,39702	2,56E-05	0,000706	1,65556
ENSGALT00000040918	LOC769773	0,473399	5,383162	2,64E-05	0,000723	1,623267
ENSGALT00000042580	SNORA66	-0,71173	-5,38176	2,65E-05	0,000723	1,620005
ENSGALT00000032871	DIMT1L	-0,56049	-5,34994	2,85E-05	0,000763	1,545808
ENSGALT00000013799	LRGUK	-0,91404	-5,34924	2,86E-05	0,000763	1,544192
ENSGALT00000037626	KIAA1958	-0,83363	-5,30327	3,18E-05	0,000844	1,436888
ENSGALT00000009820	GPC3	1,209461	5,29048	3,27E-05	0,000862	1,406999
ENSGALT00000034649	LOC769382	-0,73842	-5,28951	3,28E-05	0,000862	1,404726
ENSGALT00000008587	ZBTB5	-0,80853	-5,2833	3,33E-05	0,00087	1,390209
ENSGALT00000029431		-0,90505	-5,26557	3,47E-05	0,000897	1,348775
ENSGALT00000019742	PITX2	-1,25972	-5,25757	3,53E-05	0,000909	1,330047
ENSGALT00000014656	LOC768975	-0,84414	-5,23963	3,68E-05	0,000938	1,288093
ENSGALT00000012751	PAPOLG	1,015915	5,22576	3,8E-05	0,000961	1,255618
ENSGALT00000037523		-0,81357	-5,22051	3,85E-05	0,000961	1,24332
NM_205446	TPM3	-0,61953	-5,21159	3,93E-05	0,000976	1,222439
ENSGALT00000006843	DNAH1	-1,27734	-5,18758	4,15E-05	0,001027	1,166175
NM_204407	GRPR	0,681248	5,183337	4,19E-05	0,00103	1,15624
NM_001040463	OLFM4	-1,79881	-5,18231	4,2E-05	0,00103	1,153834
NM_001079742	STARD4	-0,55249	-5,17179	4,3E-05	0,00105	1,129177
ENSGALT00000010884	Q9I8D9	-0,62448	-5,1655	4,37E-05	0,00106	1,114422
ENSGALT00000034619	APTX	-0,99083	-5,15125	4,51E-05	0,001091	1,081
ENSGALT00000022662	EYA4	-1,25401	-5,14788	4,55E-05	0,001094	1,073093
ENSGALT00000021504	LOC418189	-1,6878	-5,13811	4,65E-05	0,001114	1,050172
ENSGALT00000024587	PPIL6	-2,23941	-5,11823	4,87E-05	0,001161	1,003507
ENSGALT00000039654	KIAA0427	-0,99408	-5,11546	4,91E-05	0,001163	0,996997
ENSGALT00000025304	KIAA0368	-0,70907	-5,0975	5,11E-05	0,001207	0,954833
ENSGALT00000025702	CDH17	-1,64056	-5,08877	5,22E-05	0,001226	0,93432
ENSGALT00000024058	SPINZ	-0,65882	-5,0778	5,35E-05	0,001252	0,908541
ENSGALT00000042554	SNORD121A	-0,71539	-5,06972	5,46E-05	0,00127	0,889555
ENSGALT00000039998	LOC771321	-1,89771	-5,03782	5,88E-05	0,001362	0,814545
ENSGALT00000026546	GREB1	0,506155	5,029152	6E-05	0,001383	0,794146
NM_001001746	ST8SIA3	-0,50696	-5,02466	6,06E-05	0,001391	0,783568
ENSGALT00000006516	Q8AYE5	-1,15188	-5,01571	6,19E-05	0,001414	0,762505
NM_204577	ALDH1A1	-0,7803	-5,00861	6,29E-05	0,001431	0,745796

ENSGALT00000024126	BET3L	0,624182	5,006541	6,32E-05	0,001432	0,740935
ENSGALT00000032847	Q8AWW1	-0,44515	-5,00045	6,41E-05	0,001446	0,726594
ENSGALT00000024804	Q802E5	-0,57037	-4,99673	6,47E-05	0,001451	0,717833
ENSGALT00000026109	COL22A1	0,744145	4,99279	6,53E-05	0,001451	0,708565
ENSGALT00000005481		-0,73502	-4,9915	6,54E-05	0,001451	0,705529
ENSGALT00000039311	LOC770114	1,022462	4,984039	6,66E-05	0,00147	0,687957
ENSGALT00000034941	SLC13A2	-1,07183	-4,97705	6,77E-05	0,001487	0,671495
ENSGALT00000039996	LOC771321	-1,95495	-4,96619	6,94E-05	0,001519	0,645927
ENSGALT00000031598	C2orf50	-1,24535	-4,95974	7,05E-05	0,001535	0,630724
ENSGALT00000033270		-0,60909	-4,95004	7,21E-05	0,001563	0,607866
NM_205364	CHRNA6	-0,54795	-4,94855	7,23E-05	0,001563	0,604352
NM_204854	PRLR	-0,68043	-4,93759	7,42E-05	0,00159	0,578536
ENSGALT00000031399	LOC428291	0,426502	4,935439	7,46E-05	0,001591	0,573458
ENSGALT00000038855		-2,14551	-4,93358	7,49E-05	0,001591	0,569073
NM_001006587	FANCC	-0,65494	-4,91641	7,8E-05	0,001643	0,528592
ENSGALT00000005551	C9orf9	-0,86562	-4,91191	7,88E-05	0,001653	0,517981
ENSGALT00000040884	LOC770639	0,44681	4,907649	7,96E-05	0,001659	0,507937
ENSGALT00000025306	SMC2	-0,63843	-4,90694	7,97E-05	0,001659	0,506271
ENSGALT00000011587	FAM3D	-0,99489	-4,90193	8,07E-05	0,001672	0,494462
ENSGALT00000040310	PAPOLG	0,95191	4,885245	8,39E-05	0,001731	0,455094
ENSGALT00000025271	HNF4G	-1,09889	-4,85822	8,93E-05	0,001837	0,39132
ENSGALT00000005897	Q9IAM2	-0,44161	-4,84848	9,14E-05	0,001871	0,368325
ENSGALT00000011997	ARMC4	-0,81088	-4,8465	9,18E-05	0,001873	0,36367
ENSGALT00000037521		-0,85695	-4,84317	9,25E-05	0,001876	0,355797
ENSGALT00000006668	UGT1A9	-1,44975	-4,84071	9,31E-05	0,001876	0,350001
ENSGALT00000004736	WDR69	-1,40923	-4,84068	9,31E-05	0,001876	0,349925
NM_001039098	ZP2	2,430694	4,817557	9,83E-05	0,001965	0,295328
ENSGALT00000023845		-0,58069	-4,81096	9,98E-05	0,001987	0,27975
ENSGALT00000024328	ASAH3L	-0,5064	-4,78877	0,000105	0,002074	0,227338
ENSGALT00000019560	MYOZ2	0,725995	4,787812	0,000105	0,002074	0,225071
ENSGALT00000029057	gga-mir-101	-0,71961	-4,77603	0,000108	0,002121	0,197225
ENSGALT00000027127	Q8AV27	1,56852	4,77498	0,000109	0,002121	0,194753
ENSGALT00000024332	DENND4C	-0,66843	-4,76679	0,000111	0,002154	0,175411
ENSGALT00000037524		-0,78213	-4,76077	0,000112	0,002176	0,161172
ENSGALT00000024816	SLC26A1	-1,06539	-4,74924	0,000115	0,002227	0,133919
ENSGALT00000005445		-1,16609	-4,73508	0,000119	0,002294	0,100461
ENSGALT00000025057	C9orf125	-0,63607	-4,72853	0,000121	0,002321	0,084968
ENSGALT00000026066	RSPH1	-1,05126	-4,72544	0,000122	0,002324	0,077674
ENSGALT00000037926		2,466424	4,723622	0,000122	0,002324	0,07337
ENSGALT00000019315	SLC6A20	-0,90189	-4,72313	0,000123	0,002324	0,072217
ENSGALT00000025241	PALM2	-0,5408	-4,7196	0,000124	0,002331	0,063858
ENSGALT00000009756	LOC416935	-1,7476	-4,71868	0,000124	0,002331	0,061688
ENSGALT00000037928		2,415788	4,707927	0,000127	0,002379	0,036261
ENSGALT00000037079		-1,17554	-4,70698	0,000127	0,002379	0,034033
ENSGALT00000010857	RTDR1	-0,61274	-4,7011	0,000129	0,002398	0,020123
ENSGALT00000009569	LOC416931	0,573113	4,700406	0,000129	0,002398	0,018479
ENSGALT00000018105	WDR78	-0,92028	-4,69092	0,000132	0,002429	-0,00394
ENSGALT00000013404	GRAMD3	-0,51989	-4,69037	0,000132	0,002429	-0,00526
ENSGALT00000024303	PTPLAD2	-0,48542	-4,68991	0,000133	0,002429	-0,00633
NM_001031130	NPY5R	-0,69049	-4,68874	0,000133	0,002429	-0,00911

ENSGALT00000008150	SLC5A10	-0,72351	-4,66664	0,00014	0,002541	-0,06137
ENSGALT00000024425	WDR52	-0,94693	-4,64659	0,000147	0,002654	-0,10879
ENSGALT00000020596	Q5ZK89	-0,58469	-4,6254	0,000154	0,00278	-0,15894
ENSGALT00000021514	PYROXD1	0,45279	4,610408	0,00016	0,002869	-0,1944
ENSGALT00000040921	LOC431350	0,485056	4,608784	0,00016	0,00287	-0,19824
ENSGALT00000039084	Q6WV23	1,959344	4,595465	0,000166	0,002945	-0,22976
ENSGALT00000030657	LOC772233	-0,79996	-4,59403	0,000166	0,002945	-0,23315
ENSGALT00000042394	gga-mir-1713	-1,29986	-4,59335	0,000166	0,002945	-0,23475
ENSGALT00000015921	CLRN3	-1,48564	-4,57577	0,000173	0,003059	-0,27637
NM_205025	MAFA	0,739106	4,57317	0,000174	0,003064	-0,28251
ENSGALT00000040898	LOC770152	0,480816	4,572139	0,000175	0,003064	-0,28496
ENSGALT00000041087		-0,74354	-4,56477	0,000178	0,003107	-0,30239
ENSGALT00000013526	CCDC146	-0,92928	-4,55942	0,00018	0,003131	-0,31506
ENSGALT00000030736	SLC26A3	-2,37146	-4,55739	0,000181	0,003131	-0,31986
ENSGALT00000040913	LOC770241	0,444605	4,557225	0,000181	0,003131	-0,32025
ENSGALT00000008429	TEKT4	-1,3711	-4,55516	0,000182	0,003131	-0,32514
ENSGALT00000017472	DIO1	-1,09751	-4,55437	0,000182	0,003131	-0,32701
ENSGALT00000021671		-1,96746	-4,54865	0,000185	0,003158	-0,34054
ENSGALT00000024818	ACHA6	-0,53204	-4,54789	0,000185	0,003158	-0,34234
ENSGALT00000039782		-1,82044	-4,54234	0,000188	0,003189	-0,35547
ENSGALT00000026779	C2orf39	-0,73343	-4,53052	0,000193	0,003268	-0,38345
ENSGALT00000025905	DPYS	-2,25358	-4,52516	0,000195	0,003298	-0,39613
NM_001159371	HYDIN	-1,07413	-4,52369	0,000196	0,003299	-0,39961
ENSGALT00000009334	LOC427400	-0,99226	-4,52091	0,000197	0,00331	-0,4062
ENSGALT00000006974	KCNJ16	-2,11855	-4,51738	0,000199	0,003326	-0,41454
ENSGALT00000035385	LOC768734	-0,52795	-4,50545	0,000205	0,003407	-0,44277
ENSGALT00000013509	FBXL13	-1,02591	-4,50317	0,000206	0,003407	-0,44818
ENSGALT00000006527	FAM81A	-0,7345	-4,49835	0,000208	0,003434	-0,45958
ENSGALT00000015083	HOXD10	-1,762	-4,49448	0,00021	0,003454	-0,46873
ENSGALT00000013161	SYPL1	1,183924	4,483891	0,000215	0,003529	-0,4938
ENSGALT00000039152		-1,02065	-4,48267	0,000216	0,003529	-0,49668
ENSGALT00000027045	VWA3B	-1,42896	-4,47538	0,00022	0,003568	-0,51395
ENSGALT00000016104	LOC771576	-0,84696	-4,45604	0,00023	0,003722	-0,55971
ENSGALT00000039412	CCDC78	-1,17078	-4,45415	0,000231	0,003722	-0,56417
ENSGALT00000030852	SLC7A13	-2,22559	-4,45341	0,000231	0,003722	-0,56592
ENSGALT00000020529		-0,62197	-4,45184	0,000232	0,003724	-0,56965
ENSGALT00000024224	TMEM171	-0,54142	-4,4182	0,000251	0,004019	-0,64925
ENSGALT00000033185	LOC770114	0,936581	4,41605	0,000253	0,004027	-0,65433
ENSGALT00000038691	HXD4	-1,96885	-4,40654	0,000259	0,004106	-0,67684
ENSGALT00000016271	LOC417848	0,790784	4,404329	0,00026	0,004114	-0,68206
ENSGALT00000034111	C15orf26	-1,16225	-4,39614	0,000265	0,004182	-0,70144
ENSGALT00000005993	NELL1	0,687532	4,388717	0,00027	0,004242	-0,719
ENSGALT00000018136	LRRIQ1	-1,6306	-4,3752	0,000278	0,004367	-0,75098
ENSGALT00000015766	KCNK2	1,068119	4,373896	0,000279	0,004367	-0,75405
ENSGALT00000010703	UPB1	-0,94849	-4,37134	0,000281	0,00438	-0,76011
ENSGALT00000023947	C7	-0,43234	-4,37004	0,000282	0,00438	-0,76317
NM_207177	HOXD8	-1,55863	-4,36057	0,000288	0,004465	-0,78558
ENSGALT00000027840	CCDC67	-1,34443	-4,35724	0,00029	0,004487	-0,79346
ENSGALT00000039495	FHAD1	-0,81142	-4,35214	0,000294	0,004515	-0,8055
ENSGALT00000039315		-0,74379	-4,35203	0,000294	0,004515	-0,80578

ENSGALT00000031104	LOC776358	0,426174	4,348123	0,000297	0,004531	-0,81501
ENSGALT00000016949	VDHAP	-1,60893	-4,34799	0,000297	0,004531	-0,81534
ENSGALT00000011701		-1,46067	-4,33472	0,000306	0,004641	-0,8467
ENSGALT00000017091		-0,90653	-4,33356	0,000307	0,004641	-0,84945
ENSGALT00000005607	C9orf171	-1,35959	-4,3334	0,000307	0,004641	-0,84984
ENSGALT00000013926	MDH1B	-0,62029	-4,33283	0,000308	0,004641	-0,85118
ENSGALT00000009885	MYOT	0,560751	4,315648	0,00032	0,004819	-0,8918
ENSGALT00000038490	TNPO1	-0,49991	-4,31179	0,000323	0,004849	-0,90093
NM_001167752	MB	0,713013	4,305823	0,000328	0,004903	-0,91503
ENSGALT00000024049	HSPB3	-0,54311	-4,2993	0,000333	0,004953	-0,93046
ENSGALT00000024491	GNA14	-1,10726	-4,29905	0,000333	0,004953	-0,93104
ENSGALT00000006672	UGT1A9	-1,79551	-4,29625	0,000335	0,004972	-0,93765
ENSGALT00000012287	LOC416086	-1,59674	-4,28929	0,000341	0,005036	-0,9541
NM_001130744	WNT11B	0,489955	4,287354	0,000343	0,005036	-0,95868
ENSGALT00000022838	MUC1	1,071142	4,287115	0,000343	0,005036	-0,95925
ENSGALT00000026893	NEIL2	1,567766	4,2839	0,000345	0,00506	-0,96685
ENSGALT00000031172	C3orf48	-0,71111	-4,28109	0,000348	0,005079	-0,97349
ENSGALT00000035047	GJB5	0,690972	4,276109	0,000352	0,005117	-0,98526
NM_205482	HAPLN1	0,783813	4,274897	0,000353	0,005117	-0,98812
ENSGALT00000041147		-0,98118	-4,27338	0,000354	0,005117	-0,9917
ENSGALT00000026640	GPR64	-0,63179	-4,27317	0,000354	0,005117	-0,99222
ENSGALT00000015076	KCNK18	-1,03294	-4,27192	0,000355	0,005117	-0,99517
ENSGALT00000025178	KISHA	-0,46646	-4,26681	0,00036	0,005165	-1,00723
ENSGALT00000031284	LOC770010	-1,96289	-4,26515	0,000361	0,005165	-1,01115
NM_001171768	CBLN2	1,049447	4,26442	0,000362	0,005165	-1,01288
ENSGALT00000027388	A5Y5L6	0,503632	4,260743	0,000365	0,005195	-1,02157
NM_204976	LOC395824	-1,88613	-4,24929	0,000375	0,005314	-1,04863
ENSGALT00000010095	PABPN1	1,843219	4,247936	0,000376	0,005314	-1,05182
NM_205079	FSHR	1,042569	4,247531	0,000376	0,005314	-1,05278
ENSGALT00000038690		-0,90207	-4,24541	0,000378	0,005314	-1,05779
NM_001098853	TBPL2	2,351653	4,245345	0,000378	0,005314	-1,05794
ENSGALT00000030016	LOC426842	0,368155	4,242097	0,000381	0,005327	-1,06562
ENSGALT00000037474	MYB	-0,82999	-4,24177	0,000382	0,005327	-1,0664
NM_001031509	MELK	-0,56453	-4,23949	0,000384	0,005329	-1,07177
ENSGALT00000006778	TMIGD1	-1,21716	-4,22619	0,000396	0,00547	-1,10317
ENSGALT00000016056	LOC425525	0,512297	4,224929	0,000397	0,005471	-1,10616
NM_205207	MFI2	-0,67006	-4,20435	0,000417	0,005729	-1,15475
ENSGALT00000018474	NEK10	-1,34412	-4,20055	0,000421	0,005765	-1,16372
ENSGALT00000023931	A2TH16	-0,80843	-4,19935	0,000422	0,005766	-1,16654
ENSGALT00000020569	AGTPBP1	-0,44554	-4,1956	0,000426	0,005793	-1,17541
ENSGALT00000033904	MYOC	0,594175	4,195068	0,000426	0,005793	-1,17666
ENSGALT00000016484	TTC6	-1,52446	-4,19342	0,000428	0,0058	-1,18055
ENSGALT00000037668		-0,66489	-4,19226	0,000429	0,005801	-1,1833
ENSGALT00000006341	IQCA	-1,05408	-4,18777	0,000434	0,005842	-1,19389
ENSGALT00000025150	RASGRF2	-0,69927	-4,18701	0,000434	0,005842	-1,19569
ENSGALT00000023011	RIBC2	-1,83771	-4,18291	0,000439	0,005884	-1,20535
ENSGALT00000039987		-1,07581	-4,1793	0,000442	0,005919	-1,21386
ENSGALT00000040923	LOC431352	0,408279	4,175636	0,000446	0,005955	-1,22251
ENSGALT00000027326	SLITRK1	0,63996	4,16542	0,000457	0,006078	-1,24662
ENSGALT00000040697	AGBL2	-1,53571	-4,16472	0,000458	0,006078	-1,24827

ENSGALT00000036418	CNTN5	0,615468	4,144666	0,00048	0,006357	-1,29556
ENSGALT00000021527	SLC6A18	-2,7807	-4,13327	0,000493	0,006497	-1,32244
ENSGALT00000023569	ACTBL2	2,284033	4,129383	0,000498	0,006526	-1,3316
ENSGALT00000014637	LOC428555	-1,17703	-4,12923	0,000498	0,006526	-1,33197
ENSGALT00000033380		1,270401	4,127987	0,000499	0,006528	-1,33489
ENSGALT00000039652	LOC771291	-1,70505	-4,12614	0,000502	0,00654	-1,33925
ENSGALT00000023689	ZBTB7C	-0,54916	-4,12502	0,000503	0,006541	-1,34187
ENSGALT00000013947	DYTN	-0,71401	-4,12039	0,000508	0,006596	-1,35279
ENSGALT00000014086	HRG	-1,14567	-4,11565	0,000514	0,006653	-1,36397
ENSGALT00000038129	Q9PT87	-0,83045	-4,11211	0,000519	0,006688	-1,37231
ENSGALT00000027076	LOC418707	1,893321	4,111318	0,000519	0,006688	-1,37417
NM_001001299		-1,4081	-4,10911	0,000522	0,006706	-1,37936
ENSGALT00000008427	SGPP2	0,410628	4,102114	0,000531	0,006801	-1,39585
ENSGALT00000038689	HOXD10	-1,51739	-4,10002	0,000534	0,006815	-1,40078
ENSGALT00000037517		-0,6922	-4,0991	0,000535	0,006815	-1,40295
ENSGALT00000009834		-1,55139	-4,09371	0,000542	0,006867	-1,41565
ENSGALT00000016931	FAM194A	-0,74865	-4,09366	0,000542	0,006867	-1,41577
ENSGALT00000018269	SMOC2	0,708354	4,092699	0,000543	0,006867	-1,41803
ENSGALT00000025190	VCAN	-0,86935	-4,08127	0,000558	0,007038	-1,44494
ENSGALT00000037473	MYB	-0,87247	-4,07951	0,00056	0,00705	-1,44908
ENSGALT00000037538	DMXL1	-0,71258	-4,07634	0,000564	0,007086	-1,45656
ENSGALT00000016904		0,99958	4,071414	0,000571	0,007151	-1,46815
ENSGALT00000017248	KCNK10	0,562125	4,066976	0,000577	0,007201	-1,4786
ENSGALT00000034591	LOC769055	-0,54608	-4,06638	0,000578	0,007201	-1,48
ENSGALT00000009874	LOC427505	-0,84433	-4,06177	0,000584	0,007262	-1,49084
ENSGALT00000040912	LOC770241	0,370887	4,052939	0,000596	0,007398	-1,51163
ENSGALT00000040911	LOC770362	0,321448	4,051128	0,000599	0,007411	-1,51589
NM_204106	TBX22	-1,45122	-4,04823	0,000603	0,00744	-1,52271
ENSGALT00000009548	SLC5A11	-1,77047	-4,04745	0,000604	0,00744	-1,52454
NM_001001901	CYP17A1	1,471595	4,041698	0,000612	0,007518	-1,53808
ENSGALT00000039880		0,631144	4,041012	0,000613	0,007518	-1,53969
ENSGALT00000040905	LOC770241	0,4351	4,039138	0,000616	0,007533	-1,5441
ENSGALT00000040367	Q6R6I2	0,492625	4,037654	0,000618	0,007541	-1,54759
NM_001012608	F2RL1	-1,24062	-4,02907	0,000631	0,007678	-1,56777
ENSGALT00000004309	CCDC57	-0,3385	-4,02492	0,000637	0,007734	-1,57754
ENSGALT00000018523	CCDC108	-0,74405	-4,02399	0,000639	0,007734	-1,57972
NM_204462	KCNA10	0,464666	4,022679	0,000641	0,007739	-1,5828
ENSGALT00000020216		-1,05427	-4,02004	0,000645	0,007769	-1,58901
ENSGALT00000038528	Q76CF0	-0,47046	-4,01456	0,000653	0,007833	-1,60189
NM_001006576	DEPDC1B	-0,39856	-4,00904	0,000662	0,007918	-1,61486
ENSGALT00000012704		0,397447	4,003028	0,000671	0,008012	-1,62898
ENSGALT00000024893	GRIK2	-0,70997	-3,99836	0,000679	0,008082	-1,63995
ENSGALT00000040890	LOC770562	0,349779	3,994831	0,000684	0,008112	-1,64824
ENSGALT00000007161	DNAI2	-1,41714	-3,99476	0,000684	0,008112	-1,6484
NM_204871	IL6ST	-0,64796	-3,99384	0,000686	0,008112	-1,65058
ENSGALT00000040764	LAD1	-1,7585	-3,9898	0,000692	0,008171	-1,66006
ENSGALT00000040907	LOC431276	0,463858	3,988751	0,000694	0,008172	-1,66252
ENSGALT00000039994		-1,70188	-3,9876	0,000696	0,008176	-1,66522
ENSGALT00000038576	Q5F4D0	-1,31783	-3,98654	0,000698	0,008177	-1,66772
ENSGALT00000009253	BIRC7	1,077875	3,983797	0,000702	0,008202	-1,67416

ENSGALT00000039633	UBXD3	-0,63857	-3,98331	0,000703	0,008202	-1,6753
ENSGALT00000014340	IQUB	-0,52267	-3,98189	0,000705	0,008211	-1,67863
ENSGALT00000028225	LOC426893	-0,63497	-3,96918	0,000727	0,008423	-1,70847
NM_001012997	CEL	0,615418	3,969134	0,000727	0,008423	-1,70858
ENSGALT00000025298		-0,78314	-3,9681	0,000729	0,008423	-1,71101
NM_204818	GSTA3	-1,45445	-3,96707	0,000731	0,008423	-1,71343
NR_035209	MIR1713	-1,14543	-3,96628	0,000732	0,008423	-1,71527
ENSGALT00000026713	C7ECT7	-0,78679	-3,95925	0,000744	0,008543	-1,73177
ENSGALT00000007066	CPSF4L	0,773129	3,95766	0,000747	0,008543	-1,73551
ENSGALT00000005023	DNAH10	-0,99095	-3,95745	0,000747	0,008543	-1,736
ENSGALT00000031575	PFN4	-0,81484	-3,95047	0,00076	0,008656	-1,75237
ENSGALT00000014512	LOC769060	1,386441	3,94999	0,000761	0,008656	-1,7535
ENSGALT00000014374	HAO1	-1,07076	-3,9482	0,000764	0,008674	-1,7577
ENSGALT00000023900	Q91002	0,689792	3,94629	0,000767	0,008694	-1,76218
ENSGALT00000040906	LOC769877	0,330597	3,9448	0,00077	0,008705	-1,76567
ENSGALT00000008112	MUPCDH	-1,37741	-3,93993	0,000779	0,008786	-1,7771
ENSGALT00000024915	ZNF462	-0,43611	-3,93074	0,000796	0,008959	-1,79863
ENSGALT00000006925	PDE8B	-0,57057	-3,92656	0,000804	0,009025	-1,80844
ENSGALT00000040780	LOC408038	1,133046	3,925779	0,000805	0,009025	-1,81026
ENSGALT00000037679	PITX2	-0,94621	-3,92287	0,000811	0,009067	-1,81707
ENSGALT00000007433	KPNA7	1,230876	3,921898	0,000813	0,009068	-1,81936
ENSGALT00000027077	HIST1H2AH	0,934501	3,913535	0,000829	0,009229	-1,83895
ENSGALT00000023726		0,508459	3,908427	0,000839	0,00932	-1,85092
ENSGALT00000024486	LOC418363	1,323334	3,905915	0,000844	0,009356	-1,8568
ENSGALT00000040891	LOC770241	0,351751	3,903412	0,000849	0,009391	-1,86266
ENSGALT00000019865	NPSR1	-0,73218	-3,89506	0,000866	0,009557	-1,88222
NM_204429	CHIA	0,731541	3,892241	0,000872	0,0096	-1,88882
NM_001195689	C9orf100	-0,60331	-3,89133	0,000874	0,0096	-1,89096
NM_206980	GCM1	0,885474	3,889074	0,000878	0,00963	-1,89623
ENSGALT00000006581	TDRD5	1,342709	3,887745	0,000881	0,00964	-1,89934
ENSGALT00000034839	FOXJ1	-1,7364	-3,8864	0,000884	0,00965	-1,90248
ENSGALT00000020126	C6orf103	-1,11663	-3,87658	0,000905	0,009855	-1,92546
ENSGALT00000034393	CCDC153	-1,00439	-3,87525	0,000908	0,00986	-1,92858
ENSGALT00000033221	LOC424557	-1,11171	-3,87455	0,000909	0,00986	-1,93021
ENSGALT00000025824	RGS22	-1,26292	-3,8731	0,000912	0,009863	-1,93361
ENSGALT00000041052	Q6X0K9	0,835277	3,872654	0,000913	0,009863	-1,93465
ENSGALT00000016514	ANKRD15	-0,38149	-3,86679	0,000926	0,009975	-1,94836
